# Supplementary material for: Factors associated with the referral of children with severe illnesses at primary care level in Ethiopia: a cross-sectional study
Source: BMJ Open. 2021 Jun 9;11(6):e047640. doi: 10.1136/bmjopen-2020-047640 (PMC8194336; doi:10.1136/bmjopen-2020-047640)
Supplement: Supplementary data [file bmjopen-2020-047640supp001.pdf]

List of supplementary figures

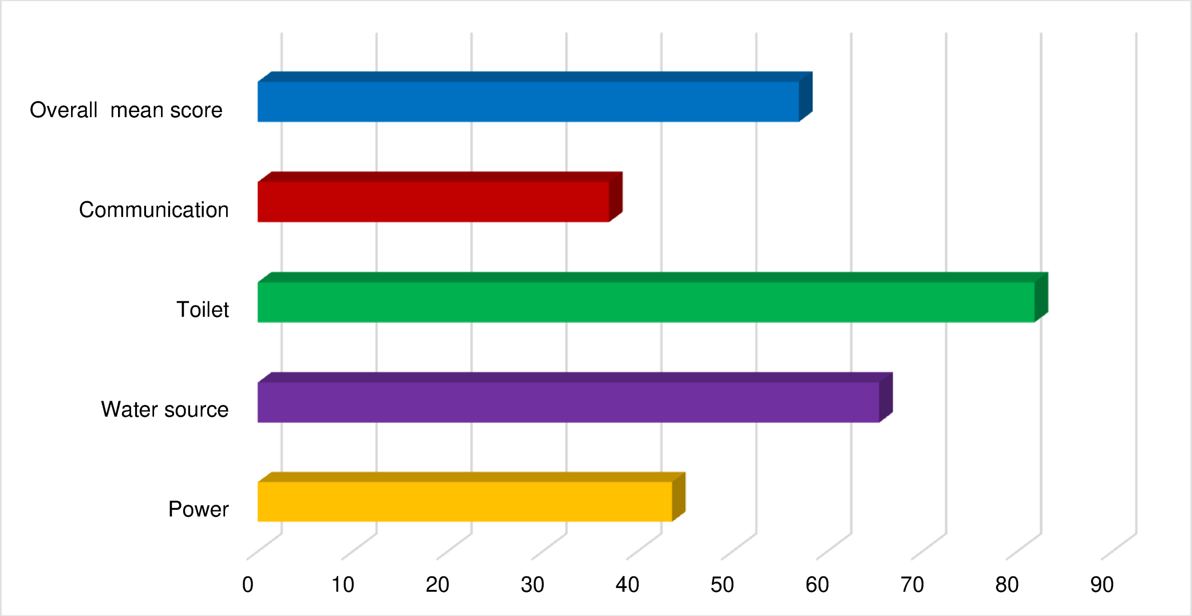

Supplementary figure 1: Percentages of mean score of basic amenities by tracer items

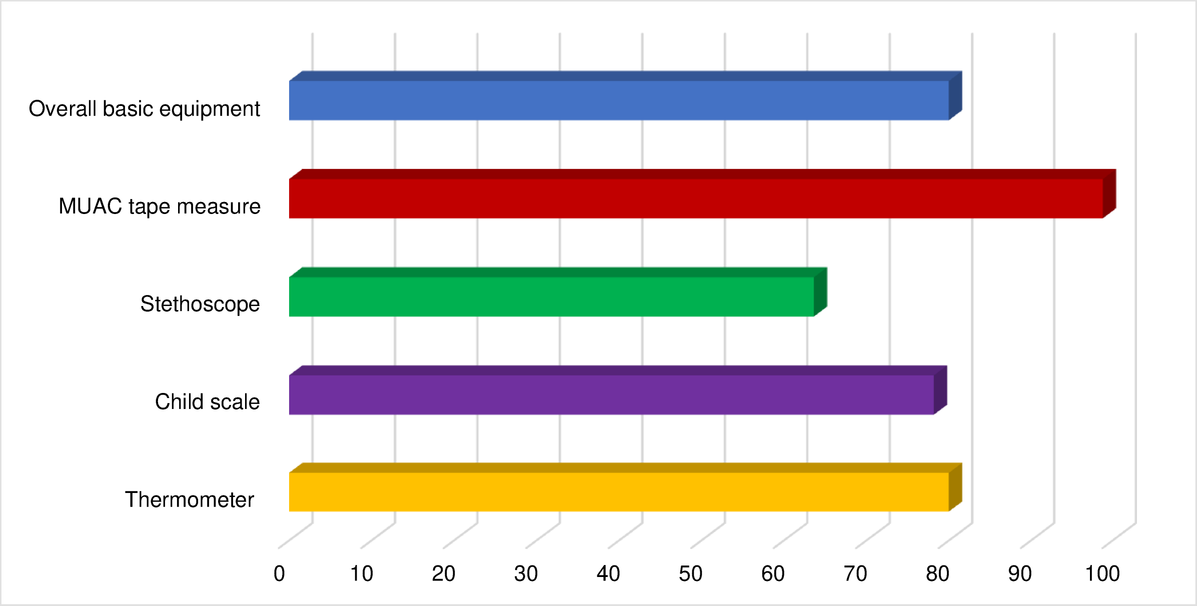

Supplementary figure 2: Percentages of mean score of basic equipment by tracer items

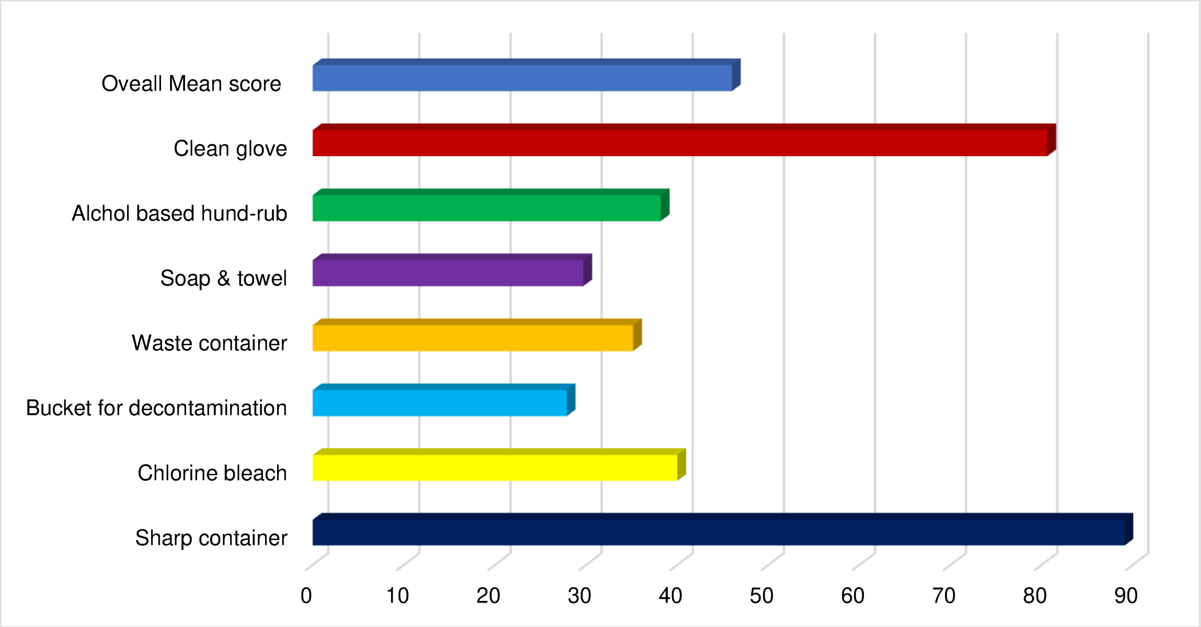

Supplementary figure 3: Percentages of mean score of standard precaution for infection prevention by tracer items.

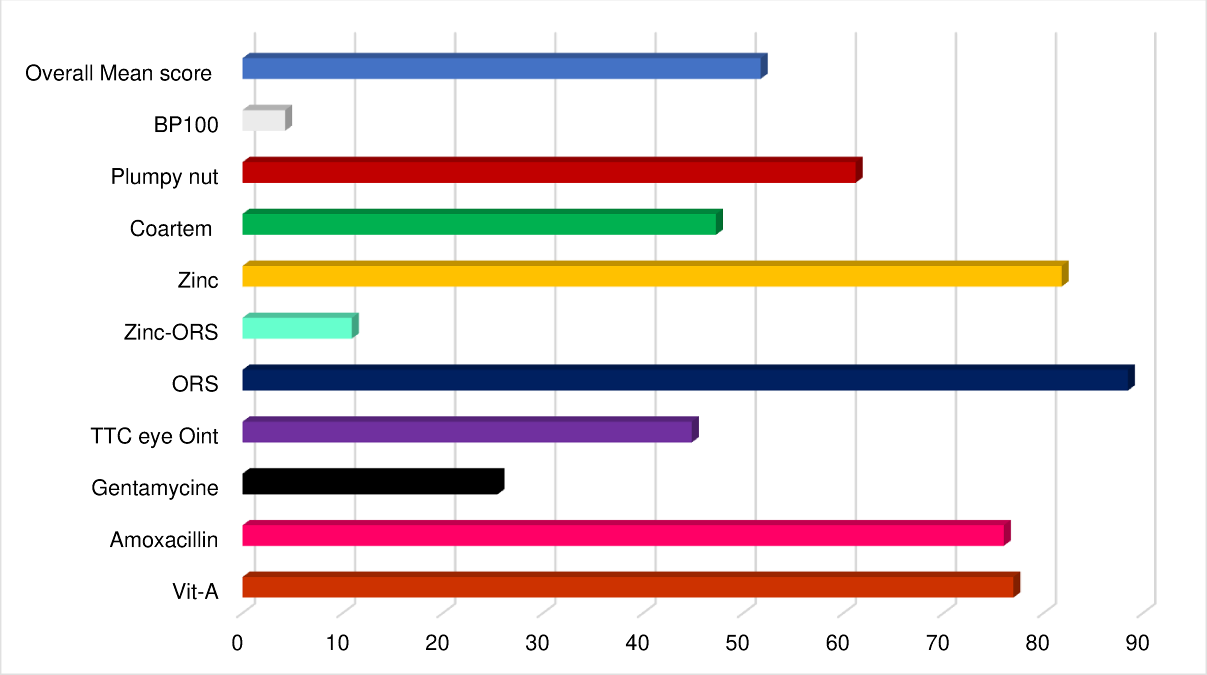

Supplementary figure 4: Percentages of mean score of essential medicines by tracer items.
