# Supplementary material for: Factors associated with the referral of children with severe illnesses at primary care level in Ethiopia: a cross-sectional study
Source: BMJ Open. 2021 Jun 9;11(6):e047640. doi: 10.1136/bmjopen-2020-047640 (PMC8194336; doi:10.1136/bmjopen-2020-047640)
Supplement: Supplementary data [file bmjopen-2020-047640supp002.pdf]

List of Supplementary tables

Supplementary table 1: Health post and child factors associated with referrals of 0 – 59 months sick young children in four regions of Ethiopia

Characteristics	Referral status of 0 – 59 days old sick young infants			Referral status of 2 – 59 months old children		
	Referred N=39 n (%)	Not referred N=190 n (%)	P-value	Referred N=78 n (%)	Not referred N=1045 n (%)	P-value
The health post						
Mean score of basic amenities						
0 to 33 percent	13 (24)	42 (76)	0.466	20 (13)	129 (87)	0.043
34 to 66 percent	9 (15)	50 (85)		19 (6)	292 (94)	
67 to 100 percent	17 (15)	98 (85)		39 (6)	624 (94)	
Mean score of basic equipment						
0 to 33 percent	2 (22)	7 (78)		3 (4)	73 (96)	
34 to 66 percent	10 (19)	43 (81)	0.825	12 (8)	147 (92)	0.512
67 to 100 percent	27 (16)	140 (84)		63 (7)	825 (93)	
Mean score of standard precaution						
0 to 33 percent	4 (9)	40 (91)		30 (8)	353 (92)	
34 to 66 percent	14 (19)	59 (81)	0.527	31 (8)	355 (92)	0.279
67 to 100 percent	21 (19)	91 (81)		17 (5)	337 (95)	
Rapid diagnostic test (RDT)						
0 to 33 percent	10 (17)	50 (83)		47 (9)	467 (91)	
34 to 66 percent	0	0	0.947	0	0	0.026
67 to 100 percent	29 (17)	140 (83)		31 (5)	578 (95)	
Mean score of essential medicines						
0 to 33 percent	1 (8)	11 (92)		17 (18)	77 (82)	
34 to 66 percent	32 (21)	113 (79)	0.026	43 (6)	697 (94)	0.001
67 to 100 percent	6 (8)	66 (92)		18 (6)	271 (94)	
Health post's overall service readiness index						
0 to 33 percent	0 (0)	1 (0)		7 (18)	31 (82)	
34 to 66 percent	22 (19)	93 (81)	0.737	43 (7)	575 (93)	0.193
67 to 100 percent	17 (15)	96 (85)		28 (6)	439 (94)	
Number of health extension worker per health post						

1	2 (5)	39 (95)	0.024	20 (9)	196 (81)	0.264
2 or more	37 (20)	151 (80)		58 (6)	849 (94)	
Distance between health post and the nearest referral health centre						
Distance to the next referral site[b]						
0 - 8 kms	16 (21)	61 (79)	0.554			
8.01 - 20 kms	10 (12)	71 (88)				
>20 kms	13 (19)	57 (81)				
0 - 8 kms				24 (7)	340 (93)	0.699
8.01 - 14 kms				25 (8)	301 (92)	
>14 kms				22 (6)	362 (94)	
The child						
Region						
Amhara	3 (25)	9 (75)	0.722	34 (7)	428 (93)	0.691
Oromia	10 (13)	65 (87)		26 (7)	342 (93)	
SNNPR[a]	9 (21)	33 (79)		11 (8)	128 (92)	
Tigray	17 (17)	83 (83)		7 (5)	147 (95)	
Age						
1st week	14 (24)	45 (76)	0.353			
2 – 4 weeks	16 (16)	82 (84)				
5 – 8 weeks	9 (13)	63 (87)				
2 – 11 months				26 (8)	294 (92)	0.249
12 – 23 months				28 (8)	325 (92)	
24 – 59 months				24 (5)	426 (95)	
Sex						
Boy	18 (15)	106 (85)	0.395	40 (7)	550 (93)	0.810
Girl	21 (20)	84 (80)		38 (7)	495 (93)	
Disease classification						
Non-severe illnesses not requiring referral	21 (12)	159 (88)	0.013	56 (5)	1023 (95)	0.001
Severe illnesses requiring referral	18 (37)	31 (63)		22 (50)	22 (50)	

[a] SNNPR - Southern Nations, Nationalities, and Peoples Region

[b] GIS based data of distance from health post to health centre were missing for 0-59 days sick young infant (1) and 2-59 months sick children (49).

Supplementary table 2: Individual health post readiness factors associated with referrals of 0-59 months sick children in four regions of Ethiopia

Characteristics		Referral status of 0 – 59 days sick young infants			Referral status of 2 – 59 months old children		
		Referred N=39 n (%)	Not referred N=190 n (%)	P-value	Referred N=78 n (%)	Referred N=1045 n (%)	P-value
Availability of basic amenities							
Water source							
	Not available	15 (22)	53 (78)	0.296	30 (8)	356 (92)	0.558
	Available	24 (15)	137 (85)		48 (7)	689 (93)	
Electric power							
	Not available	18 (17)	91 (83)	0.886	51 (8)	602 (92)	0.257
	Available	21 (18)	99 (82)		27 (6)	443 (94)	
Facility owned toilet							
	Not available	6 (18)	27 (82)	0.880	14 (7)	207 (93)	0.981
	Available	33 (17)	163 (83)		64 (7)	838 (93)	
Facility owned landline or mobile phone							
	Not available	35 (19)	153 (81)	0.133	51 (6)	828 (94)	0.038
	Available	4 (10)	37 (90)		27 (11)	217 (89)	
Availability of basic equipment							
Thermometer							
	Not available	8 (14)	49 (86)	0.594	12 (5)	228 (95)	0.207
	Available	31 (18)	141 (82)		66 (8)	817 (92)	
Child weight scale							
	Not available	5 (13)	34 (87)	0.526	20 (9)	214 (91)	0.388
	Available	34 (18)	156 (82)		58 (7)	831 (93)	
Stethoscope							
	Not available	18 (19)	79 (81)	0.709	35 (8)	399 (92)	0.336
	Available	21 (16)	111 (84)		43 (6)	646 (94)	
MUAC tape measure							
	Not available	1 (100)	0	0.040	0 (0)	14 (100)	0.701
	Available	38 (17)	190 (83)		78 (7)	1031 (93)	
Availability diagnostics							
Rapid diagnostic test (RDT)							
	Not available	10 (17)	50 (84)	0.947	47 (9)	467 (91)	0.026
	Available	29 (17)	140 (82)		31 (5)	578 (95)	

Availability of essential medicines							
Vitamin A							
	Not available	9 (27)	25 (73)	0.211	11 (5)	207 (95)	0.214
	Available	30 (15)	165 (85)		67 (7)	838 (93)	
Amoxicillin							
	Not available	6 (14)	36 (86)	0.745	28 (15)	159 (85)	0.001
	Available	33 (18)	154 (82)		50 (5)	886 (95)	
Gentamycin							
	Not available	20 (14)	127 (86)	0.201			
	Available	19 (23)	63 (77)				
Tetracycline eye ointment							
	Not available	26 (19)	110 (81)	0.457	46 (8)	567 (92)	0.554
	Available	13 (14)	80 (86)		32 (6)	478 (94)	
Oral rehydration solution (ORS)							
	Not available	2 (13)	13 (87)	0.627	10 (17)	48 (83)	0.048
	Available	37 (17)	177 (83)		68 (6)	997 (94)	
Zinc-ORS							
	Not available				69 (7)	947 (93)	0.608
	Available				9 (8)	98 (92)	
Zinc							
	Not available				16 (9)	152 (91)	0.331
	Available				62 (7)	893 (93)	
Coartem							
	Not available	18 (20)	71 (80)	0.494	47 (9)	507 (91)	0.087
	Available	21 (15)	119 (85)		31 (5)	535 (95)	
Plumpy Nut							
	Not available	8 (23)	27 (77)	0.404	30 (8)	372 (92)	0.688
	Available	31 (16)	163 (84)		48 (7)	673 (93)	
BP100							
	Not available	38 (17)	180 (83)	0.012	77 (7)	993 (93)	0.107
	Available	1 (9)	10 (91)		1 (2)	52 (98)	
