# Supplementary material for: Factors associated with the referral of children with severe illnesses at primary care level in Ethiopia: a cross-sectional study
Source: BMJ Open. 2021 Jun 9;11(6):e047640. doi: 10.1136/bmjopen-2020-047640 (PMC8194336; doi:10.1136/bmjopen-2020-047640)
Supplement: Author's manuscript [file bmjopen-2020-047640.draft_revisions.pdf]

BMJ Open

BMJ Open is committed to open peer review. As part of this commitment we make the peer review history of every article we publish publicly available.

When an article is published we post the peer reviewers' comments and the authors' responses online. We also post the versions of the paper that were used during peer review. These are the versions that the peer review comments apply to.

The versions of the paper that follow are the versions that were submitted during the peer review process. They are not the versions of record or the final published versions. They should not be cited or distributed as the published version of this manuscript.

BMJ Open is an open access journal and the full, final, typeset and author-corrected version of record of the manuscript is available on our site with no access controls, subscription charges or pay-per-view fees (http://bmjopen.bmj.com).

If you have any questions on BMJ Open's open peer review process please email info.bmjopen@bmj.com

BMJ Open

Factors associated with the referral of children with severe illnesses at primary care level in Ethiopia: a cross-sectional study.

Journal:	*BMJ Open*
Manuscript ID	bmjopen-2020-047640
Article Type:	Original research
Date Submitted by the Author:	05-Dec-2020
Complete List of Authors:	Beyene, Habtamu; Hawassa University, College of Medicine and Health Sciences; Southern Nations Nationalities and Peoples' Region, Regional Health Bureau<br>Kassa, Dejene; Hawassa University, College of Medicine and Health Sciences, School of Public Health<br>Tadele, Henok; Addis Ababa University, College of Health Sciences, Department of Paediatrics and Child Health<br>Persson, Lars; London School of Hygiene and Tropical Medicine Faculty of Infectious and Tropical Diseases; Ethiopian Public Health Institute<br>Defar, Atkure ; Ethiopian Public Health Institute, Health System and Reproductive Health Research Directorate ; University of Gondar, Epidemiology and Biostatistics, Institute of Public Health, College of Medicine and Health Sciences<br>Berhanu, Della; London School of Hygiene and Tropical Medicine Faculty of Infectious and Tropical Diseases; Ethiopian Public Health Institute
Keywords:	Organisation of health services < HEALTH SERVICES ADMINISTRATION & MANAGEMENT, Quality in health care < HEALTH SERVICES ADMINISTRATION & MANAGEMENT, Community child health < PAEDIATRICS, EPIDEMIOLOGY

SCHOLARONE™
Manuscripts

I, the Submitting Author has the right to grant and does grant on behalf of all authors of the Work (as defined in the below author licence), an exclusive licence and/or a non-exclusive licence for contributions from authors who are: i) UK Crown employees; ii) where BMJ has agreed a CC-BY licence shall apply, and/or iii) in accordance with the terms applicable for US Federal Government officers or employees acting as part of their official duties; on a worldwide, perpetual, irrevocable, royalty-free basis to BMJ Publishing Group Ltd ("BMJ") its licensees and where the relevant Journal is co-owned by BMJ to the co-owners of the Journal, to publish the Work in this journal and any other BMJ products and to exploit all rights, as set out in our licence.

The Submitting Author accepts and understands that any supply made under these terms is made by BMJ to the Submitting Author unless you are acting as an employee on behalf of your employer or a postgraduate student of an affiliated institution which is paying any applicable article publishing charge ("APC") for Open Access articles. Where the Submitting Author wishes to make the Work available on an Open Access basis (and intends to pay the relevant APC), the terms of reuse of such Open Access shall be governed by a Creative Commons licence – details of these licences and which Creative Commons licence will apply to this Work are set out in our licence referred to above.

Other than as permitted in any relevant BMJ Author's Self Archiving Policies, I confirm this Work has not been accepted for publication elsewhere, is not being considered for publication elsewhere and does not duplicate material already published. I confirm all authors consent to publication of this Work and authorise the granting of this licence.

For peer review only

Factors associated with the referral of children with severe illnesses at primary care level in Ethiopia: a cross-sectional study.

Short run title: Referral of sick children in Ethiopia

Habtamu Beyene[1, 2], Dejene Hailu[2], Henok Tadele[3], Lars Åke Persson[4, 5], Atkure Defar[5, 6], Della Berhanu[4, 5]

Corresponding author:

Habtamu Beyene; MSc, BSc

College of Medicine and Health Sciences, School of Public Health

Hawassa University,

Hawassa, Ethiopia

Tel: +251-911-003346

Email: habtamu3346@yahoo.com

Co-authors:

Dr. Dejene Hailu Kassa; PhD, Assoc. Prof. of Public health, dejenkassa@yahoo.com

Dr. Henok Tadele; MD, Assoc. Prof. of Pediatrics & Child Health; henny_2007@yahoo.com

Prof. Lars Åke Persson; MD, PhD, lars.persson@lshtm.ac.uk

Atkure Defar; MPH-E, MBA, atkuredefar@gmail.com

Dr. Della Berhanu; PhD, della.berhanu@lshtm.ac.uk

For peer review only

ABSTRACT

Context and objective: Ethiopia's primary care has a weak referral system for sick children. We aimed to identify health post and child factors associated with referrals of sick children 0-59 months of age.

Design: A cross-sectional facility-based survey.

Setting: This study included 165 health posts in 52 districts in four Ethiopian regions collected from December 2018 to February 2019. Data included interviews with health extension workers, assessment of health post preparedness, recording of GPS-coordinates of the health post and the referral health centre, and reviewing registers of sick children treated during the last three months at the health posts. We analysed the association between the sick child's characteristics, the health post preparedness, and distance to the health centre with referral of sick children by multivariable logistic regressions.

Outcome measure: Referral to the nearest health centre of sick young infants aged 0-59 days and sick children 2-59 months.

Results: The health extension workers referred 39/229 (17%) of the sick young infants and 78/1123 (7%) of the older children to the next level of care. Only 18 (37%) sick young infants and 22 (50%) 2-59 months children that deserved urgent referral according to guidelines were referred. The leading causes of referral were possible serious bacterial infection and pneumonia. Those being classified as a severe disease were referred more frequently. The availability of basic amenities (AOR = 0.38, 95% CI: 0.15 – 0.96), amoxicillin (AOR = 0.41, 95% CI: 0.19 - 0.88) and rapid diagnostic test (AOR = 0.18, 95% CI: 0.07 - 0.46) were associated with less referral in the older age group.

Conclusion: Few children with severe illness were referred from health posts to health centres. Improving the health posts' medicine and diagnostic supplies may enhance adherence to referral guidelines and ultimately reduce child mortality.

Key words: health extension workers, referral, severe illnesses, sick child, referral factors.

For peer review only

Strengths and limitations of this study

- Based on secondary analysis of register data from 165 health posts in 52 districts of the four most populous regions of Ethiopia, this study reports primary care referral patterns and associated factors in rural areas.
- This study used objectively measured data based on the WHO health service availability and readiness assessment tool and information obtained from the surveyed health posts compared with the national guidelines on referral.
- Using a cross-sectional study design, we cannot prove any causal relationship between the health post readiness, child factors, and referral of sick children observed in this study.
- The study findings do not give a complete picture of the country's referral system as we limited the analysis to the primary healthcare level, and we did not collect data from parents of the referred children.

For peer review only

INTRODUCTION

Globally, nearly 5.3 million children below the age of 5 years died in 2018. Of these deaths, 47% occurred during the first month of life. More than half of this mortality occurred in Sub-Saharan Africa. Infectious diseases remained the leading causes of death (1).

Ethiopia achieved a significant reduction in child mortality during the Millennium Development Goals era from 1990 to 2015 (2). However, under-five and neonatal mortality rates remain high with 55 and 30 deaths per 1,000 live births, respectively (3). Lower respiratory tract infection, diarrhoeal diseases, and neonatal causes (mainly preterm birth complications, asphyxia, and neonatal sepsis), were the leading causes of death (4).

The integrated case management of childhood illnesses and the community based newborn care programmes are, if delivered with quality, cost-effective in reducing under-five deaths (5,6). Referral is included as a critical element in these programmes (7). In Ethiopia, the integrated community case management programme was initiated in 2010 (8). This programme trained health extension workers to assess, classify, and treat sick children 2-59 months of age with pneumonia, diarrhoea, malnutrition, and other common illnesses and refer severe cases to health centres according to guidelines (9). In 2013, the Community-Based Newborn Care programme was initiated. Health extension workers were trained to provide pre-referral antibiotic treatment for young infants (0-59 days) with possible severe bacterial infection and refer those to a higher-level facility. If a referral was not possible, the health workers were instructed to provide a seven-day antibiotic treatment. These interventions have been implemented in Ethiopia within the existing Health Extension Program (6,10).

A referral is defined as "a process in which a health worker at one level of the health system, having insufficient resources (drugs, equipment, skills) to manage a clinical condition, seeks the assistance of a better or differently resourced facility at the same or higher level to assist in or take over the management of the client's case" (11). A well-functioning referral system is an essential part of the health system and an indicator of the quality of care provided at health facilities (7). The referral pattern may reflect the healthcare

For peer review only

workers' ability to identify signs of severe illness at an early stage (12). For effective referral care, a pre-requisite is also that the higher-level facility is ready to receive and treat these children appropriately (7).

In a previous study, we showed that the Ethiopian health extension workers referred only a fraction of the severely sick young infants and under-five children that deserved urgent referral to health centres. Generally, referral logistics were weak (13). Other studies in low-income countries have shown that the distance to the referral site, the severity of the child's illnesses (14), and the availability of referral logistics (15,16) may influence severely sick children's referral.

This study aimed to identify factors associated with the referral of severely ill children aged 0-59 months in Ethiopia's primary health care system. Specifically, we assessed the referral of sick children 0-59 months from health post to the next higher level of health care in relation to guidelines. We analysed factors associated with referral care, including child characteristics, health post preparedness, and distance to the next-level health facility.

METHODS

Study design and settings

This study was based on data from the evaluation of Optimizing the Health Extension Programme intervention. This intervention was a collaborative effort between the Government of Ethiopia and implementing partners to increase the use and quality of community-based newborn care and integrated community case management of childhood illnesses in four Ethiopian regions (17).

We did a secondary analysis of data from the endline cross-sectional survey conducted in 26 districts, where the Optimizing the Health Extension Programme intervention was implemented, and in 26 comparison districts without the intervention. These districts were found in four major Ethiopian regions, namely Amhara, Oromia, Southern Nations, Nationalities and Peoples (SNNP), and Tigray. The survey was conducted from December 2018 to February 2019, after the implementation of the intervention.

For peer review only

In Ethiopia, primary health care services are provided within the district health system, comprised of five primary health care units and a district hospital serving around 100,000 people. Each primary health care unit consists of five health posts and their referral health centre. The health post is typically staffed with two health extension workers and serves a population of 5000 people (18).

The referral care of sick children 0-59 months of age from health posts to the next level was assessed in relation to the treatment guidelines for 0-59 days old sick young infants (community-based newborn care), and 2 – 59 months old children (integrated community case management).

According to these guidelines, babies 0-59 days who are very preterm (< 32 weeks), very low birth weight (< 1500gm), preterm (32-37 weeks), low birth weight (1500-2500gm), have a possible severe bacterial infection, severe jaundice, severe dehydration, severe persistent diarrhoea, or dysentery require an urgent referral. Similarly, children 2-59 months with a possible severe bacterial infection, severe dehydration, severe pneumonia, possible severe bacterial infections, severe persistent diarrhoea, very severe febrile disease, severe complicated measles, mastoiditis, severe anaemia, and complicated severe acute malnutrition require an urgent referral (19).

Study population and sample

The primary study had household and health facility modules to evaluate the Optimizing the Health Extension Programme intervention (20). We used the 2007 Ethiopian census to select 200 enumeration areas proportional to the population size of the 52 districts included in the study. In the main study, households were randomly selected to represent the enumeration areas. The health posts serving the selected households were included in this study. All records of 0-59 days old sick young infants, who sought care in three months prior to the survey, and records of the most recent ten 2-59 months sick children, who also sought care, were reviewed from treatment registers at the health post. Children without any illness who visited the health posts for postnatal care, vaccination, and growth monitoring were excluded from the analysis.

The sample size for the main study was based on the requirement that the baseline and end-line surveys should measure changes of a fixed number of percentage points of selected indicators for care-seeking and treatment for a sick child, based on household interviews.

Data collection

The questionnaires used in this study were adapted from existing large-scale survey tools, such as the Demographic and Health Surveys and the Service Provision Assessment survey (21). Questionnaires were translated into three local languages (Amharic, Afan Oromo and Tigrigna) and pre-tested. All data were collected using tablet computers.

Out of the eligible 200 clusters, 19 clusters were excluded from the study due to civil unrest. Trained health professionals and supervisors collected the data.

We collected data on the number of health extension workers at the health posts, availability of amenities, equipment, supplies, diagnostics, and medicines. Geographic Positioning System (GIS) coordinates were assessed at health posts and referral health centres. We reviewed registers of 0-59 days and 2-59 months old children and collected background data on their symptoms, disease classifications, treatment, and referral status.

Patient and Public Involvement: Not applicable (patients were not involved in this study).

Data analysis

A descriptive analysis was performed, including frequencies, percentages, and means. We characterized the health posts, the distance from health posts to the referral health centres, the proportion of health posts with service readiness tracer items and referral logistics for childhood referral care, and the referral of sick children of 0-59 months from health posts to higher levels in relation to the treatment guidelines.

We used health posts and their respective referral health centres' GPS reading to calculate the two's straight-line distance. The health post's facility readiness was assessed using the World Health Organization service availability and readiness assessment (SARA) reference manual (22). The general health facility

For peer review only

service readiness has five domains, which include different tracer indicators, items, and services. The first is basic amenities, including four items: communication, access to toilet facilities for clients, water source, and power supply. The second is basic equipment with four items: infant or child scale, thermometer, functional stethoscope, and mid-upper arm circumference tape measure. The third is standard precautions for infection prevention with seven items: sharps container, chlorine bleach, bucket for decontamination solution, contaminated waste container, soap, and a towel or hand rub, alcohol-based hand rub, and clean gloves. The fourth is the basic diagnostic capacity with a rapid diagnostic test for malaria. The last is essential medicines with ten items: vitamin A, tetracycline eye ointment, gentamycin, amoxicillin, oral rehydration solution, zinc-oral rehydration solution combined, zinc, and the ready-to-use- therapeutic foods (plumpy nut and BP100). Finally, the combined facility service readiness was assessed using the general service readiness index by calculating five domains' mean score.

The attributes of the referred sick children, their symptoms, disease classification, and treatment were analysed based on the existing treatment guidelines (19). Accordingly, the proportion of sick children with severe illnesses that require an urgent referral, non-severe illnesses that do not require urgent referral, the numbers referred and causes of their referrals were analysed. The referral status was linked with the facility readiness, and other health post factors, including the distance to the referral facility.

Referral of 0-59 days old sick young infants, and 2-59 months old children were the outcomes separately dichotomized as "yes" (referred) or "no" (not referred). Chi-square tests were performed to assess whether the child's characteristics, health post preparedness, and the distance to the next level health facility were associated with a sick child's referral. Relevant factors with statistically significant association to the referral of sick children in the bivariate analysis were entered into a multivariable logistic regression analysis to identify referral predictors. Adjustments were made for clustering and whether the cluster was an Optimizing the Health Extension Program intervention or comparison area. Odds ratios (ORs) with 95% confidence intervals (CIs) were calculated to measure the association between the predictor and outcome variables.

For peer review only

Additionally, adherence to referral guidelines for sick young infants and older children was dichotomised as "yes" (managed according to referral guideline) or "no" (not managed according to referral guideline). Factors that had statistically significant association with referrals were then assessed in bivariate and multivariable logistic regression analyses to identify predictors of adherence to correct referral practices.

The Census and Survey Processing System (CSPro) software (United States Census Bureau, Washington DC, USA), was used to enter and edit survey data. Statistical analysis and tests were done using SPSS version 25 (SPSS, IBM Corp, New York, USA).

RESULTS

We extracted data from treatment registers at 165 health posts serving 181 enumeration areas. Information on the assessment, treatment and referral of 229 sick young infants of 0 – 59 days and 1123 sick children aged 2-59 months was collected from the registers. Out of the 165 health posts, 48 had registered to manage one or more 0-59 days old sick young infants, and 147 had registered one or more 2-59 months old sick children in the three months preceding the survey.

Characteristics of health posts

Nearly 80% of health posts were staffed with two or more health extension workers, while the rest had only one. Almost all health posts had sick child registers, and 42% had referred at least one sick child to a health centre in the quarter before the survey. A cell phone signal was available in most of the surveyed health posts. Still, most health workers had neither used mobile phones nor other communication means for their most recent referral of a sick child (Table 1). Half of the surveyed health posts were found within 10 kilometres from the next level referral health centre (Figure 1). There were missing distance data for seven health posts.

For peer review only

Health post readiness status

The readiness of health posts to provide health services was assessed for five general service readiness domains. The average readiness score was 61% for basic amenities, 80% for basic equipment, 49% for standard precaution for infection prevention, 50% for basic diagnostic capacity, and 52% for essential medicines (Figure 2). A significant proportion of the health posts lacked readiness to provide the required service for sick children, shown by an overall service readiness index of 57%. Moreover, nearly half of the health posts did not have the required essential medicines including those needed to treat possible severe bacterial infection of 0-59 days sick young infants. The items included in these scores are shown in supplementary figure 1 – 4.

Referral of sick children

During the last three months before the survey, 39 (17%) of the 229 examined 0 – 59 days old sick young infants were referred from the health posts to the nearest health centres. Similarly, 78 (7%) of the 1123 sick children aged 2 – 59 months old had been referred. Possible serious bacterial infection and pneumonia were the leading causes of referral among 0 – 59 days old and 2 – 59 months old children, respectively (Table 2).

Referral in different groups

Health posts in the lowest and highest tertiles of essential medicines referred fewer sick young infants than the middle tertile (p=0.026). A higher proportion of sick young infants was referred from health posts served by two or more health extension workers than those served by only one (p=0.024). Among the sick 0-59 days old sick young infants with a severe disease classification that, according to guidelines, require a referral, 37% were referred to the next level, while among those with an illness that does not require a referral, 12% of were referred (p=0.013) (Table 3).

Health posts within the lowest tertile score for basic amenities (p=0.043), diagnostics (p=0.026), and essential medicines (p=0.001) referred more sick older children than the other tertiles (Table 3). Cross-tabulation of the individual components across the four facility readiness domains is shown in supplementary

table 1. Further, health posts that had communication facility (p=0.038), and amoxicillin (p=0.001) reported fewer referrals of older children than those without (Supplementary table 1). Among the sick 2 – 59 months old children with a severe illness classification that deserves referral, half were referred to the next level, while among those with an illness that does not require a referral 5% were referred (p=0.001).

There was no difference in referral between age groups or boys and girls among young infants as well as older children.

Similarly, the distance between health post and the referral health centre was neither associated with the referral of the sick young infants nor the older sick children (Table 3).

Factors associated with referral

Sick young infants aged 0-59 days with severe illnesses were seven times more likely to be referred to the next referral level, as compared to those with non-severe illnesses (95% CI: 1.50 - 30.11) (Table 4). Sick young infants in the later weeks of life were less likely to be referred than those in the first week (AOR = 0.25, 95% CI: 0.10 - 0.64). Neither the sex of the sick child nor the distance from health post to referral health centre were associated with referral (Table 4).

Also, sick children aged 2-59 months with severe illness were more likely to be referred than those with non-severe illness (AOR = 21.27, 95% CI: 7.51 - 60.23) (Table 5). Health posts that had a better score for basic amenities were less likely to refer 2-59 months sick children to the next level than those without (AOR= 0.38, 95% CI: 0.15 – 0.96). Health posts with amoxicillin (AOR = 0.41, 95% CI: 0.19 - 0.88) and rapid diagnostic tests (AOR = 0.18, 95% CI: 0.07 – 0.46) were less likely to refer 2-59 months old children than those without these medicines and diagnostic kit.

The number of health extension workers, age and sex of the child and distance from health post to the referral health centre were not associated with referrals of 2-59 months old children.

For peer review only

Management according to guideline

The adherence to referral guidelines of sick young infants and older children is shown in supplementary table 2. Among the sick 229 young infants, 21 children were referred when a referral was not advised, and 31 children were treated at the health post when a referral was recommended. The remaining 177 (77%) were managed according to referral guidelines. Of the factors associated with a referral, essential medicines were also associated with adhering to referral guidelines for young infants. Those in the higher tertile adhered more to referral guidelines (AOR= 13, 95% CI: 1.5-112.6). Among the 1123 sick older children, 56 were referred when a referral was not advised, and 22 children were treated at the health post when a referral was required. The remaining 945 (93%) were managed according to referral guidelines. Facilities with amoxicillin and diagnostics were more likely to adhere to referral guidelines (amoxicillin AOR = 2.8, 95% CI: 1.3-5.5 and diagnostics AOR = 4.7, 95% CI: 1.8-11.9). Data for this analysis are shown in supplementary tables 3 and 4.

DISCUSSION

Our study from four major Ethiopian regions showed that health extension workers referred only around one-third of the sick young infants 0-59 days old and half of the 2-59 months old children that should be referred, according to guidelines. The leading diagnoses at referral were possible severe bacterial infections in young infants and pneumonia in older children. As expected, conditions classified as severe were referred more frequently than non-severe cases. The tendency to refer was associated with some characteristics. For 0-59 days old infants, young neonates were referred more often than a bit older young infants. Health posts with basic amenities, amoxicillin, and rapid diagnostic tests were less inclined to refer older sick children. Within the 2-59 months group, age was not associated with referral. There was no sex difference in referral. Further, the straight-line distance from the health post to the health centre was not associated with referrals.

Due to the cross-sectional study design, we cannot prove any causal relationship between the health post and child factors that were associated with a referral of sick children. Further, we have analysed referral from the first primary care level, and are therefore only displaying part of the country's referral system. We used

For peer review only

objectively measured data on the health posts, based on the WHO health service availability and readiness assessment tool, and information obtained from the surveyed health posts were compared with national guidelines on referral. The study was performed in four of the largest regions in Ethiopia accounting for 80% of the population, where the enumeration areas were selected to represent the chosen districts of these regions. Though generalization of the study findings to the regions might be difficult, it is expected that the selected districts did not differ in health systems characteristics.

Overall, we found that insufficient proportions of young infants and older children, who required referral, were referred from health post to the health centres. The World Health Organization recommends that 0-59 days old sick young infants with possible severe bacterial infections are managed at the primary care level when referral to a higher level is impossible (23). In this study, only one in three sick young infants with signs of possible severe bacterial infection was referred from health posts. Similarly, only about half of the eligible sick older children that required referral were referred to the next level. This finding was consistent with the previous Ethiopian and Malawian studies (9, 24).

The low referrals seen might be attributed to health providers' fear that caregivers would protest a referral (25), and the actual refusal of caregivers to comply with the referral (26). These factors might lead the health extension workers to manage the child at the health post (27). Health care providers might also undermine the overall impact of treatment guidelines on childhood survival (25, 28). They may perceive that there is little added value in referral and even feel a loss of power and prestige when referring (29). They might also lack the knowledge and be reluctant to adhere to the guidelines (29). This low level of referral of severely sick children is concerning. It could delay access to appropriate care in the higher-level referral facilities, thus increasing the risk of death (30), and hinder the efforts to reach the sustainable development goals on neonatal and under-five mortality (31).

In this study, the severity of a child's illness was associated with their referral in both age groups (32). This relation indicates that severely ill children were more frequently managed according to the referral guidelines. There is a need to strengthen health extension workers' ability to identify severely sick children

For peer review only

requiring urgent referral (33). Neonates were primarily referred in their first week of life, highlighting the need for urgent referrals of severely ill newborns to prevent death in neonatal sepsis and other critical conditions (34). There was no difference in referrals between boys and girls. Also, we found that distance from health posts to health centre was not associated with referrals, perhaps since nearly three-quarters of the health posts were located within 15 kilometres from their referral health centre.

Nearly four out of ten health posts did not have the overall readiness to provide the required childcare services, based on the World Health Organization's service availability and readiness assessment (SARA) reference manual (22). Moreover, the necessary essential medicines were lacking in nearly half of the surveyed health posts. This scenario could indicate that many care-seeking sick children were not getting the care they required, at least not at the health post (35). The lack of essential medicines might be a reason for some referrals of children with non-severe diseases, which otherwise may be treated at the first-level health facility (32,36). This condition might also impose caregiver for unnecessary social and economic costs incurred by referrals of children with non-severe illnesses (36,37).

Indeed, our data showed that health posts that had medicine and diagnostics were likely to adhere to referral guidelines. Although not statistically significant in the multivariable analysis, health posts with two or more health extension works were also more likely to refer as well as adhere to referral guidelines for young infants. A study in Niger also found adequate staffing to be linked to adherence to referral guidelines (29).

Conclusion

We have shown that health extension workers referred few children with severe illnesses while a referral to the next-level health centre was recommended by guidelines. A significant proportion of the health posts were not ready to provide the necessary care, including referral. The severity of the child's illness, the availability of essential medicines and rapid diagnostic test were among the predictors of referrals of sick children from the health posts. Among other actions to enhance the quality of care provided at the health posts, improving

For peer review only

medicine and diagnostic supplies may strengthen adherence to referral guidelines and ultimately prevent child deaths in Ethiopia.

Author affiliations

[1] Southern Nations, Nationalities & Peoples Regional Health Bureau, Department of maternal, newborn, and child health, and nutrition, Hawassa, Ethiopia

[2] Hawassa University, College of Medicine and Health Sciences, School of Public Health, Hawassa, Ethiopia

[3] Addis Ababa University, College of Health Sciences, Department of Paediatrics and Child Health, Addis Ababa, Ethiopia

[4] London School of Hygiene and Tropical Medicine, London, UK

[5] Ethiopian Public Health Institute, Addis Ababa 5654, Ethiopia

[6] Department of Epidemiology and Biostatistics, Institute of Public Health, College of Medicine and Health Sciences, University of Gondar, Gondar, Ethiopia

Acknowledgments

We would like to extend our sincere gratitude to SNNP Regional Health Bureau and Hawassa University, College of Medicine and Health Sciences for providing support to conduct this research project. We would also like to pass our deepest appreciation to the different levels of the health systems and health providers involved in this study.

Authors' contributions

HB contributed to the conceptualization of the study, design, development of the survey instrument, training and supervision of data collectors, statistical analysis, and wrote the original draft and final versions of the manuscript. DK and HT contributed to the conceptualization of the study design, reviewed and contributed to the different versions of the manuscript. DB and LÅP contributed to the conceptualization of the study, design, development of the survey instrument, data compilation and statistical analysis, and reviewed the different

For peer review only

versions of the paper. AD contributed to the management of distance from health post to health centre data. All authors read and approved the final version of the manuscript.

Funding: The study was supported by a grant from Bill and Melinda Gates Foundation to The London School of Hygiene and Tropical Medicine (grant OPP1132551). The funder had no role in data collection, analysis, or interpretation of results.

Disclaimer: Not required.

Competing interests: The authors declared no competing interests exist.

Patient consent for publication: Not required.

Ethics approval

Ethical approvals were secured from the ethical review boards of Hawassa University (protocol number IRB/199/10; June 2018), the Ethiopian Public Health Institute (protocol number SERO-012-8-2016; Version 001, August 2016) and the London School of Hygiene & Tropical Medicine (protocol number 11235; June 2016).

Data availability statement: Data from this study are co-owned by the participating institutions and stored in a depository at the Ethiopian Public Health Institute (EPHI). The use of these data is guided by a data sharing agreement. Data can be accessed from the secretary of the data sharing committee of EPHI-LSHTM collaborative projects. Contact information: Martha Zeweldemariam, E-mail: martha.zeweldemariam@lshtm.ac.uk

ORCID IDs

Habtamu Beyene https://orcid.org/0000-0003-0923-5191

Della Berhanu https://orcid.org/0000-0002-4984-893X

Atkure Defar http:// orcid.org/ 0000-0001-9435-2135

Lars Åke Persson https://orcid.org/0000-0003-0710-7954

For peer review only

References

1. United Nations Inter-agency Group for Child Mortality Estimation (UNIGME), 'Levels & Trends in Child Mortality: Report 2019, Estimates developed by the United Nations Inter-agency Group for Child Mortality Estimation', United Nations Children's Fund, New York, 2019.
2. Ruducha J, Mann C, Singh NS, et al. Articles How Ethiopia achieved Millennium Development Goal 4 through multisectoral interventions : a Countdown to 2015 case study. *Lancet Glob Heal.* 2015; 5 (11):e1142–51.
3. Ethiopian Public Health Institute (EPHI) [Ethiopia] and ICF. 2019. Ethiopia Mini Demographic and Health Survey 2019: Key Indicators. Rockville, Maryland, USA: EPHI and ICF. Available from: https://www.dhsprogram.com/pubs/pdf/PR120/PR120.pdf. Accessed on August 24, 2020.
4. Deribew A; Tessema GA; Deribe K; Melaku YA; Lakew Y; Amare AT; Abera SF; Mohammed M; Hiruye A; Teklay E; Misganaw A; Kassebaum N. Trends, causes, and risk factors of mortality among children under 5 in Ethiopia, 1990-2013: findings from the Global Burden of Disease Study 2013. *Popul Health Metr.* 2016; 14:42.
5. Mugeni, C., Levine, A. C., Munyaneza, R. M., Mulindahabi, E., Hannah, C., Habimana, J. P. …Ngabo, F. Nationwide implementation of integrated community case management of childhood illness in Rwanda. *Glob Heal Sci Pract.* 2014; *2*: 328–341.
6. Mathewos B, Owen H, Sitrin D, *et al*. Community-Based interventions for newborns in Ethiopia (COMBINE): cost-effectiveness analysis. *Health Policy Plan* 2017; 32:i21–32.
7. World Health Organisation. Standards for improving the quality of care for children and young adolescents in health facilities. Geneva: World Health Organization, 2018. CC BY-NC-SA 3.0 IGO.
8. Miller NP, Amouzou A, Tafesse M, Hazel E, Legesse H, Degefie T, et al. Integrated Community Case Management of Childhood Illness in Ethiopia : Implementation Strength and Quality of Care. *Am. J. Trop. Med. Hyg.* 2014; 91:424–34.

For peer review only

9. Amouzou A, Hazel E, Shaw B, Miller NP, Tafesse M, Mekonnen Y, et al. Effects of the integrated Community Case Management of Childhood Illness Strategy on Child Mortality in Ethiopia : A Cluster Randomized Trial. *Am J Trop Med Hyg.* 2016; 94:596–604.
10. Marsh DR, Hamer DH, Pagnoni F, Peterson S. Introduction to a Special Supplement : Evidence for the Implementation, Effects, and Impact of the Integrated Community Case Management Strategy to Treat Childhood Infection. *Am J Trop Med Hyg.* 2012; 87:2–5.
11. World Health Organization. Referral Systems - a summary of key processes to guide health services managers. Geneva: World Health Organization, 2013. Available at: https://www.who.int/management/facility/referral/en/. Accessed on August 10, 2020.
12. Price J, Willcox M, Kabudula CW, Herbst K, Hinton L, Kahn K, et al. Care pathways during a child's final illness in rural South Africa: Findings from a social autopsy study. *PLoS ONE* 2019; 14: e0224284.
13. Beyene H, Hailu D, Tadele H, Persson LÅ, Berhanu D. Insufficient referral practices of sick children in Ethiopia based on a cross-sectional survey. *Acta Paediatr.* 2020*; 00:1–8.*
14. Abrahim O, Linnander E, Mohammed H, Fetene N, Bradley E. A Patient-Centered Understanding of the Referral System in Ethiopian Primary Health Care Units. *PLoS One*. 2015; 10:1–10.
15. Eskandari M, Abbaszadeh A, Borhani F. Barriers of Referral System to Health Care Provision in Rural Societies in Iran. *J Caring Sci.* 2013; 2:229–36.
16. Akbari A, Mayhew A, Al-alawi MA, Grimshaw J, Winkens R, Glidewell E, et al. Interventions to improve outpatient referrals from primary care to secondary care. *Cochrane Database Syst Rev.* 2014; 4: 1-50.
17. Berhanu D, Okwaraji YB, Belayneh AB, *et al*. Protocol for the evaluation of a complex intervention aiming at increased utilisation of primary child health services in Ethiopia: a before and after study in intervention and comparison areas. *BMC Health Serv Res.* 2020; 20:339.
18. Global financing facility. Ethiopia Health Sector Transformation Plan (2015/16-2019/20). https://www.globalfinancingfacility.org/ethiopia-health-sector-transformation-plan-201516-201920. Accessed on November 22, 2020.

For peer review only

19. FMOH. Federal Ministry of Health, Ethiopia. *Integrated Management of Newborn and Childhood Illness, Part 1*. Blended Learning Module for the Health Extension Programme. 2011. Retrieved from https://www.open.edu/openlearncreate/-pluginfile.php/71990/-mod_resource/content/2/IMNCI-_Part_1_Final_Print-ready_March_2011_.pdf. Accessed on November 15, 2020.

20. Berhanu D, Okwaraji YB, Defar A, et al. Does a complex intervention targeting communities, health facilities and district health managers increase the utilisation of community- based child health services? A before and after study in intervention and comparison areas of Ethiopia. *BMJ Open* 2020; 10:e040868.

21. Central Statistical Agency (CSA) [Ethiopia] and ICF (2016). Ethiopia: Demographic and Health Survey 2016 [Internet]. 2016. Available from: https://www.dhsprogram.com/publications/-publication-FR328-DHS-Final-Reports.cfm. Accessed on November 22, 2020.

22. World Health Organization. Service availability and readiness assessment (SARA). An annual monitoring system for service delivery. Reference manual. Geneva; 2015. Available from http://www.who.int/healthinfo/systems/sara_indicators_questionnaire/en/. Accessed 20 October, 2020.

23. World Health Organization. Guideline: managing possible serious bacterial infection in young infants when referral is not feasible. World Health Organization. Geneva; 2015. https://apps.who.int/iris/-handle/10665/181426. Accessed 20 October, 2020.

24. Amouzou A, Kanyuka M, Hazel E, Heidkamp R, Marsh A, Mleme T, et al. Independent Evaluation of the integrated Community Case Management of Childhood Illness Strategy in Malawi Using a National Evaluation Platform Design. *Am J Trop Med Hyg.* 2016; 94:574–83.

25. Chinbuah MA, Abbey M, Kager PA, Gyapong M, Nonvignon J, Ashitey P, Akpakli J, Appiatse SA, Kubi D, Gyapong JO. Assessment of the adherence of community health workers to dosing and referral guidelines for the management of fever in children under 5 years: a study in Dangme West District, Ghana. *Int Health*. 2013; 5:148-56.

For peer review only

26. Hailegebriel TD, Mulligan B, Cousens S, Mathewos B, Wall S, Bekele A, et al. Effect on Neonatal Mortality of Newborn Infection Management at Health Posts When Referral Is Not Possible : *Glob Heal Sci Pract.* 2017; 5:202–16.
27. Wammanda RD, Adamu SA, Joshua HD, Nisar YB, Qazi SA, Aboubaker S, et al. Implementation of the WHO guideline on treatment of young infants with signs of possible serious bacterial infection when hospital referral is not feasible in rural Zaria, Nigeria: Challenges and solutions. *PLoS ONE*. 2020; 15: e0228718.
28. Wanduru P, Tetui M, Tuhebwe D, Ediau M, Okuga M, Nalwadda C, Ekirapa-Kiracho E, Waiswa P, Rutebemberwa E. The performance of community health workers in the management of multiple childhood infectious diseases in Lira, northern Uganda - a mixed methods cross-sectional study. *Glob Health Action.* 2016; 9:33194.
29. Bossyns P, Lerberghe W Van. Human Resources for Health The weakest link : competence and prestige as constraints to referral by isolated nurses in rural Niger. *BioMed Cent.* 2004; 8:1–8.
30. Nabongo P, Verver S, Nangobi E, Mutunzi R, Wajja A, Mayanja-kizza H, et al. Two year mortality and associated factors in a cohort of children from rural Uganda. *BMC Public Health.* 2014; 14:1–9.
31. Golding, N., Burstein, R., Longbottom, J., Browne, A. J., Fullman, N., Osgood-zimmerman, A., … Foundation, M. G. Mapping under-5 and neonatal mortality in Africa , 2000 – 15 : a baseline analysis for the Sustainable Development Goals. *Lancet*. 2015; *390*, 2171–2182.
32. Musa EO, Ejembi CL. Reasons and outcome of paediatric referrals from first- level health facilities in Sabongari , Zaria , Northwestern. *J Community Med Prim Heal Care.* 2004; 16:10–5.
33. Newbrander W, Ickx P, Werner R, Mujadidi F. Compliance with referral of sick children : a survey in five districts of Afghanistan. *BMC Pediatr.* 2012; 12:1471–2431.
34. Rosenstock S, Katz J, Mullany LC, Khatry SK, Leclerq SC, Darmstadt GL, et al. Sex differences in morbidity and care-seeking during the neonatal period in rural southern Nepal. *J Heal Popul Nutr*. 2015; 33:1–10.
35. Getachew T, Abebe SM, Yitayal M, Persson LÅ, Berhanu D. Assessing the quality of care in sick child services at health facilities in Ethiopia. *BMC Health Serv Res.* 2020; 7:1–12.

For peer review only - http://bmjopen.bmj.com/site/about/guidelines.xhtml

36. Logaraj, M. Management of Referral Services through Computer Based Management System, *Nat.J.Res.Com.Med.* 2013; 2, 1–4.

37. Nanyonjo A, Bagorogoza B, Kasteng F, Ayebale G, Makumbi F, Tomson G. Estimating the cost of referral and willingness to pay for referral to higher-level health facilities : a case series study from an integrated community case management programme in Uganda. *BMC Health Serv Res*. 2015;15:1–10.

For peer review only

Legend to figure

Figure 1: Cumulative percentages of distance from health posts to the referral health centres, analysed based on linear distance from 158 health posts to their 128 referral health centres.

Figure 2. The mean percentage scores for five general service readiness domains of the surveyed 165 health posts analysed based on the WHO Service Availability and Readiness Assessment (SARA) reference manual (22).

Supplementary figure 1: Percentages of mean score of basic amenities by tracer items

Supplementary figure 2: Percentages of mean score of basic equipment by tracer items

Supplementary figure 3: Percentages of mean score of standard precaution for infection prevention by tracer items.

Supplementary figure 4: Percentages of mean score of essential medicines by tracer items.

For peer review only

List of tables

Table 1.Characteristics of health posts in four regions of Ethiopia (December 2018-February 2019) (n=165)

Characteristics	n	%
Region		
Amhara	70	42
Oromia	48	29
SNNPR[a]	22	13
Tigray	25	15
Number of health extension worker per health post		
1	35	21
2+	130	79
Availability of		
Registers for 0 – 59 days old children	148	90
Registers for 2 -59 months old children	156	95
Cell phone signals in the health post	135	82
Number of health posts that referred sick children in the last quarter	69	42
Means of communication used for the referral of last sick child		
Facility landline or mobile phone	3	2
Staff member's mobile phone	25	15
In person communication	13	8
No communication for last child referral	124	75

[a]SNNPR - Southern Nations, Nationalities, and Peoples Region

For peer review only

Table 2. Disease classification, recommended and performed referrals among children 0-59 months old seen at the surveyed 165 health posts, December 2018 – February 2019.

Age group	Disease classification	Referral guideline supports referral?	Number seen	Number (%) referred
0 – 59 days	Very preterm or very low birth weight	Yes	0	0
	Preterm or low birth weight	Yes	4	2 (50)
	Possible serious bacterial infection	Yes	42	15 (36)
	Severe dehydration	Yes	0	0
	Severe persistent diarrhea	Yes	1	0
	Dysentery	Yes	1	1 (100)
	Severe jaundice	Yes	1	0
	Local bacterial infection	No	59	5 (9)
	Some dehydration	No	2	1 (50)
	No dehydration	No	17	1 (6)
	Jaundice	No	1	0
	Malaria	No	0	0
	Feeding problem or low weight	No	45	2 (4)
	Classification not given	-	26	0
	Other[a]	No	30	12 (40)
	Total 0 – 59 days old		229	39 (17)
2 – 59 moths	Severe pneumonia/very severe disease	Yes	9	8 (89)
	Severe dehydration	Yes	2	1 (50)
	Severe persistent diarrhea	Yes	3	1 (33)
	Dysentery	Yes	9	6 (67)
	Very severe febrile disease	Yes	2	1 (50)
	Severe complicated measles	Yes	0	0
	Severe malnutrition	Yes	22	6 (27)
	Severe anaemia	Yes	0	0
	Pneumonia	No	478	16 (3)
	Some dehydration	No	186	9 (5)
	No dehydration	No	203	0
	Persistent diarrhea	No	28	2 (7)
	Malaria	No	12	0
	Fever, malaria unlikely	No	11	0
	Fever, no malaria	No	55	4 (7)
	Measles with eye/mouth complications	No	0	0

For peer review only

Age group	Disease classification	Referral guideline supports referral?	Number seen	Number (%) referred
	Measles	No	1	1 (100)
	Acute ear infection	No	7	3 (43)
	Chronic ear infection	No	1	0
	Moderate malnutrition	No	33	4 (12)
	Anaemia	No	1	0
	Others[b]	No	64	16 (25)
	Total 2 – 59 months old		1123	78 (7)

[a]Other – classification for 0 – 59 days included "infection unlikely" (12), "eye problems" (10), and PNC cases with referral outcome (8)

[b]Others – classification for 2 – 59 months old children included mainly "no pneumonia, cough" (42), and "eye problems" (6), growth monitoring with referral outcomes (16).

For peer review only

Table 3: Health post and child factors associated with referrals of 0 – 59 months sick young children in four regions of Ethiopia

Characteristics	Referral status of 0 – 59 days old sick young infants			Referral status of 2 – 59 months old children		
	Referred N=39 n (%)	Not referred N=190 n (%)	P-value	Referred N=39 n (%)	Not referred N=190 n (%)	P-value
The health post						
Mean score of basic Amenities						
0 to 33 percent	13 (24)	42 (76)	0.466	20 (13)	129 (87)	0.043
34 to 66 percent	9 (15)	50 (85)		19 (6)	292 (94)	
67 to 100 percent	17 (15)	98 (85)		39 (6)	624 (94)	
Mean score of basic equipment						
0 to 33 percent	2 (22)	7 (78)	0.825	3 (4)	73 (96)	0.512
34 to 66 percent	10 (19)	43 (81)		12 (8)	147 (92)	
67 to 100 percent	27 (16)	140 (84)		63 (7)	825 (93)	
Mean score of standard precaution						
0 to 33 percent	4 (9)	40 (91)	0.527	30 (8)	353 (92)	0.279
34 to 66 percent	14 (19)	59 (81)		31 (8)	355 (92)	
67 to 100 percent	21 (19)	91 (81)		17 (5)	337 (95)	
Rapid diagnostic test (RDT)						
0 to 33 percent	10 (17)	50 (83)	0.947	47 (9)	467 (91)	0.026
34 to 66 percent	0	0		0	0	
67 to 100 percent	29 (17)	140 (83)		31 (5)	578 (95)	
Mean score of essential medicines						
0 to 33 percent	1 (8)	11 (92)	0.026	17 (18)	77 (82)	0.001
34 to 66 percent	32 (21)	113 (79)		43 (6)	697 (94)	
67 to 100 percent	6 (8)	66 (92)		18 (6)	271 (94)	
Health post's overall service readiness index						
0 to 33 percent	0 (0)	1 (0)	0.737	7 (18)	31 (82)	0.193
34 to 66 percent	22 (19)	93 (81)		43 (7)	575 (93)	
67 to 100 percent	17 (15)	96 (85)		28 (6)	439 (94)	
Number of health extension worker per health post+						
1	2 (5)	39 (95)	0.024	20 (9)	196 (81)	0.264
2 or more	37 (20)	151 (80)		58 (6)	849 (94)	
Distance between health post and the nearest referral health centre						
Distance to the next referral site[b]						
0 - 8 kms	16 (21)	61 (79)	0.554			
8.01 - 20 kms	10 (12)	71 (88)				

For peer review only

>20 kms	13 (19)	57 (81)				
0 - 8 kms				24 (7)	340 (93)	
8.01 - 14 kms				25 (8)	301 (92)	0.699
>14 kms				22 (6)	362 (94)	
The child						
Region						
Amhara	3 (25)	9 (75)		34 (7)	428 (93)	
Oromia	10 (13)	65 (87)	0.722	26 (7)	342 (93)	0.691
SNNPR[a]	9 (21)	33 (79)		11 (8)	128 (92)	
Tigray	17 (17)	83 (83)		7 (5)	147 (95)	
Age						
1st week	14 (24)	45 (76)				
2 – 4 weeks	16 (16)	82 (84)	0.353			
5 – 8 weeks	9 (13)	63 (87)				
2 – 11 months				26 (8)	294 (92)	
12 – 23 months				28 (8)	325 (92)	0.249
24 – 59 months				24 (5)	426 (95)	
Sex						
Boy	18 (15)	106 (85)	0.395	40 (7)	550 (93)	0.810
Girl	21 (20)	84 (80)		38 (7)	495 (93)	
Disease classification						
Non-severe illnesses not requiring referral	21 (12)	159 (88)	0.013	56 (5)	1023 (95)	0.001
Severe illnesses requiring referral	18 (37)	31 (63)		22 (50)	22 (50)	

[a] SNNPR - Southern Nations, Nationalities, and Peoples Region

[b] GIS based data of distance from health post to health centre were missing for 0-59 days sick young infant (1) and 2-59 months sick children (49).

For peer review only

Table 4. Factors associated with referral of 0-59 days old infants from health post to higher levels in four Ethiopian regions, December 2018-February 2019. Logistic regression analyses. (N=228)[1]

Characteristic	Levels	Referred (n)	Not referred (n)	Crude OR (95% CI)	Adjusted OR (95% CI)[2]
The health post					
Facility owned landline or mobile phone	Not available	13	35	1.0	1.0
	Available	26	154	0.47 (0.17 – 1.28)	0.61 (0.13 – 2.80)
Mean score of essential medicine	0 to 33 percent	1	11	1.0	1.0
	34 to 66 percent	32	112	3.14 (0.99 - 9.99)	3.17 (0.42 - 24.19)
	67 to 100 percent	6	66	1.00 (0.24 – 4.14)	1.34 (0.10 – 18.80)
Number of health extension workers	1	2	39	1.0	1.0
	2 or more	37	150	4.81 (1.10 - 21.05)$^{+}$	5.63 (0.87 – 36.40)
Distance between health post and the nearest referral health centre					
Distance tertiles	0-8 km	14	60	1.0	1.0
	8.01-20 km	11	63	0.54 (0.18 – 1.60)	0.60 (0.18 - 2.04)
	20.01 or more km	14	66	0.87 (0.26 – 2.96)	1.02 (0.33 – 3.17)
The child					
Age	0-1 week	14	45	1.0	1.0
	2-4 weeks	16	82	0.63 (0.21 – 1.84)	0.35 (0.11 – 1.10)
	5 or more weeks	9	62	0.47 (0.15 - 1.46)	0.25 (0.10 - 0.64)$^{++}$
Gender	Boy	18	105	1.0	1.0
	Girl	21	84	1.46 (0.59 - 3.62)	1.12 (0.45 - 2.75)
Disease classification	Not severe	21	158	1.0	1.0
	Severe	18	31	4.37 (1.30 – 14.67)$^{+}$	6.72 (1.50 - 30.11)$^{+}$

[1]Records of 1 sick young infant of 0-59 days old were removed because of missing of data of distance from health post to the referral health centre.

[2] Multivariable logistic regression adjusted for region and clusters. The logistic regression model was tested using the Hosmer and Lemeshow test of the goodness-of-fit statistics, which suggested that the model was a good fit to the data as *p=0.860* (*>0.05*).

$^{+++}$ P-value < 0.001 $^{++}$ P-value < 0.01 $^{+}$ P-value < 0.05

For peer review only

Table 5. Factors associated with referral of 2-59 months old children from health post to higher levels in four Ethiopian regions, December 2018-February 2019. Logistic regression analyses. (N=1074)[1]

Characteristic	Levels	Referred (n)	Not referred (n)	Crude OR (95% CI)	Adjusted OR (95% CI)[2]
The health post					
Facility owned landline or mobile phone	Not available	44	792	1.0	1.0
	Available	27	211	2.30 (1.16 – 4.55)+	1.88 (0.94 – 3.76)
Mean score of basic amenities	0 to 33 percent	20	129	1.0	1.0
	34 to 66 percent	15	279	0.35 (0.13 – 0.89)+	0.38 (0.15 – 0.96)+
	67 to 100 percent	36	595	0.39 (0.17 – 0.89)+	0.40 (0.16 - 1.03)
Mean score of basic equipment	0 to 33 percent	2	64	1.0	1.0
	34 to 66 percent	12	147	2.61 (0.65 - 10.45)	3.49 (0.16 - 75.12)
	67 to 100 percent	57	792	2.30 (0.62 - 8.53)	2.38 (0.12 - 48.96)
Amoxicillin	Not available	27	152	1.0	1.0
	Available	44	851	0.29 (0.15 – 0.58)++	0.41 (0.19 - 0.88)+
Rapid diagnostic test for malaria	Not available	45	449	1.0	1.0
	Available	26	554	0.47 (0.26 – 0.83)+	0.18 (0.07 - 0.46)++
Mean score of overall service readiness[3]	0 to 33 percent	6	22	1.0	1.0
	34 to 66 percent	43	573	0.28 (0.04 – 2.05)	0.21 (0.03 – 1.53)
	67 to 100 percent	22	408	0.20 (0.03 – 1.49)	0.62 (0.07 – 5.60)
Number of health extension workers	1	20	196	1.0	1.0
	2 or more	51	807	0.62 (0.30 – 1.28)	1.01 (0.38 - 2.65)
Distance between health post and the nearest referral health centre					
Distance tertiles	0-8 km	24	321	1.0	1.0
	8.01-14 km	25	320	1.18 (0.54 - 2.56)	0.98 (0.43 – 2.21)
	14.01 or more km	22	362	0.86 (0.43 - 1.71)	0.71 (0.28 - 1.79)
The child					
Age	0-1 week	25	284	1.0	1.0
	2-4 weeks	24	317	0.86 (0.48 - 1.54)	0.97 (0.52 - 1.81)
	5 or more weeks	22	402	0.62 (0.36 - 1.07)	0.88 (0.45 - 1.68)
Gender	Boy	35	526	1.0	1.0
	Girl	36	477	1.13 (0.71 -1.81)	0.89 (0.52 - 1.52)
Disease classification	Not severe	50	981	1.0	1.0
	Severe	21	22	18.73 (9.21 – 38.09)+++	21.27 (7.51 - 60.23)+++

For peer review only

[1]Records of 1 sick young infant of 0-59 days old were removed because of missing of data of distance from health post to the referral health centre.

[2] Multivariable logistic regression adjusted for region and clusters. The logistic regression model was tested using the Hosmer and Lemeshow test of the goodness-of-fit statistics, which suggested that the model was a good fit to the data as *p=0.500* (*>0.05*).

[3] Overall facility readiness was computed while the individual domains and items were excluded, to avoid the multi-collinearity problem.

+++ P-value < 0.001 ++ P-value < 0.01 + P-value < 0.0

For peer review only

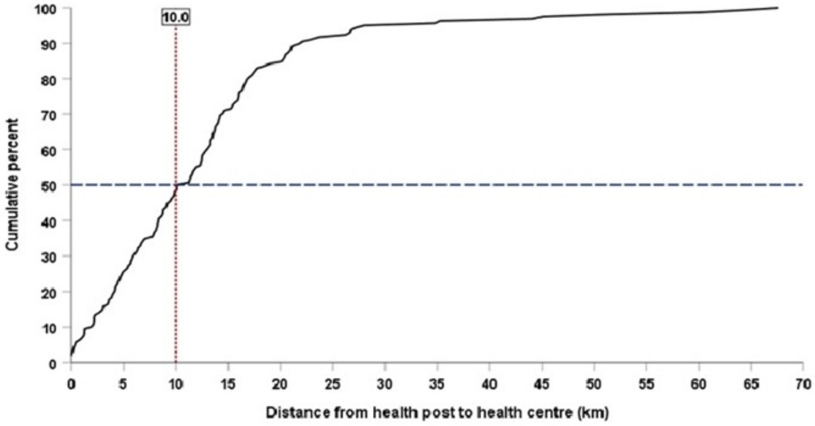

216x121mm (96 x 96 DPI)

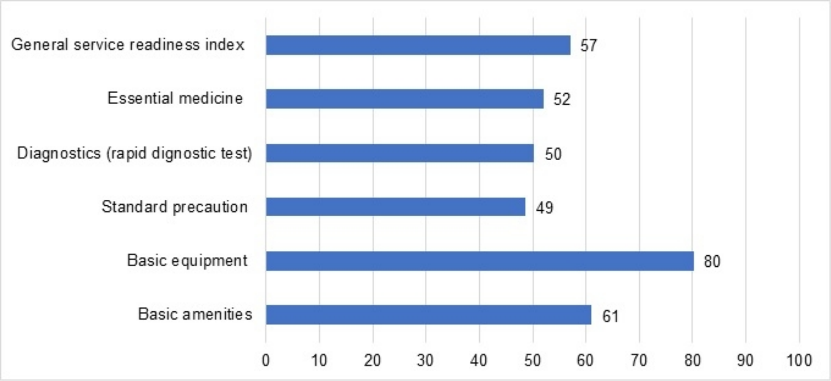

216x121mm (96 x 96 DPI)

List of supporting tables

Supplementary table 1: Individual health post readiness factors associated with referrals of 0-59 months sick children in four regions of Ethiopia

Characteristics	Referral status of 0 – 59 days sick young infants			Referral status of 2 – 59 months old children		
	Referred N=39 n (%)	Not referred N=190 n (%)	P-value	Referred N=78 n (%)	Referred N=1045 n (%)	P-value
Availability of basic amenities						
Water source						
Not available	15 (22)	53 (78)	0.296	30 (8)	356 (92)	0.558
Available	24 (15)	137 (85)		48 (7)	689 (93)	
Electric power						
Not available	18 (17)	91 (83)	0.886	51 (8)	602 (92)	0.257
Available	21 (18)	99 (82)		27 (6)	443 (94)	
Facility owned toilet						
Not available	6 (18)	27 (82)	0.880	14 (7)	207 (93)	0.981
Available	33 (17)	163 (83)		64 (7)	838 (93)	
Facility owned landline or mobile phone						
Not available	35 (19)	153 (81)	0.133	51 (6)	828 (94)	0.038
Available	4 (10)	37 (90)		27 (11)	217 (89)	
Availability of basic equipment						
Thermometer						
Not available	8 (14)	49 (86)	0.594	12 (5)	228 (95)	0.207
Available	31 (18)	141 (82)		66 (8)	817 (92)	
Child weight scale						
Not available	5 (13)	34 (87)	0.526	20 (9)	214 (91)	0.388
Available	34 (18)	156 (82)		58 (7)	831 (93)	
Stethoscope						
Not available	18 (19)	79 (81)	0.709	35 (8)	399 (92)	0.336
Available	21 (16)	111 (84)		43 (6)	646 (94)	
MUAC tape measure						
Not available	1 (100)	0	0.040	0 (0)	14 (100)	0.701
Available	38 (17)	190 (83)		78 (7)	1031 (93)	
Availability Diagnostics						
Rapid diagnostic test (RDT)						
Not available	10 (17)	50 (84)	0.947	47 (9)	467 (91)	0.026
Available	29 (17)	140 (82)		31 (5)	578 (95)	
Availability of essential medicines						
Vitamin A						
Not available	9 (27)	25 (73)	0.211	11 (5)	207 (95)	0.214
Available	30 (15)	165 (85)		67 (7)	838 (93)	
Amoxicillin						

For peer review only

For peer review only - http://bmjopen.bmj.com/site/about/guidelines.xhtml

	Not available	6 (14)	36 (86)	0.745	28 (15)	159 (85)	0.001
	Available	33 (18)	154 (82)		50 (5)	886 (95)	
Gentamycin							
	Not available	20 (14)	127 (86)	0.201			
	Available	19 (23)	63 (77)				
Tetracycline eye ointment							
	Not available	26 (19)	110 (81)	0.457	46 (8)	567 (92)	0.554
	Available	13 (14)	80 (86)		32 (6)	478 (94)	
Oral rehydration solution (ORS)							
	Not available	2 (13)	13 (87)	0.627	10 (17)	48 (83)	0.048
	Available	37 (17)	177 (83)		68 (6)	997 (94)	
Zinc-ORS							
	Not available				69 (7)	947 (93)	0.608
	Available				9 (8)	98 (92)	
Zinc							
	Not available				16 (9)	152 (91)	0.331
	Available				62 (7)	893 (93)	
Coartem							
	Not available	18 (20)	71 (80)	0.494	47 (9)	507 (91)	0.087
	Available	21 (15)	119 (85)		31 (5)	535 (95)	
Plumpynut							
	Not available	8 (23)	27 (77)	0.404	30 (8)	372 (92)	0.688
	Available	31 (16)	163 (84)		48 (7)	673 (93)	
BP100							
	Not available	38 (17)	180 (83)	0.012	77 (7)	993 (93)	0.107
	Available	1 (9)	10 (91)		1 (2)	52 (98)	

For peer review only

Supplementary table 2. Adherence to referral management guidelines for sick young infants and older children

Age group	Observed	Management guideline	
		Referral advised	Referral not advised
0-59 days	Referred	18	21
	Not referred	31	159
2-59 months	Referred	22	56
	Not referred	22	1023

*Shadowed cells indicate referral management that does not adhere to guideline

For peer review only

Supplementary table 3: Logistic regression for correct management of sick young infants according to guidelines

Characteristic	Levels	Management according to guideline		Crude OR (95% CI)	Adjusted OR (95% CI)
		Correct (n)	Not correct (n)		
Facility-owned landline or mobile phone	Not available	145	42	1.0	1.0
	Available	31	10	0.90 (0.26 – 3.07)	1.12 (0.27 – 4.68)
Mean score of essential medicine	0 to 33 percent	7	5	1.0	1.0
	34 to 66 percent	112	32	2.50 (0.83 – 7.51)	4.49 (0.93 – 21.77)
	67 to 100 percent	57	15	2.71 (0.51 – 14.46)	12.95 (1.49 – 112.82)+
Number of health extension workers	1	27	14	1.0	1.0
	2 or more	149	38	2.03 (0.37 – 11.28)	8.51 (0.83 – 86.98)
Distance tertiles	0-8 km	54	23	1.0	1.0
	8.01-20 km	63	18	1.49 (0.37 – 6.03)	1.01 (0.17 – 6.13)
	20.01 or more km	59	11	2.28 (0.56 – 9.30)	0.58 (0.08 – 4.20)
Age	0-1 week	49	10	1.0	1.0
	2-4 weeks	69	29	0.49 (0.15 – 1.58)	0.57 (0.15 – 2.24)
	5 or more weeks	58	13	0.91 (0.26 – 3.23)	1.37 (0.28 - 6.76)
Gender	Boy	95	28	1.0	1.0
	Girl	81	24	0.99 (0.47 – 2.11)	1.19 (0.55 - 2.58)

+++ P-value < 0.001 ++ P-value < 0.01 + P-value < 0.05

For peer review only

Supplementary table 4: Logistic regression for correct management of sick older children according to guidelines

Characteristic	Levels	Management according to guideline Correct (n)	Not correct (n)	Crude OR (95% CI)	Adjusted OR (95% CI)
Age	0-1 week	282	27	1.0	1.0
	2-4 weeks	315	26	1.16 (0.65 – 2.06)	1.24 (0.70 – 2.18)
	5 or more weeks	402	22	1.75 (0.95 – 3.23)	1.78 (0.93 - 3.40)
Gender	Boy	524	37	1.0	1.0
	Girl	475	38	0.88 (0.56 – 1.39)	0.97 (0.61 - 1.55)
Amoxicillin	Not available	153	26	1.0	1.0
	Available	846	49	2.93 (1.48 – 5.82)++	2.74 (1.40 – 5.35)++
Distance tertiles	0-8 km	340	24	1.0	1.0
	8.01-14 km	298	28	0.75 (0.36 – 1.58)	0.72 (0.28 – 1.82)
	14.01 or more km	361	23	1.11 (0.54 – 2.27)	0.96 (0.44 – 2.05)
Mean score of basic amenities	0 to 33 percent	130	19	1.0	1.0
	34 to 66 percent	278	16	2.54 (1.01 – 6.39)+	1.97 (0.71 – 5.49)
	67 to 100 percent	591	40	2.16 (0.93 – 5.02)	1.41 (0.56 – 3.56)
Mean score of basic equipment	0 to 33 percent	61	5	1.0	1.0
	34 to 66 percent	141	18	0.64 (0.21 – 1.93)	0.96 (0.12 – 7.57)
	67 to 100 percent	797	52	1.26 (0.45 – 3.47)	2.46 (0.29 – 20.54)
Mean score of diagnostics	0 to 33 percent	448	46	1.0	1.0
	67 to 100 percent	551	29	1.95 (1.10 – 3.46)+	4.59 (1.79 - 11.75)++
Mean score of overall readiness	0 to 33 percent	21	7	1.0	1.0
	34 to 66 percent	570	46	4.13 (0.68 – 25.02)	3.19 (0.43 - 23.61)
	67 to 100 percent	408	22	6.18 (1.01 – 37.87)	1.59 (0.14 – 18.00)
Number of health extension workers	1	193	23	1.0	1.0
	2 or more	806	52	1.84 (0.92 – 3.72)	1.52 (0.77 – 3.02)

+++ P-value < 0.001 ++ P-value < 0.01 + P-value < 0.

For peer review only

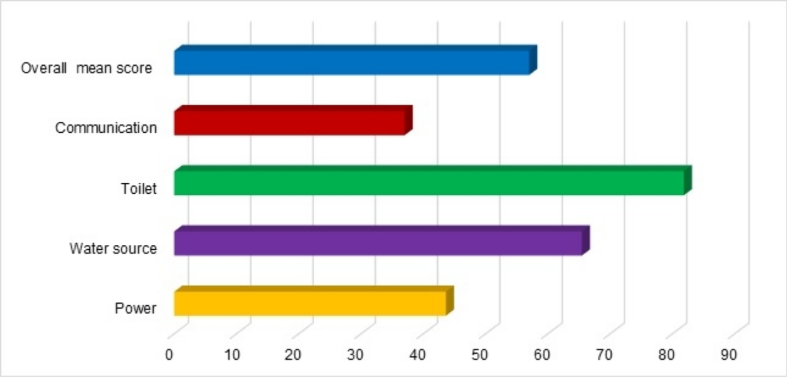

216x121mm (96 x 96 DPI)

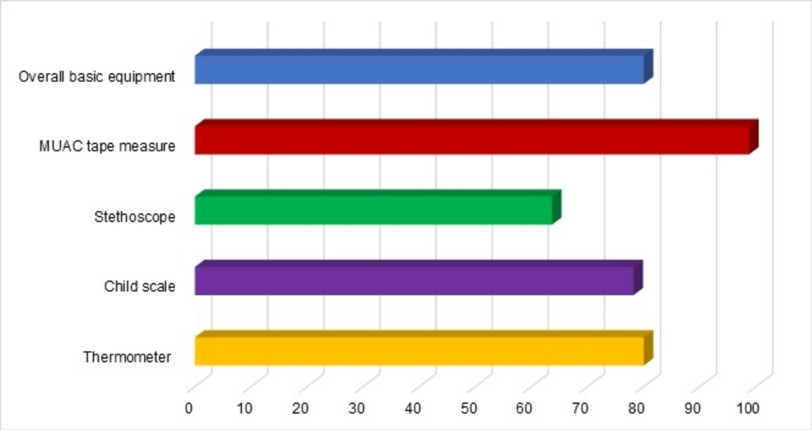

216x121mm (96 x 96 DPI)

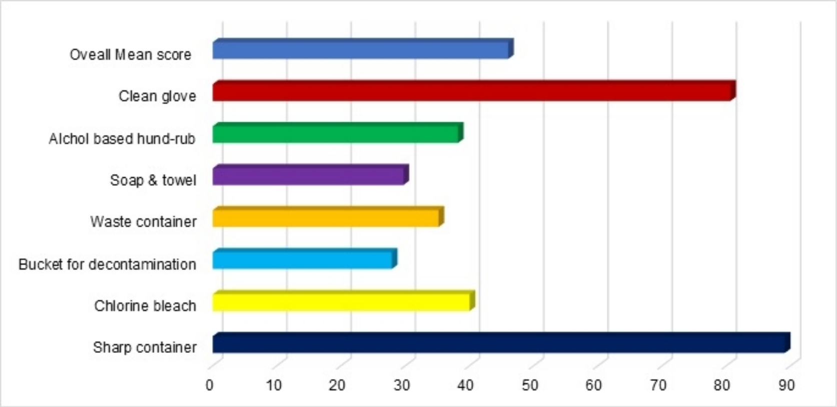

216x121mm (96 x 96 DPI)

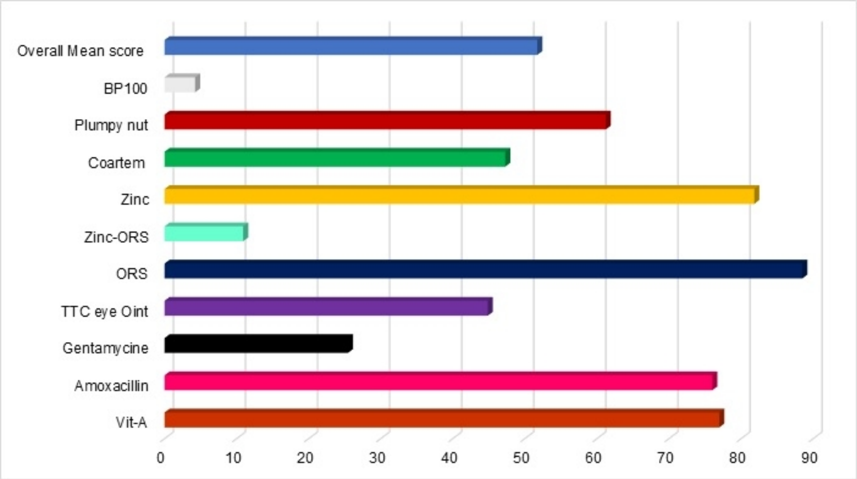

216x121mm (96 x 96 DPI)

STROBE 2007 (v4) Statement—Checklist of items that should be included in reports of *cross-sectional studies*

Section/Topic	Item #	Recommendation	Reported on page #
**Title and abstract**	1	(*a*) Indicate the study's design with a commonly used term in the title or the abstract	1
		(*b*) Provide in the abstract an informative and balanced summary of what was done and what was found	2
**Introduction**			
Background/rationale	2	Explain the scientific background and rationale for the investigation being reported	3
Objectives	3	State specific objectives, including any prespecified hypotheses	5
**Methods**			
Study design	4	Present key elements of study design early in the paper	5
Setting	5	Describe the setting, locations, and relevant dates, including periods of recruitment, exposure, follow-up, and data collection	5
Participants	6	(*a*) Give the eligibility criteria, and the sources and methods of selection of participants	6
Variables	7	Clearly define all outcomes, exposures, predictors, potential confounders, and effect modifiers. Give diagnostic criteria, if applicable	8
Data sources/ measurement	8*	For each variable of interest, give sources of data and details of methods of assessment (measurement). Describe comparability of assessment methods if there is more than one group	7
Bias	9	Describe any efforts to address potential sources of bias	8
Study size	10	Explain how the study size was arrived at	6 – 7
Quantitative variables	11	Explain how quantitative variables were handled in the analyses. If applicable, describe which groupings were chosen and why	7 – 8
Statistical methods	12	(*a*) Describe all statistical methods, including those used to control for confounding	8
		(*b*) Describe any methods used to examine subgroups and interactions	8 - 9
		(*c*) Explain how missing data were addressed	29 - 30
		(*d*) If applicable, describe analytical methods taking account of sampling strategy	8
		(*e*) Describe any sensitivity analyses	-
**Results**			

For peer review only

Participants	13*	(a) Report numbers of individuals at each stage of study—eg numbers potentially eligible, examined for eligibility, confirmed eligible, included in the study, completing follow-up, and analysed	9
		(b) Give reasons for non-participation at each stage	-
		(c) Consider use of a flow diagram	-
Descriptive data	14*	(a) Give characteristics of study participants (eg demographic, clinical, social) and information on exposures and potential confounders	9
		(b) Indicate number of participants with missing data for each variable of interest	8, 28, 29, 30
Outcome data	15*	Report numbers of outcome events or summary measures	10, 25
Main results	16	(*a*) Give unadjusted estimates and, if applicable, confounder-adjusted estimates and their precision (eg, 95% confidence interval). Make clear which confounders were adjusted for and why they were included	11, 29, 30
		(*b*) Report category boundaries when continuous variables were categorized	10, 29, 30
		(*c*) If relevant, consider translating estimates of relative risk into absolute risk for a meaningful time period	N/A
Other analyses	17	Report other analyses done—eg analyses of subgroups and interactions, and sensitivity analyses	12
**Discussion**			
Key results	18	Summarise key results with reference to study objectives	12
Limitations	19	Discuss limitations of the study, taking into account sources of potential bias or imprecision. Discuss both direction and magnitude of any potential bias	12 - 13
Interpretation	20	Give a cautious overall interpretation of results considering objectives, limitations, multiplicity of analyses, results from similar studies, and other relevant evidence	13 - 14
Generalisability	21	Discuss the generalisability (external validity) of the study results	13
**Other information**			
Funding	22	Give the source of funding and the role of the funders for the present study and, if applicable, for the original study on which the present article is based	16

*Give information separately for cases and controls in case-control studies and, if applicable, for exposed and unexposed groups in cohort and cross-sectional studies.

Note: An Explanation and Elaboration article discusses each checklist item and gives methodological background and published examples of transparent reporting. The STROBE checklist is best used in conjunction with this article (freely available on the Web sites of PLoS Medicine at http://www.plosmedicine.org/, Annals of Internal Medicine at http://www.annals.org/, and Epidemiology at http://www.epidem.com/). Information on the STROBE Initiative is available at www.strobe-statement.org.

For peer review only

BMJ Open

Factors associated with the referral of children with severe illnesses at primary care level in Ethiopia: a cross-sectional study.

Journal:	*BMJ Open*
Manuscript ID	bmjopen-2020-047640.R1
Article Type:	Original research
Date Submitted by the Author:	22-Mar-2021
Complete List of Authors:	Beyene, Habtamu; Hawassa University, College of Medicine and Health Sciences; Southern Nations Nationalities and Peoples' Region, Regional Health Bureau<br>Kassa, Dejene; Hawassa University, College of Medicine and Health Sciences, School of Public Health<br>Tadele, Henok; Addis Ababa University, College of Health Sciences, Department of Paediatrics and Child Health<br>Persson, Lars; London School of Hygiene and Tropical Medicine Faculty of Infectious and Tropical Diseases; Ethiopian Public Health Institute<br>Defar, Atkure ; Ethiopian Public Health Institute, Health System and Reproductive Health Research Directorate ; University of Gondar, Epidemiology and Biostatistics, Institute of Public Health, College of Medicine and Health Sciences<br>Berhanu, Della; London School of Hygiene and Tropical Medicine Faculty of Infectious and Tropical Diseases; Ethiopian Public Health Institute
**Primary Subject Heading**:	Paediatrics
Secondary Subject Heading:	Public health
Keywords:	Organisation of health services < HEALTH SERVICES ADMINISTRATION & MANAGEMENT, Quality in health care < HEALTH SERVICES ADMINISTRATION & MANAGEMENT, Community child health < PAEDIATRICS, EPIDEMIOLOGY

SCHOLARONE™
Manuscripts

I, the Submitting Author has the right to grant and does grant on behalf of all authors of the Work (as defined in the below author licence), an exclusive licence and/or a non-exclusive licence for contributions from authors who are: i) UK Crown employees; ii) where BMJ has agreed a CC-BY licence shall apply, and/or iii) in accordance with the terms applicable for US Federal Government officers or employees acting as part of their official duties; on a worldwide, perpetual, irrevocable, royalty-free basis to BMJ Publishing Group Ltd ("BMJ") its licensees and where the relevant Journal is co-owned by BMJ to the co-owners of the Journal, to publish the Work in this journal and any other BMJ products and to exploit all rights, as set out in our licence.

The Submitting Author accepts and understands that any supply made under these terms is made by BMJ to the Submitting Author unless you are acting as an employee on behalf of your employer or a postgraduate student of an affiliated institution which is paying any applicable article publishing charge ("APC") for Open Access articles. Where the Submitting Author wishes to make the Work available on an Open Access basis (and intends to pay the relevant APC), the terms of reuse of such Open Access shall be governed by a Creative Commons licence – details of these licences and which Creative Commons licence will apply to this Work are set out in our licence referred to above.

Other than as permitted in any relevant BMJ Author's Self Archiving Policies, I confirm this Work has not been accepted for publication elsewhere, is not being considered for publication elsewhere and does not duplicate material already published. I confirm all authors consent to publication of this Work and authorise the granting of this licence.

For peer review only

Factors associated with the referral of children with severe illnesses at primary care level in Ethiopia: a cross-sectional study.

Short run title: Referral of sick children in Ethiopia

Habtamu Beyene[1, 2], Dejene Hailu[2], Henok Tadele[3], Lars Åke Persson[4, 5], Atkure Defar[5, 6], Della Berhanu[4, 5]

Corresponding author:

Habtamu Beyene; MSc, BSc

College of Medicine and Health Sciences, School of Public Health

Hawassa University,

Hawassa, Ethiopia

Tel: +251-911-003346

Email: habtamu3346@yahoo.com

Co-authors:

Dr. Dejene Hailu Kassa; PhD, Assoc. Prof. of Public health, dejenkassa@yahoo.com

Dr. Henok Tadele; MD, Assoc. Prof. of Pediatrics & Child Health; henny_2007@yahoo.com

Prof. Lars Åke Persson; MD, PhD, lars.persson@lshtm.ac.uk

Atkure Defar; MPH-E, MBA, atkuredefar@gmail.com

Dr. Della Berhanu; PhD, della.berhanu@lshtm.ac.uk

For peer review only

ABSTRACT

Context and objective: Ethiopia's primary care has a weak referral system for sick children. We aimed to identify health post and child factors associated with referrals of sick children 0-59 months of age and evaluate the healthcare providers' adherence to referral guidelines.

Design: A cross-sectional facility-based survey.

Setting: This study included data from 165 health posts in 52 districts in four Ethiopian regions collected from December 2018 to February 2019. The data included interviews with health extension workers, assessment of health post preparedness, recording of GPS-coordinates of the health post and the referral health centre, and reviewing registers of sick children treated during the last three months at the health posts. We analysed the association between the sick child's characteristics, health post preparedness, and distance to the health centre with referral of sick children by multivariable logistic regressions.

Outcome measure: Referral to the nearest health centre of sick young infants aged 0-59 days and sick children 2-59 months.

Results: The health extension workers referred 39/229 (17%) of the sick young infants and 78/1123 (7%) of the older children to the next level of care. Only 18 (37%) sick young infants and 22 (50%) 2-59 months children that deserved urgent referral according to guidelines were referred. The leading causes of referral were possible serious bacterial infection and pneumonia. Those being classified as a severe disease were referred more frequently. The availability of basic amenities (AOR = 0.38, 95% CI: 0.15 – 0.96), amoxicillin (AOR = 0.41, 95% CI: 0.19 - 0.88) and rapid diagnostic test (AOR = 0.18, 95% CI: 0.07 - 0.46) were associated with less referral in the older age group.

Conclusion: Few children with severe illness were referred from health posts to health centres. Improving the health posts' medicine and diagnostic supplies may enhance adherence to referral guidelines and ultimately reduce child mortality.

Key words: health extension workers, referral, severe illnesses, sick child, referral factors.

For peer review only

Strengths and limitations of this study

- Based on secondary analysis of register data from 165 health posts in 52 districts of the four most populous regions of Ethiopia, this study reports primary care referral patterns and associated factors in rural areas.
- This study used objectively measured data based on the WHO health service availability and readiness assessment tool and information obtained from the surveyed health posts compared with the national guidelines on referral.
- Using a cross-sectional study design, we cannot prove causal relationships between the health post readiness, child factors, and referral of sick children observed in this study.
- Referral data were based on register reviews; Ethiopian facility registers are job aids to assess, classify, and treat sick children – a fact that may have enhanced completeness and reduced reporting bias.
- The study findings do not give a complete picture of the country's referral system as we limited the analysis to the primary healthcare level, and we did not collect data from parents of the referred children.

For peer review only

INTRODUCTION

Globally, nearly 5.3 million children below the age of 5 years died in 2018. Of these deaths, 47% occurred during the first month of life. More than half of this mortality occurred in Sub-Saharan Africa. Infectious diseases remained the leading causes of death (1).

Ethiopia achieved a significant reduction in child mortality during the Millennium Development Goals era from 1990 to 2015 (2). However, under-five and neonatal mortality rates remain high with 55 and 30 deaths per 1,000 live births, respectively (3). Lower respiratory tract infection, diarrhoeal diseases, and neonatal causes (mainly preterm birth complications, asphyxia, and neonatal sepsis), were the leading causes of death (4).

The integrated case management of childhood illnesses and the community based newborn care programmes are, if delivered with quality, cost-effective in reducing under-five deaths (5,6). Referral is included as a critical element in these programmes (7). In Ethiopia, the integrated community case management programme was initiated in 2010 (8). This programme trained health extension workers to assess, classify, and treat sick children 2-59 months of age with pneumonia, diarrhoea, malnutrition, and other common illnesses and refer severe cases to health centres according to guidelines (9). In 2013, the Community-Based Newborn Care programme was initiated. Health extension workers were trained to provide pre-referral antibiotic treatment for young infants (0-59 days) with possible severe bacterial infection and refer those to a higher-level facility. If a referral was not possible, the health workers were instructed to provide a seven-day antibiotic treatment. These interventions have been implemented in Ethiopia within the existing Health Extension Program (6,10).

A referral is defined as “a process in which a health worker at one level of the health system, having insufficient resources (drugs, equipment, skills) to manage a clinical condition, seeks the assistance of a better or differently resourced facility at the same or higher level to assist in or take over the management of the client’s case” (11). A well-functioning referral system is an essential part of the health system and an indicator of the quality of care provided at health facilities (7). The referral pattern may reflect the healthcare

For peer review only

workers' ability to identify signs of severe illness at an early stage (12). For effective referral care, a pre-requisite is also that the higher-level facility is ready to receive and treat these children appropriately (7).

In a previous study, we showed that the Ethiopian health extension workers referred only a fraction of the severely sick young infants and under-five children that deserved urgent referral to health centres. Generally, referral logistics were weak (13). Other studies in low-income countries have shown that the distance to the referral site, the severity of the child's illnesses (14), and the availability of referral logistics (15,16) may influence severely sick children's referral.

This study aimed to identify factors associated with the referral of severely ill children aged 0-59 months and healthcare providers' adherence to referral guidelines in Ethiopia's primary health care system. Specifically, we assessed the referral of sick children 0-59 months from health post to the next higher level of health care in relation to guidelines. We analysed factors associated with referral care, including child characteristics, health post preparedness, and distance to the next-level health facility.

METHODS

Study design and settings

This study was based on data from the evaluation of Optimizing the Health Extension Programme intervention. This intervention was a collaborative effort between the Government of Ethiopia and implementing partners to increase the use and quality of community-based newborn care and integrated community case management of childhood illnesses in four Ethiopian regions (17).

We did a secondary analysis of data from the endline cross-sectional survey conducted in 26 districts, where the Optimizing the Health Extension Programme intervention was implemented, and in 26 comparison districts without the intervention. These districts were found in four major Ethiopian regions, namely Amhara, Oromia, Southern Nations, Nationalities and Peoples (SNNP), and Tigray. The survey was conducted from December 2018 to February 2019, after the implementation of the intervention.

For peer review only

In Ethiopia, primary health care services are provided within the district health system, comprised of five primary health care units and a district hospital serving around 100,000 people. Each primary health care unit consists of five health posts and their referral health centre. The health post is typically staffed with two health extension workers and serves a population of 5000 people (18).

The referral care of sick children 0-59 months of age from health posts to the next level was assessed in relation to the treatment guidelines for 0-59 days old sick young infants (community-based newborn care), and 2 – 59 months old children (integrated community case management).

According to these guidelines, babies 0-59 days who are very preterm (< 32 weeks), very low birth weight (< 1500gm), preterm (32-37 weeks), low birth weight (1500-2500gm), have a possible severe bacterial infection, severe jaundice, severe dehydration, severe persistent diarrhoea, or dysentery require an urgent referral. Similarly, children 2-59 months with a possible severe bacterial infection, severe dehydration, severe pneumonia, possible severe bacterial infections, severe persistent diarrhoea, very severe febrile disease, severe complicated measles, mastoiditis, severe anaemia, and complicated severe acute malnutrition require an urgent referral (19).

Study population and sample

The primary study had household and health facility modules to evaluate the Optimizing the Health Extension Programme intervention (20). We used the 2007 Ethiopian census to select 200 enumeration areas proportional to the population size of the 52 districts included in the study. In the main study, households were randomly selected to represent the enumeration areas. The health posts serving the selected households were included in this study. All records of 0-59 days old sick young infants, who sought care in three months prior to the survey, and records of the most recent ten 2-59 months sick children, who also sought care, were reviewed from treatment registers at the health post. Children without any illness who visited the health posts for postnatal care, vaccination, and growth monitoring were excluded from the analysis.

The sample size for the main study was based on the requirement that the baseline and end-line surveys should measure changes of a fixed number of percentage points of selected indicators for care-seeking and treatment for a sick child, based on household interviews.

Data collection

The questionnaires used in this study were adapted from existing large-scale survey tools, such as the Demographic and Health Surveys and the Service Provision Assessment survey (21). Questionnaires were translated into three local languages (Amharic, Afan Oromo and Tigrigna) and pre-tested. All data were collected using tablet computers.

Out of the eligible 200 clusters, 19 clusters were excluded from the study due to civil unrest. Trained health professionals and supervisors collected the data.

We collected data on the number of health extension workers at the health posts, availability of amenities, equipment, supplies, diagnostics, and medicines. Geographic Positioning System (GIS) coordinates were assessed at health posts and referral health centres. We reviewed registers of 0-59 days and 2-59 months old children and collected background data on their symptoms, disease classifications, treatment, and referral status.

Patient and Public Involvement: Not applicable (patients were not involved in this study).

Data analysis

A descriptive analysis was performed, including frequencies, percentages, and means. We characterized the health posts, the distance from health posts to the referral health centres, the proportion of health posts with service readiness tracer items and referral logistics for childhood referral care, and the referral of sick children of 0-59 months from health posts to higher levels in relation to the treatment guidelines.

We used health posts and their respective referral health centres' GPS reading to calculate the two's straight-line distance. The health post's facility readiness was assessed using the World Health Organization service

For peer review only

availability and readiness assessment (SARA) reference manual (22). The general health facility service readiness has five domains, which include different tracer indicators, items, and services. The first is basic amenities, including four items: communication, access to toilet facilities for clients, water source, and power supply. The second is basic equipment with four items: infant or child scale, thermometer, functional stethoscope, and mid-upper arm circumference tape measure. The third is standard precautions for infection prevention with seven items: sharps container, chlorine bleach, bucket for decontamination solution, contaminated waste container, soap, and a towel or hand rub, alcohol-based hand rub, and clean gloves. The fourth is the basic diagnostic capacity with a rapid diagnostic test for malaria. The last is essential medicines with ten items: vitamin A, tetracycline eye ointment, gentamycin, amoxicillin, oral rehydration solution, zinc-oral rehydration solution combined, zinc, and the ready-to-use- therapeutic foods (plumpy nut and BP100). Finally, the combined facility service readiness was assessed using the general service readiness index by calculating five domains' mean score.

The attributes of the referred sick children, their symptoms, disease classification, and treatment were analysed based on the existing treatment guidelines (19). Accordingly, the proportion of sick children with severe illnesses that require an urgent referral, non-severe illnesses that do not require urgent referral, the numbers referred and causes of their referrals were analysed. The referral status was linked with the facility readiness, and other health post factors, including the distance to the referral facility.

Referral of 0-59 days old sick young infants, and 2-59 months old children were the outcomes separately dichotomized as "yes" (referred) or "no" (not referred). Chi-square tests were performed to assess whether the child's characteristics, health post preparedness, and the distance to the next level health facility were associated with a sick child's referral. Relevant factors with statistically significant association to the referral of sick children in the bivariate analysis were entered into a multivariable logistic regression analysis to identify referral predictors. Adjustments were made for clustering and whether the cluster was an Optimizing the Health Extension Program intervention or comparison area. Odds ratios (ORs) with 95% confidence intervals

For peer review only

(CIs) were calculated to measure the association between the predictor and outcome variables. Additionally, adherence to referral guidelines for sick young infants and older children was dichotomised as “yes” (managed according to referral guideline) or “no” (not managed according to referral guideline). Factors that had statistically significant association with referrals were then assessed in bivariate and multivariable logistic regression analyses to identify predictors of adherence to correct referral practices.

The Census and Survey Processing System (CSPro) software (United States Census Bureau, Washington DC, USA), was used to enter and edit survey data. Statistical analysis and tests were done using SPSS version 25 (SPSS, IBM Corp, New York, USA).

RESULTS

We extracted data from treatment registers at 165 health posts serving 181 enumeration areas. Information on the assessment, treatment and referral of 229 sick young infants of 0 – 59 days and 1123 sick children aged 2-59 months was collected from the registers. Out of the 165 health posts, 48 had one or more registered 0-59 days old sick young infants, and 147 had registered one or more 2-59 months old sick children in the three months preceding the survey.

Characteristics of health posts

Nearly 80% of health posts were staffed with two or more health extension workers, while the rest had only one. Almost all health posts had sick child registers, and 42% had referred at least one sick child to a health centre in the quarter before the survey. A cell phone signal was available in most of the surveyed health posts. Still, most health workers had neither used mobile phones nor other communication means for their most recent referral of a sick child (Table 1). Half of the surveyed health posts were found within 10 kilometres from the next level referral health centre (Figure 1). There were missing distance data for seven health posts.

For peer review only

Health post readiness status

The readiness of health posts to provide health services was assessed for five general service readiness domains. The average readiness score was 61% for basic amenities, 80% for basic equipment, 49% for standard precaution for infection prevention, 50% for basic diagnostic capacity, and 52% for essential medicines (Figure 2). A significant proportion of the health posts lacked readiness to provide the required service for sick children, shown by an overall service readiness index of 57%. Moreover, nearly half of the health posts did not have the required essential medicines including those needed to treat possible severe bacterial infection of 0-59 days sick young infants. The items included in these scores are shown in supplementary figure 1 – 4.

Referral of sick children

During the last three months before the survey, 39 (17%) of the 229 examined 0 – 59 days old sick young infants were referred from the health posts to the nearest health centres. Similarly, 78 (7%) of the 1123 sick children aged 2 – 59 months old had been referred. Possible serious bacterial infection and pneumonia were the leading causes of referral among 0 – 59 days old and 2 – 59 months old children, respectively (Table 2).

Cross-tabulation of the four facility readiness domains and their respective individual components are shown in supplementary table 1 and 2, respectively.

Factors associated with referral

Sick young infants aged 0-59 days with severe illnesses were seven times more likely to be referred to the next referral level, as compared to those with non-severe illnesses (95% CI: 1.50 - 30.11) (Table 3). Sick young infants in the later weeks of life were less likely to be referred than those in the first week (AOR = 0.25, 95% CI: 0.10 - 0.64). Neither the sex of the sick child nor the distance from health post to referral health centre were associated with referral (Table 3). A higher proportion of sick young infants was referred from health posts served by two or more health extension workers than those served by only one (p=0.024) (Supplementary table 1).

For peer review only

Also, sick children aged 2-59 months with severe illness were more likely to be referred than those with non-severe illness (AOR = 21.27, 95% CI: 7.51 - 60.23) (Table 3). Health posts that had a better score for basic amenities were less likely to refer 2-59 months sick children to the next level than those without (AOR= 0.38, 95% CI: 0.15 – 0.96). Health posts with amoxicillin (AOR = 0.41, 95% CI: 0.19 - 0.88) and rapid diagnostic tests (AOR = 0.18, 95% CI: 0.07 – 0.46) were less likely to refer 2-59 months old children than those without these medicines and diagnostic kit.

The number of health extension workers, age and sex of the child and distance from health post to the referral health centre were not associated with referrals of 2-59 months old children.

Management according to guideline

The adherence to referral guidelines of sick young infants and older children is shown in supplementary table 3. Among the sick 229 young infants, 21 children were referred when a referral was not advised, and 31 children were treated at the health post when a referral was recommended. The remaining 177 (77%) were managed according to referral guidelines. Of the factors associated with a referral, essential medicines were also associated with adhering to referral guidelines for young infants. Those in the higher tertile adhered more to referral guidelines (AOR= 13, 95% CI: 1.5-112.6). Among the 1123 sick older children, 56 were referred when a referral was not advised, and 22 children were treated at the health post when a referral was required. The remaining 945 (93%) were managed according to referral guidelines. Facilities with amoxicillin and diagnostics were more likely to adhere to referral guidelines (amoxicillin AOR = 2.8, 95% CI: 1.3-5.5 and diagnostics AOR = 4.7, 95% CI: 1.8-11.9). Data for this analysis are shown in supplementary tables 4 and 5.

DISCUSSION

Our study from four major Ethiopian regions showed that health extension workers referred only around one-third of the sick young infants 0-59 days old and half of the 2-59 months old children that should be referred, according to guidelines. The leading diagnoses for referral were possible severe bacterial infections in young infants and pneumonia in older children. As expected, conditions classified as severe were referred more

For peer review only

frequently than non-severe cases. The tendency to refer was associated with some characteristics. For 0-59 days old infants, young neonates were referred more often than a bit older young infants. Health posts with basic amenities, amoxicillin, and rapid diagnostic tests were less inclined to refer older sick children. Within the 2-59 months group, age was not associated with referral. There was no sex difference in referral. Further, the straight-line distance from the health post to the health centre was not associated with referrals.

The availability of medicine was assessed for the day of the survey, while records of children seen up to three months prior to the survey were included from registers and as such caution should be taken when interpreting the association between drug availability and referral adherence. Due to the cross-sectional study design, we cannot prove any causal relationship between the health post and child factors that were associated with a referral of sick children. Further, we have analysed referral from the first primary care level, and are therefore only displaying part of the country's referral system. Referral data were based on reviews of health facility registers. Such data may be lacking or incomplete. However, it should be noted that the Ethiopian facility registers are job aids that assist the health extension worker to assess, classify, and treat the sick children. This fact may have enhanced completeness and thereby reduced reporting bias. The exclusion of records that were incomplete and poorly documented could also have led to reporting bias. However, only a few sick children records were incomplete and linked studies that observed the health extension workers' clinical competency and adherence to guidelines found similar referral adherence trends as shown in our study (23). We used objectively measured data on the health posts, based on the WHO health service availability and readiness assessment tool, and information obtained from the surveyed health posts were compared with national guidelines on referral. The study was performed in four of the largest regions in Ethiopia accounting for 80% of the population, where the enumeration areas were selected to represent the chosen districts of these regions. Though generalization of the study findings to the regions might be difficult, it is expected that the selected districts did not differ in health systems characteristics.

Despite the World Health Organization's recommendation (24), our study showed that only a third of young infants and half of the older children, who required referral, were referred from health post to the health

For peer review only

centres. This finding was consistent with the previous Ethiopian and Malawian studies (9, 25). The low referrals seen might be attributed to health providers' fear that caregivers would protest a referral (26), and the actual refusal of caregivers to comply with the referral (27). These factors might have ledthe health extension workers to manage the child at the health post (28). Health care providers might have also undermined the overall impact of treatment guidelines on childhood survival (26, 29). They may have perceived that there is little added value in referral and even felt a loss of power and prestige when referring (30). They might also have lacked the knowledge and have been reluctant to adhere to the guidelines (30). This low level of referral of severely sick children is concerning. It could delay access to appropriate care in the higher-level referral facilities, thus increasing the risk of death (31), and hinder the efforts to reach the sustainable development goals on neonatal and under-five mortality (32).

In this study, the severity of a child's illness was associated with their referral in both age groups (33). This relation indicates that severely ill children were more frequently managed according to the referral guidelines. There is a need to strengthen health extension workers' ability to identify severely sick children requiring urgent referral (34). Neonates were primarily referred in their first week of life, highlighting the need for urgent referrals of severely ill newborns to prevent death in neonatal sepsis and other critical conditions (35). Also, we found that distance from health posts to health centre was not associated with referrals, perhaps since nearly three-quarters of the health posts were located within 15 kilometres from their referral health centre.

Lack of the overall readiness of the health posts to provide the required childcare services is an important finding of this study. This scenario could indicate that many care-seeking sick children were not getting the care they required, at least not at the health post (23). The lack of essential medicines might have been a reason for some referrals of children with non-severe diseases, which otherwise could have been treated at the first-level health facility (33,36). Referral of children with non-severe illnesses could also impose unnecessary social and economic costs for caregivers (36,37).

For peer review only

Indeed, our data also showed that health posts that had medicine and diagnostics were likely to adhere to referral guidelines. This indicates the need to equip the health facilities with the basic essential medicines and diagnostics. Although not statistically significant in the multivariable analysis, health posts with two or more health extension works were also more likely to refer as well as adhere to referral guidelines for young infants. A study in Niger also found adequate staffing to be linked to adherence to referral guidelines (30).

The non-adherence of the some of the healthcare providers to the referral guidelines when managing severely sick children found in this study requires attention. This could result in overuse of antibiotics, and missing treatment or increased lethality in severe cases (38). Hence, mechanisms that can improve adherence to referral guidelines are needed as part of actions for improved quality-of-care.

Conclusion

We have shown that health extension workers referred few children with severe illnesses while a referral to the next-level health centre was recommended by guidelines. A significant proportion of the health posts were not ready to provide the necessary care, including referral. The severity of the child's illness, the availability of essential medicines and rapid diagnostic test were among the predictors of referrals of sick children from the health posts. Among other actions to enhance the quality of care provided at the health posts, improving medicine and diagnostic supplies may strengthen adherence to referral guidelines and ultimately prevent child deaths in Ethiopia.

Author affiliations

[1] Southern Nations, Nationalities & Peoples Regional Health Bureau, Department of maternal, newborn, and child health, and nutrition, Hawassa, Ethiopia

[2] Hawassa University, College of Medicine and Health Sciences, School of Public Health, Hawassa, Ethiopia

[3] Addis Ababa University, College of Health Sciences, Department of Paediatrics and Child Health, Addis Ababa, Ethiopia

For peer review only

[4] London School of Hygiene and Tropical Medicine, London, UK

[5] Ethiopian Public Health Institute, Addis Ababa 5654, Ethiopia

[6] Department of Epidemiology and Biostatistics, Institute of Public Health, College of Medicine and Health Sciences, University of Gondar, Gondar, Ethiopia

Acknowledgments

We would like to extend our sincere gratitude to SNNP Regional Health Bureau and Hawassa University, College of Medicine and Health Sciences for providing support to conduct this research project. We would also like to pass our deepest appreciation to the different levels of the health systems and health providers involved in this study.

Authors' contributions

HB contributed to the conceptualization of the study, design, development of the survey instrument, training and supervision of data collectors, statistical analysis, and wrote the original draft and final versions of the manuscript. DK and HT contributed to the conceptualization of the study design, reviewed and contributed to the different versions of the manuscript. DB and LÅP contributed to the conceptualization of the study, design, development of the survey instrument, data compilation and statistical analysis, and reviewed the different versions of the paper. AD contributed to the management of distance from health post to health centre data. All authors read and approved the final version of the manuscript.

Funding: The study was supported by a grant from Bill and Melinda Gates Foundation to The London School of Hygiene and Tropical Medicine (grant OPP1132551). The funder had no role in data collection, analysis, or interpretation of results.

Disclaimer: Not required.

Competing interests: The authors declared no competing interests exist.

For peer review only

Patient consent for publication: Not required.

Ethics approval

Ethical approvals were secured from the ethical review boards of Hawassa University (protocol number IRB/199/10; June 2018), the Ethiopian Public Health Institute (protocol number SERO-012-8-2016; Version 001, August 2016) and the London School of Hygiene & Tropical Medicine (protocol number 11235; June 2016).

Data availability statement: Data from this study are co-owned by the participating institutions and stored in a depository at the Ethiopian Public Health Institute (EPHI). The use of these data is guided by a data sharing agreement. Data can be accessed from the secretary of the data sharing committee of EPHI-LSHTM collaborative projects. Contact information: Martha Zeweldemariam, E-mail: martha.zeweldemariam@lshtm.ac.uk

ORCID IDs

Habtamu Beyene https://orcid.org/0000-0003-0923-5191

Della Berhanu https://orcid.org/0000-0002-4984-893X

Atkure Defar http:// orcid.org/ 0000-0001-9435-2135

Lars Åke Persson https://orcid.org/0000-0003-0710-7954

For peer review only

References

1. United Nations Inter-agency Group for Child Mortality Estimation (UNIGME), 'Levels & Trends in Child Mortality: Report 2019, Estimates developed by the United Nations Inter-agency Group for Child Mortality Estimation', United Nations Children's Fund, New York, 2019.
2. Ruducha J, Mann C, Singh NS, et al. Articles How Ethiopia achieved Millennium Development Goal 4 through multisectoral interventions : a Countdown to 2015 case study. *Lancet Glob Heal.* 2015; 5 (11):e1142–51.
3. Ethiopian Public Health Institute (EPHI) [Ethiopia] and ICF. 2019. Ethiopia Mini Demographic and Health Survey 2019: Key Indicators. Rockville, Maryland, USA: EPHI and ICF. Available from: https://www.dhsprogram.com/pubs/pdf/PR120/PR120.pdf. Accessed on August 24, 2020.
4. Deribew A; Tessema GA; Deribe K; Melaku YA; Lakew Y; Amare AT; Abera SF; Mohammed M; Hiruye A; Teklay E; Misganaw A; Kassebaum N. Trends, causes, and risk factors of mortality among children under 5 in Ethiopia, 1990-2013: findings from the Global Burden of Disease Study 2013. *Popul Health Metr.* 2016; 14:42.
5. Mugeni, C., Levine, A. C., Munyaneza, R. M., Mulindahabi, E., Hannah, C., Habimana, J. P. …Ngabo, F. Nationwide implementation of integrated community case management of childhood illness in Rwanda. *Glob Heal Sci Pract.* 2014; *2*: 328–341.
6. Mathewos B, Owen H, Sitrin D, *et al*. Community-Based interventions for newborns in Ethiopia (COMBINE): cost-effectiveness analysis. *Health Policy Plan* 2017; 32:i21–32.
7. World Health Organisation. Standards for improving the quality of care for children and young adolescents in health facilities. Geneva: World Health Organization, 2018. CC BY-NC-SA 3.0 IGO.
8. Miller NP, Amouzou A, Tafesse M, Hazel E, Legesse H, Degefie T, et al. Integrated Community Case Management of Childhood Illness in Ethiopia : Implementation Strength and Quality of Care. *Am. J. Trop. Med. Hyg.* 2014; 91:424–34.

For peer review only

9. Amouzou A, Hazel E, Shaw B, Miller NP, Tafesse M, Mekonnen Y, et al. Effects of the integrated Community Case Management of Childhood Illness Strategy on Child Mortality in Ethiopia : A Cluster Randomized Trial. *Am J Trop Med Hyg.* 2016; 94:596–604.

10. Marsh DR, Hamer DH, Pagnoni F, Peterson S. Introduction to a Special Supplement : Evidence for the Implementation, Effects, and Impact of the Integrated Community Case Management Strategy to Treat Childhood Infection. *Am J Trop Med Hyg.* 2012; 87:2–5.

11. World Health Organization. Referral Systems - a summary of key processes to guide health services managers. Geneva: World Health Organization, 2013. Available at: https://www.who.int/management/facility/referral/en/. Accessed on August 10, 2020.

12. Price J, Willcox M, Kabudula CW, Herbst K, Hinton L, Kahn K, et al. Care pathways during a child's final illness in rural South Africa: Findings from a social autopsy study. *PLoS ONE* 2019; 14: e0224284.

13. Beyene H, Hailu D, Tadele H, Persson LÅ, Berhanu D. Insufficient referral practices of sick children in Ethiopia based on a cross-sectional survey. *Acta Paediatr.* 2020*; 00:1–8.*

14. Abrahim O, Linnander E, Mohammed H, Fetene N, Bradley E. A Patient-Centered Understanding of the Referral System in Ethiopian Primary Health Care Units. *PLoS One*. 2015; 10:1–10.

15. Eskandari M, Abbaszadeh A, Borhani F. Barriers of Referral System to Health Care Provision in Rural Societies in Iran. *J Caring Sci.* 2013; 2:229–36.

16. Akbari A, Mayhew A, Al-alawi MA, Grimshaw J, Winkens R, Glidewell E, et al. Interventions to improve outpatient referrals from primary care to secondary care. *Cochrane Database Syst Rev.* 2014; 4: 1-50.

17. Berhanu D, Okwaraji YB, Belayneh AB, *et al*. Protocol for the evaluation of a complex intervention aiming at increased utilisation of primary child health services in Ethiopia: a before and after study in intervention and comparison areas. *BMC Health Serv Res.* 2020; 20:339.

18. Global financing facility. Ethiopia Health Sector Transformation Plan (2015/16-2019/20). https://www.globalfinancingfacility.org/ethiopia-health-sector-transformation-plan-201516-201920. Accessed on November 22, 2020.

For peer review only

19. FMOH. Federal Ministry of Health, Ethiopia. *Integrated Management of Newborn and Childhood Illness, Part 1*. Blended Learning Module for the Health Extension Programme. 2011. Retrieved from https://www.open.edu/openlearncreate/-pluginfile.php/71990/-mod_resource/content/2/IMNCI-_Part_1_Final_Print-ready_March_2011_.pdf. Accessed on November 15, 2020.

20. Berhanu D, Okwaraji YB, Defar A, et al. Does a complex intervention targeting communities, health facilities and district health managers increase the utilisation of community- based child health services? A before and after study in intervention and comparison areas of Ethiopia. *BMJ Open* 2020; 10:e040868.

21. Central Statistical Agency (CSA) [Ethiopia] and ICF (2016). Ethiopia: Demographic and Health Survey 2016 [Internet]. 2016. Available from: https://www.dhsprogram.com/publications/-publication-FR328-DHS-Final-Reports.cfm. Accessed on November 22, 2020.

22. World Health Organization. Service availability and readiness assessment (SARA). An annual monitoring system for service delivery. Reference manual. Geneva; 2015. Available from http://www.who.int/healthinfo/systems/sara_indicators_questionnaire/en/. Accessed 20 October, 2020.

23. Getachew T, Abebe SM, Yitayal M, Persson LÅ, Berhanu D. Assessing the quality of care in sick child services at health facilities in Ethiopia. *BMC Health Serv Res.* 2020; 7:1–12.

24. World Health Organization. Guideline: managing possible serious bacterial infection in young infants when referral is not feasible. World Health Organization. Geneva; 2015. https://apps.who.int/iris/-handle/10665/181426. Accessed 20 October, 2020.

25. Amouzou A, Kanyuka M, Hazel E, Heidkamp R, Marsh A, Mleme T, et al. Independent Evaluation of the integrated Community Case Management of Childhood Illness Strategy in Malawi Using a National Evaluation Platform Design. *Am J Trop Med Hyg.* 2016; 94:574–83.

26. Chinbuah MA, Abbey M, Kager PA, Gyapong M, Nonvignon J, Ashitey P, Akpakli J, Appiatse SA, Kubi D, Gyapong JO. Assessment of the adherence of community health workers to dosing and referral guidelines for the management of fever in children under 5 years: a study in Dangme West District, Ghana. *Int Health*. 2013; 5:148-56.

For peer review only

27. Hailegebriel TD, Mulligan B, Cousens S, Mathewos B, Wall S, Bekele A, et al. Effect on Neonatal Mortality of Newborn Infection Management at Health Posts When Referral Is Not Possible : *Glob Heal Sci Pract.* 2017; 5:202–16.

28. Wammanda RD, Adamu SA, Joshua HD, Nisar YB, Qazi SA, Aboubaker S, et al. Implementation of the WHO guideline on treatment of young infants with signs of possible serious bacterial infection when hospital referral is not feasible in rural Zaria, Nigeria: Challenges and solutions. *PLoS ONE*. 2020; 15: e0228718.

29. Wanduru P, Tetui M, Tuhebwe D, Ediau M, Okuga M, Nalwadda C, Ekirapa-Kiracho E, Waiswa P, Rutebemberwa E. The performance of community health workers in the management of multiple childhood infectious diseases in Lira, northern Uganda - a mixed methods cross-sectional study. *Glob Health Action.* 2016; 9:33194.

30. Bossyns P, Lerberghe W Van. Human Resources for Health The weakest link : competence and prestige as constraints to referral by isolated nurses in rural Niger. *BioMed Cent.* 2004; 8:1–8.

31. Nabongo P, Verver S, Nangobi E, Mutunzi R, Wajja A, Mayanja-kizza H, et al. Two year mortality and associated factors in a cohort of children from rural Uganda. *BMC Public Health.* 2014; 14:1–9.

32. Golding, N., Burstein, R., Longbottom, J., Browne, A. J., Fullman, N., Osgood-zimmerman, A., … Foundation, M. G. Mapping under-5 and neonatal mortality in Africa , 2000 – 15 : a baseline analysis for the Sustainable Development Goals. *Lancet*. 2015; *390*, 2171–2182.

33. Musa EO, Ejembi CL. Reasons and outcome of paediatric referrals from first- level health facilities in Sabongari , Zaria , Northwestern. *J Community Med Prim Heal Care.* 2004; 16:10–5.

34. Newbrander W, Ickx P, Werner R, Mujadidi F. Compliance with referral of sick children : a survey in five districts of Afghanistan. *BMC Pediatr.* 2012; 12:1471–2431.

35. Rosenstock S, Katz J, Mullany LC, Khatry SK, Leclerq SC, Darmstadt GL, et al. Sex differences in morbidity and care-seeking during the neonatal period in rural southern Nepal. *J Heal Popul Nutr*. 2015; 33:1–10.

36. Logaraj, M. Management of Referral Services through Computer Based Management System, *Nat.J.Res.Com.Med.* 2013; *2*, 1–4.

For peer review only

37. Nanyonjo A, Bagorogoza B, Kasteng F, Ayebale G, Makumbi F, Tomson G. Estimating the cost of referral and willingness to pay for referral to higher-level health facilities : a case series study from an integrated community case management programme in Uganda. *BMC Health Serv Res*. 2015;15:1–10.

38. Druetz T, Siekmans K, Goossens S. The community case management of pneumonia in Africa : a review of the evidence. Heal policy Planning. 2015;30(December 2013):253.

For peer review only

Legend to figure

Figure 1: Cumulative percentages of distance from health posts to the referral health centres, analysed based on linear distance from 158 health posts to their 128 referral health centres.

Figure 2. The mean percentage scores for five general service readiness domains of the surveyed 165 health posts analysed based on the WHO Service Availability and Readiness Assessment (SARA) reference manual (22).

Supplementary figure 1: Percentages of mean score of basic amenities by tracer items

Supplementary figure 2: Percentages of mean score of basic equipment by tracer items

Supplementary figure 3: Percentages of mean score of standard precaution for infection prevention by tracer items.

Supplementary figure 4: Percentages of mean score of essential medicines by tracer items.

For peer review only

List of tables

Table 1.Characteristics of health posts in four regions of Ethiopia (December 2018-February 2019) (n=165)

Characteristics		n	%
Region			
	Amhara	70	42
	Oromia	48	29
	SNNPR[a]	22	13
	Tigray	25	15
Number of health extension worker per health post			
	1	35	21
	2+	130	79
Availability of			
	Registers for 0 – 59 days old children	148	90
	Registers for 2 -59 months old children	156	95
	Cell phone signals in the health post	135	82
Number of health posts that referred sick children in the last quarter		69	42
Means of communication used for the referral of last sick child			
	Facility landline or mobile phone	3	2
	Staff member's mobile phone	25	15
	In person communication	13	8
	No communication for last child referral	124	75

[a]SNNPR - Southern Nations, Nationalities, and Peoples Region

For peer review only

Table 2. Disease classification, recommended and performed referrals among children 0-59 months old seen at the surveyed 165 health posts, December 2018 – February 2019.

Age group	Disease classification	Referral guideline supports referral?	Number seen	Number (%) referred
0 – 59 days	Very preterm or very low birth weight	Yes	0	0
	Preterm or low birth weight	Yes	4	2 (50)
	Possible serious bacterial infection	Yes	42	15 (36)
	Severe dehydration	Yes	0	0
	Severe persistent diarrhea	Yes	1	0
	Dysentery	Yes	1	1 (100)
	Severe jaundice	Yes	1	0
	Local bacterial infection	No	59	5 (9)
	Some dehydration	No	2	1 (50)
	No dehydration	No	17	1 (6)
	Jaundice	No	1	0
	Malaria	No	0	0
	Feeding problem or low weight	No	45	2 (4)
	Classification not given	-	26	0
	Other[a]	No	30	12 (40)
	Total 0 – 59 days old		229	39 (17)
2 – 59 moths	Severe pneumonia/very severe disease	Yes	9	8 (89)
	Severe dehydration	Yes	2	1 (50)
	Severe persistent diarrhea	Yes	3	1 (33)
	Dysentery	Yes	9	6 (67)
	Very severe febrile disease	Yes	2	1 (50)
	Severe complicated measles	Yes	0	0
	Severe malnutrition	Yes	22	6 (27)
	Severe anaemia	Yes	0	0
	Pneumonia	No	478	16 (3)
	Some dehydration	No	186	9 (5)
	No dehydration	No	203	0
	Persistent diarrhea	No	28	2 (7)
	Malaria	No	12	0
	Fever, malaria unlikely	No	11	0
	Fever, no malaria	No	55	4 (7)
	Measles with eye/mouth complications	No	0	0

For peer review only

Age group	Disease classification	Referral guideline supports referral?	Number seen	Number (%) referred
	Measles	No	1	1 (100)
	Acute ear infection	No	7	3 (43)
	Chronic ear infection	No	1	0
	Moderate malnutrition	No	33	4 (12)
	Anaemia	No	1	0
	Others[b]	No	64	16 (25)
	Total 2 – 59 months old		1123	78 (7)

[a]Other – classification for 0 – 59 days included "infection unlikely" (12), "eye problems" (10), and PNC cases with referral outcome (8)

[b]Others – classification for 2 – 59 months old children included mainly "no pneumonia, cough" (42), and "eye problems" (6), growth monitoring with referral outcomes (16).

For peer review only

Table 3. Factors associated with referral of 0-59 days old infants and 2-59 months old children from health post to higher levels in four Ethiopian regions, December 2018-February 2019. Logistic regression analyses.

Characteristic	Levels	Referred (n)	Not referred (n)	Crude OR (95% CI)	Adjusted OR (95% CI)[1]
**For 0-59 days old infants (N=228)[2]**					
The health post					
Facility owned landline or mobile phone	Not available	13	35	1.0	1.0
	Available	26	154	0.47 (0.17 – 1.28)	0.61 (0.13 – 2.80)
Mean score of essential medicine	0 to 33 percent	1	11	1.0	1.0
	34 to 66 percent	32	112	3.14 (0.99 - 9.99)	3.17 (0.42 - 24.19)
	67 to 100 percent	6	66	1.00 (0.24 – 4.14)	1.34 (0.10 – 18.80)
Number of health extension workers	1	2	39	1.0	1.0
	2 or more	37	150	4.81 (1.10 - 21.05)[+]	5.63 (0.87 – 36.40)
Distance between health post and the nearest referral health centre					
	0-8 km	14	60	1.0	1.0
Distance tertiles	8.01-20 km	11	63	0.54 (0.18 – 1.60)	0.60 (0.18 - 2.04)
	20.01 or more km	14	66	0.87 (0.26 – 2.96)	1.02 (0.33 – 3.17)
The child					
Age	0-1 week	14	45	1.0	1.0
	2-4 weeks	16	82	0.63 (0.21 – 1.84)	0.35 (0.11 – 1.10)
	5 or more weeks	9	62	0.47 (0.15 - 1.46)	0.25 (0.10 - 0.64)[++]
Gender	Boy	18	105	1.0	1.0
	Girl	21	84	1.46 (0.59 - 3.62)	1.12 (0.45 - 2.75)
Disease classification	Not severe	21	158	1.0	1.0
	Severe	18	31	4.37 (1.30 – 14.67)[+]	6.72 (1.50 - 30.11)[+]
**For 2-59 months old children (N=1074)[3]**					
The health post					
Facility owned landline or mobile phone	Not available	44	792	1.0	1.0
	Available	27	211	2.30 (1.16 – 4.55)[+]	1.88 (0.94 – 3.76)
Mean score of basic amenities	0 to 33 percent	20	129	1.0	1.0
	34 to 66 percent	15	279	0.35 (0.13 – 0.89)[+]	0.38 (0.15 – 0.96)[+]
	67 to 100 percent	36	595	0.39 (0.17 – 0.89)[+]	0.40 (0.16 - 1.03)
Mean score of basic equipment	0 to 33 percent	2	64	1.0	1.0
	34 to 66 percent	12	147	2.61 (0.65 - 10.45)	3.49 (0.16 - 75.12)
	67 to 100 percent	57	792	2.30 (0.62 - 8.53)	2.38 (0.12 - 48.96)
Amoxicillin	Not available	27	152	1.0	1.0
	Available	44	851	0.29 (0.15 – 0.58)[++]	0.41 (0.19 - 0.88)[+]

For peer review only

Characteristic	Levels	Referred (n)	Not referred (n)	Crude OR (95% CI)	Adjusted OR (95% CI)[1]
Rapid diagnostic test for malaria	Not available	45	449	1.0	1.0
	Available	26	554	0.47 (0.26 – 0.83)[+]	0.18 (0.07 - 0.46)[++]
Mean score of overall service readiness[3]	0 to 33 percent	6	22	1.0	1.0
	34 to 66 percent	43	573	0.28 (0.04 – 2.05)	0.21 (0.03 – 1.53)
	67 to 100 percent	22	408	0.20 (0.03 – 1.49)	0.62 (0.07 – 5.60)
Number of health extension workers	1	20	196	1.0	1.0
	2 or more	51	807	0.62 (0.30 – 1.28)	1.01 (0.38 - 2.65)
Distance between health post and the nearest referral health centre					
	0-8 km	24	321	1.0	1.0
Distance tertiles	8.01-14 km	25	320	1.18 (0.54 - 2.56)	0.98 (0.43 – 2.21)
	14.01 or more km	22	362	0.86 (0.43 - 1.71)	0.71 (0.28 - 1.79)
The child					
Age	0-1 week	25	284	1.0	1.0
	2-4 weeks	24	317	0.86 (0.48 - 1.54)	0.97 (0.52 - 1.81)
	5 or more weeks	22	402	0.62 (0.36 - 1.07)	0.88 (0.45 - 1.68)
Gender	Boy	35	526	1.0	1.0
	Girl	36	477	1.13 (0.71 -1.81)	0.89 (0.52 - 1.52)
Disease classification	Not severe	50	981	1.0	1.0
	Severe	21	22	18.73 (9.21 – 38.09)[+++]	21.27 (7.51 - 60.23)[+++]

[1] Multivariable logistic regression adjusted for region and clusters. The logistic regression model was tested using the Hosmer and Lemeshow test of the goodness-of-fit statistics, which suggested that the model was a good fit to the data.

[2]Records of 1 sick young infant of 0-59 days old were removed because of missing of data of distance from health post to the referral health centre.

[3]Records of 49 sick older children of 2-59 months old were removed because of missing of data of distance from health post to the referral health centre.

[+++] P-value < 0.001 [++] P-value < 0.01 [+] P-value < 0.05

For peer review only

List of Supplementary tables

Supplementary table 1: Health post and child factors associated with referrals of 0 – 59 months sick young children in four regions of Ethiopia

Characteristics	Referral status of 0 – 59 days old sick young infants			Referral status of 2 – 59 months old children		
	Referred N=39 n (%)	Not referred N=190 n (%)	P-value	Referred N=78 n (%)	Not referred N=1045 n (%)	P-value
The health post						
Mean score of basic amenities						
0 to 33 percent	13 (24)	42 (76)	0.466	20 (13)	129 (87)	0.043
34 to 66 percent	9 (15)	50 (85)		19 (6)	292 (94)	
67 to 100 percent	17 (15)	98 (85)		39 (6)	624 (94)	
Mean score of basic equipment						
0 to 33 percent	2 (22)	7 (78)	0.825	3 (4)	73 (96)	0.512
34 to 66 percent	10 (19)	43 (81)		12 (8)	147 (92)	
67 to 100 percent	27 (16)	140 (84)		63 (7)	825 (93)	
Mean score of standard precaution						
0 to 33 percent	4 (9)	40 (91)	0.527	30 (8)	353 (92)	0.279
34 to 66 percent	14 (19)	59 (81)		31 (8)	355 (92)	
67 to 100 percent	21 (19)	91 (81)		17 (5)	337 (95)	
Rapid diagnostic test (RDT)						
0 to 33 percent	10 (17)	50 (83)	0.947	47 (9)	467 (91)	0.026
34 to 66 percent	0	0		0	0	
67 to 100 percent	29 (17)	140 (83)		31 (5)	578 (95)	
Mean score of essential medicines						
0 to 33 percent	1 (8)	11 (92)	0.026	17 (18)	77 (82)	0.001
34 to 66 percent	32 (21)	113 (79)		43 (6)	697 (94)	
67 to 100 percent	6 (8)	66 (92)		18 (6)	271 (94)	
Health post's overall service readiness index						
0 to 33 percent	0 (0)	1 (0)	0.737	7 (18)	31 (82)	0.193
34 to 66 percent	22 (19)	93 (81)		43 (7)	575 (93)	
67 to 100 percent	17 (15)	96 (85)		28 (6)	439 (94)	
Number of health extension worker per health post						
1	2 (5)	39 (95)	0.024	20 (9)	196 (81)	0.264
2 or more	37 (20)	151 (80)		58 (6)	849 (94)	
Distance between health post and the nearest referral health centre						

For peer review only

Distance to the next referral site[b]						
0 - 8 kms	16 (21)	61 (79)				
8.01 - 20 kms	10 (12)	71 (88)	0.554			
>20 kms	13 (19)	57 (81)				
0 - 8 kms				24 (7)	340 (93)	
8.01 - 14 kms				25 (8)	301 (92)	0.699
>14 kms				22 (6)	362 (94)	
The child						
Region						
Amhara	3 (25)	9 (75)		34 (7)	428 (93)	
Oromia	10 (13)	65 (87)	0.722	26 (7)	342 (93)	0.691
SNNPR[a]	9 (21)	33 (79)		11 (8)	128 (92)	
Tigray	17 (17)	83 (83)		7 (5)	147 (95)	
Age						
1st week	14 (24)	45 (76)				
2 – 4 weeks	16 (16)	82 (84)	0.353			
5 – 8 weeks	9 (13)	63 (87)				
2 – 11 months				26 (8)	294 (92)	
12 – 23 months				28 (8)	325 (92)	0.249
24 – 59 months				24 (5)	426 (95)	
Sex						
Boy	18 (15)	106 (85)	0.395	40 (7)	550 (93)	0.810
Girl	21 (20)	84 (80)		38 (7)	495 (93)	
Disease classification						
Non-severe illnesses not requiring referral	21 (12)	159 (88)	0.013	56 (5)	1023 (95)	0.001
Severe illnesses requiring referral	18 (37)	31 (63)		22 (50)	22 (50)	

[a] SNNPR - Southern Nations, Nationalities, and Peoples Region

[b] GIS based data of distance from health post to health centre were missing for 0-59 days sick young infant (1) and 2-59 months sick children (49).

For peer review only

Supplementary table 2: Individual health post readiness factors associated with referrals of 0-59 months sick children in four regions of Ethiopia

Characteristics		Referral status of 0 – 59 days sick young infants			Referral status of 2 – 59 months old children		
		Referred N=39 n (%)	Not referred N=190 n (%)	P-value	Referred N=78 n (%)	Referred N=1045 n (%)	P-value
Availability of basic amenities							
Water source							
	Not available	15 (22)	53 (78)	0.296	30 (8)	356 (92)	0.558
	Available	24 (15)	137 (85)		48 (7)	689 (93)	
Electric power							
	Not available	18 (17)	91 (83)	0.886	51 (8)	602 (92)	0.257
	Available	21 (18)	99 (82)		27 (6)	443 (94)	
Facility owned toilet							
	Not available	6 (18)	27 (82)	0.880	14 (7)	207 (93)	0.981
	Available	33 (17)	163 (83)		64 (7)	838 (93)	
Facility owned landline or mobile phone							
	Not available	35 (19)	153 (81)	0.133	51 (6)	828 (94)	0.038
	Available	4 (10)	37 (90)		27 (11)	217 (89)	
Availability of basic equipment							
Thermometer							
	Not available	8 (14)	49 (86)	0.594	12 (5)	228 (95)	0.207
	Available	31 (18)	141 (82)		66 (8)	817 (92)	
Child weight scale							
	Not available	5 (13)	34 (87)	0.526	20 (9)	214 (91)	0.388
	Available	34 (18)	156 (82)		58 (7)	831 (93)	
Stethoscope							
	Not available	18 (19)	79 (81)	0.709	35 (8)	399 (92)	0.336
	Available	21 (16)	111 (84)		43 (6)	646 (94)	
MUAC tape measure							
	Not available	1 (100)	0	0.040	0 (0)	14 (100)	0.701
	Available	38 (17)	190 (83)		78 (7)	1031 (93)	
Availability diagnostics							
Rapid diagnostic test (RDT)							
	Not available	10 (17)	50 (84)	0.947	47 (9)	467 (91)	0.026
	Available	29 (17)	140 (82)		31 (5)	578 (95)	
Availability of essential medicines							
Vitamin A							

For peer review only

	Not available	9 (27)	25 (73)	0.211	11 (5)	207 (95)	0.214
	Available	30 (15)	165 (85)		67 (7)	838 (93)	
Amoxicillin							
	Not available	6 (14)	36 (86)	0.745	28 (15)	159 (85)	0.001
	Available	33 (18)	154 (82)		50 (5)	886 (95)	
Gentamycin							
	Not available	20 (14)	127 (86)	0.201			
	Available	19 (23)	63 (77)				
Tetracycline eye ointment							
	Not available	26 (19)	110 (81)	0.457	46 (8)	567 (92)	0.554
	Available	13 (14)	80 (86)		32 (6)	478 (94)	
Oral rehydration solution (ORS)							
	Not available	2 (13)	13 (87)	0.627	10 (17)	48 (83)	0.048
	Available	37 (17)	177 (83)		68 (6)	997 (94)	
Zinc-ORS							
	Not available				69 (7)	947 (93)	0.608
	Available				9 (8)	98 (92)	
Zinc							
	Not available				16 (9)	152 (91)	0.331
	Available				62 (7)	893 (93)	
Coartem							
	Not available	18 (20)	71 (80)	0.494	47 (9)	507 (91)	0.087
	Available	21 (15)	119 (85)		31 (5)	535 (95)	
Plumpy Nut							
	Not available	8 (23)	27 (77)	0.404	30 (8)	372 (92)	0.688
	Available	31 (16)	163 (84)		48 (7)	673 (93)	
BP100							
	Not available	38 (17)	180 (83)	0.012	77 (7)	993 (93)	0.107
	Available	1 (9)	10 (91)		1 (2)	52 (98)	

For peer review only

For peer review only - http://bmjopen.bmj.com/site/about/guidelines.xhtml

Supplementary table 3. Adherence to referral management guidelines for sick young infants and older children

Age group	Observed	Management guideline	
		Referral advised	Referral not advised
0-59 days	Referred	18	21
	Not referred	31	159
2-59 months	Referred	22	56
	Not referred	22	1023

*Shadowed cells indicate referral management that did not adhere to guidelines

For peer review only

Supplementary table 4: Logistic regression for correct management of 0-59 days old sick young infants according to guidelines

Characteristic	Levels	Management according to guideline		Crude OR (95% CI)	Adjusted OR (95% CI)
		Correct (n)	Not correct (n)		
Facility-owned landline or mobile phone	Not available	145	42	1.0	1.0
	Available	31	10	0.90 (0.26 – 3.07)	1.12 (0.27 – 4.68)
Mean score of essential medicine	0 to 33 percent	7	5	1.0	1.0
	34 to 66 percent	112	32	2.50 (0.83 – 7.51)	4.49 (0.93 – 21.77)
	67 to 100 percent	57	15	2.71 (0.51 – 14.46)	12.95 (1.49 – 112.82)$^{+}$
Number of health extension workers	1	27	14	1.0	1.0
	2 or more	149	38	2.03 (0.37 – 11.28)	8.51 (0.83 – 86.98)
Distance tertiles	0-8 km	54	23	1.0	1.0
	8.01-20 km	63	18	1.49 (0.37 – 6.03)	1.01 (0.17 – 6.13)
	20.01 or more km	59	11	2.28 (0.56 – 9.30)	0.58 (0.08 – 4.20)
Age	0-1 week	49	10	1.0	1.0
	2-4 weeks	69	29	0.49 (0.15 – 1.58)	0.57 (0.15 – 2.24)
	5 or more weeks	58	13	0.91 (0.26 – 3.23)	1.37 (0.28 - 6.76)
Gender	Boy	95	28	1.0	1.0
	Girl	81	24	0.99 (0.47 – 2.11)	1.19 (0.55 - 2.58)

$^{+++}$ P-value < 0.001 $^{++}$ P-value < 0.01 $^{+}$ P-value < 0.05

For peer review only

Supplementary table 5: Logistic regression for correct management of 2-59 months old sick children according to guidelines

Characteristic	Levels	Management according to guideline		Crude OR (95% CI)	Adjusted OR (95% CI)
		Correct (n)	Not correct (n)		
Age	0-1 week	282	27	1.0	1.0
	2-4 weeks	315	26	1.16 (0.65 – 2.06)	1.24 (0.70 – 2.18)
	5 or more weeks	402	22	1.75 (0.95 – 3.23)	1.78 (0.93 - 3.40)
Gender	Boy	524	37	1.0	1.0
	Girl	475	38	0.88 (0.56 – 1.39)	0.97 (0.61 - 1.55)
Amoxicillin	Not available	153	26	1.0	1.0
	Available	846	49	2.93 (1.48 – 5.82)++	2.74 (1.40 – 5.35)++
Distance tertiles	0-8 km	340	24	1.0	1.0
	8.01-14 km	298	28	0.75 (0.36 – 1.58)	0.72 (0.28 – 1.82)
	14.01 or more km	361	23	1.11 (0.54 – 2.27)	0.96 (0.44 – 2.05)
Mean score of basic amenities	0 to 33 percent	130	19	1.0	1.0
	34 to 66 percent	278	16	2.54 (1.01 – 6.39)+	1.97 (0.71 – 5.49)
	67 to 100 percent	591	40	2.16 (0.93 – 5.02)	1.41 (0.56 – 3.56)
Mean score of basic equipment	0 to 33 percent	61	5	1.0	1.0
	34 to 66 percent	141	18	0.64 (0.21 – 1.93)	0.96 (0.12 – 7.57)
	67 to 100 percent	797	52	1.26 (0.45 – 3.47)	2.46 (0.29 – 20.54)
Mean score of diagnostics	0 to 33 percent	448	46	1.0	1.0
	67 to 100 percent	551	29	1.95 (1.10 – 3.46)+	4.59 (1.79 - 11.75)++
Mean score of overall readiness	0 to 33 percent	21	7	1.0	1.0
	34 to 66 percent	570	46	4.13 (0.68 – 25.02)	3.19 (0.43 - 23.61)
	67 to 100 percent	408	22	6.18 (1.01 – 37.87)	1.59 (0.14 – 18.00)
Number of health extension workers	1	193	23	1.0	1.0
	2 or more	806	52	1.84 (0.92 – 3.72)	1.52 (0.77 – 3.02)

+++ P-value < 0.001 ++ P-value < 0.01 + P-value < 0. 0.05

For peer review only

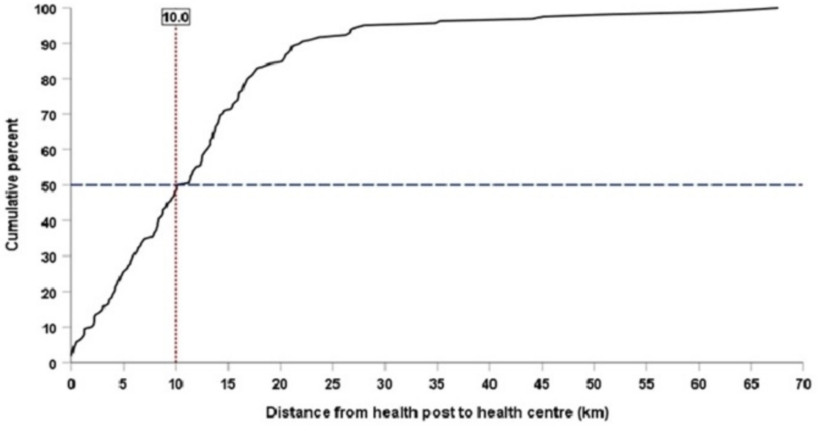

216x121mm (96 x 96 DPI)

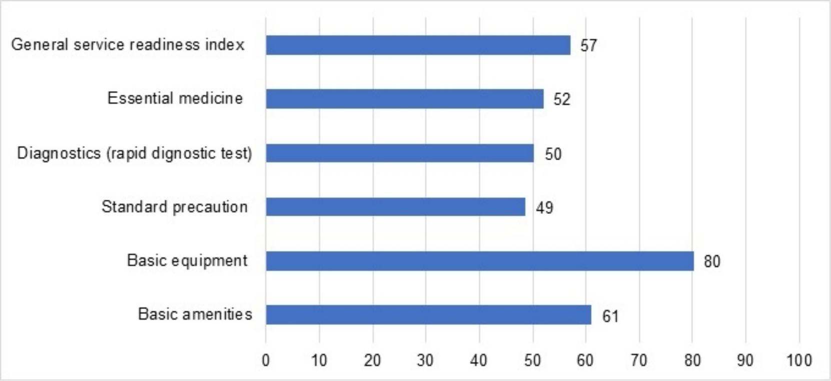

216x121mm (96 x 96 DPI)

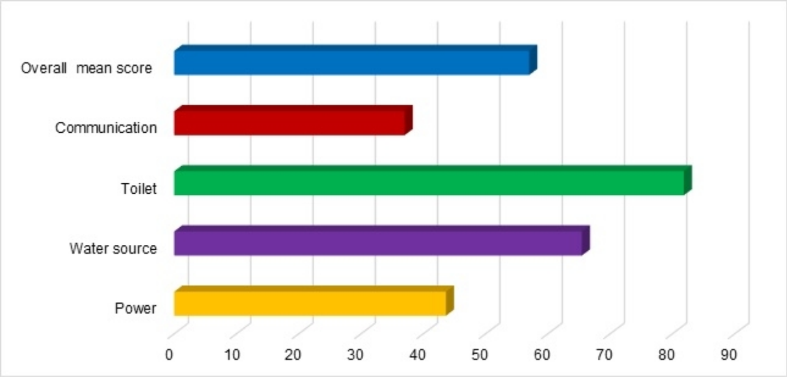

216x121mm (96 x 96 DPI)

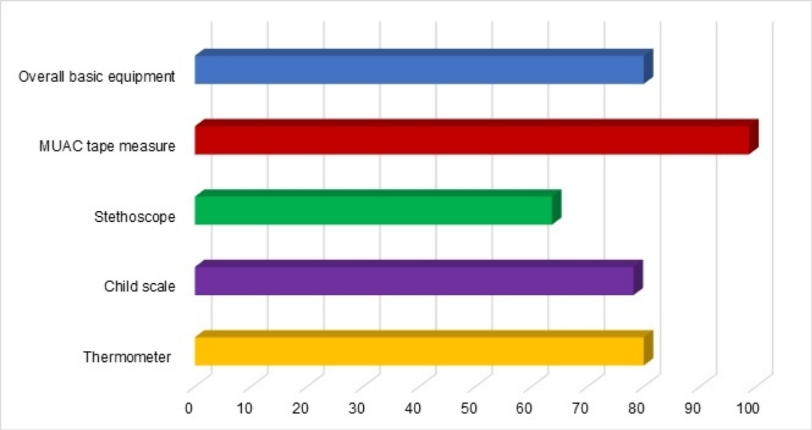

216x121mm (96 x 96 DPI)

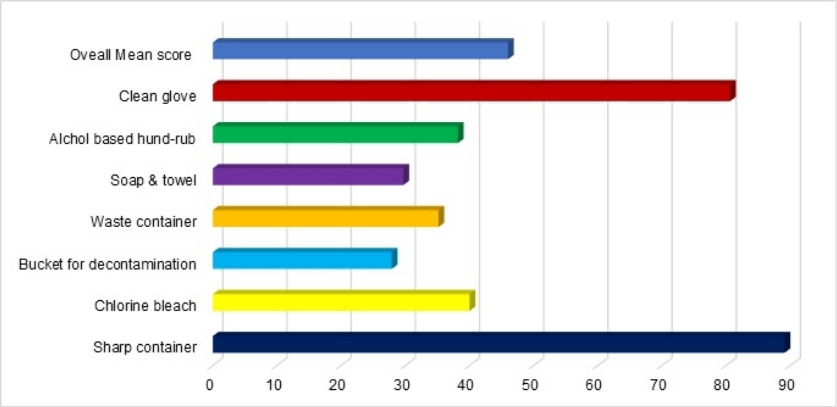

216x121mm (96 x 96 DPI)

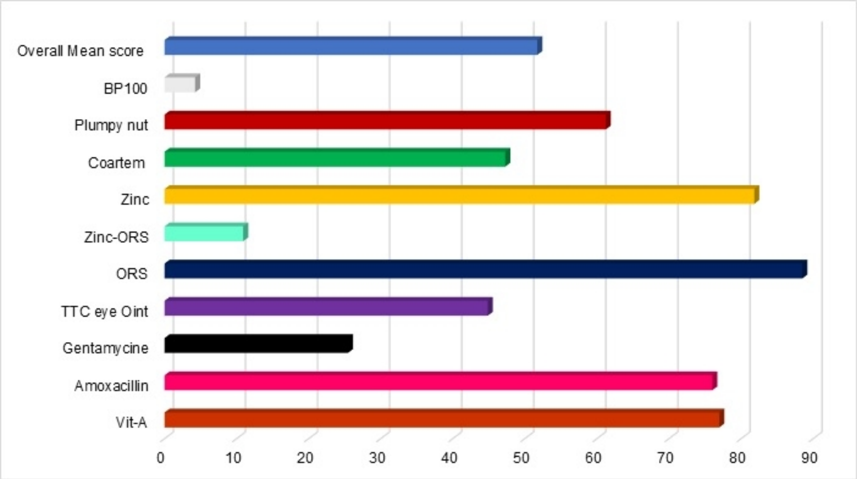

216x121mm (96 x 96 DPI)

STROBE 2007 (v4) Statement—Checklist of items that should be included in reports of *cross-sectional studies*

Section/Topic	Item #	Recommendation	Reported on page #
**Title and abstract**	1	(*a*) Indicate the study's design with a commonly used term in the title or the abstract	1
		(*b*) Provide in the abstract an informative and balanced summary of what was done and what was found	2
**Introduction**			
Background/rationale	2	Explain the scientific background and rationale for the investigation being reported	3
Objectives	3	State specific objectives, including any prespecified hypotheses	5
**Methods**			
Study design	4	Present key elements of study design early in the paper	5
Setting	5	Describe the setting, locations, and relevant dates, including periods of recruitment, exposure, follow-up, and data collection	5
Participants	6	(*a*) Give the eligibility criteria, and the sources and methods of selection of participants	6
Variables	7	Clearly define all outcomes, exposures, predictors, potential confounders, and effect modifiers. Give diagnostic criteria, if applicable	8
Data sources/ measurement	8*	For each variable of interest, give sources of data and details of methods of assessment (measurement). Describe comparability of assessment methods if there is more than one group	7
Bias	9	Describe any efforts to address potential sources of bias	8
Study size	10	Explain how the study size was arrived at	6 – 7
Quantitative variables	11	Explain how quantitative variables were handled in the analyses. If applicable, describe which groupings were chosen and why	7 – 8
Statistical methods	12	(*a*) Describe all statistical methods, including those used to control for confounding	8
		(*b*) Describe any methods used to examine subgroups and interactions	8 - 9
		(*c*) Explain how missing data were addressed	29 - 30
		(*d*) If applicable, describe analytical methods taking account of sampling strategy	8
		(*e*) Describe any sensitivity analyses	-
**Results**			

For peer review only

Participants	13*	(a) Report numbers of individuals at each stage of study—eg numbers potentially eligible, examined for eligibility, confirmed eligible, included in the study, completing follow-up, and analysed	9
		(b) Give reasons for non-participation at each stage	-
		(c) Consider use of a flow diagram	-
Descriptive data	14*	(a) Give characteristics of study participants (eg demographic, clinical, social) and information on exposures and potential confounders	9
		(b) Indicate number of participants with missing data for each variable of interest	8, 28, 29, 30
Outcome data	15*	Report numbers of outcome events or summary measures	10, 25
Main results	16	(*a*) Give unadjusted estimates and, if applicable, confounder-adjusted estimates and their precision (eg, 95% confidence interval). Make clear which confounders were adjusted for and why they were included	11, 29, 30
		(*b*) Report category boundaries when continuous variables were categorized	10, 29, 30
		(*c*) If relevant, consider translating estimates of relative risk into absolute risk for a meaningful time period	N/A
Other analyses	17	Report other analyses done—eg analyses of subgroups and interactions, and sensitivity analyses	12
**Discussion**			
Key results	18	Summarise key results with reference to study objectives	12
Limitations	19	Discuss limitations of the study, taking into account sources of potential bias or imprecision. Discuss both direction and magnitude of any potential bias	12 - 13
Interpretation	20	Give a cautious overall interpretation of results considering objectives, limitations, multiplicity of analyses, results from similar studies, and other relevant evidence	13 - 14
Generalisability	21	Discuss the generalisability (external validity) of the study results	13
**Other information**			
Funding	22	Give the source of funding and the role of the funders for the present study and, if applicable, for the original study on which the present article is based	16

*Give information separately for cases and controls in case-control studies and, if applicable, for exposed and unexposed groups in cohort and cross-sectional studies.

Note: An Explanation and Elaboration article discusses each checklist item and gives methodological background and published examples of transparent reporting. The STROBE checklist is best used in conjunction with this article (freely available on the Web sites of PLoS Medicine at http://www.plosmedicine.org/, Annals of Internal Medicine at http://www.annals.org/, and Epidemiology at http://www.epidem.com/). Information on the STROBE Initiative is available at www.strobe-statement.org.

For peer review only

BMJ Open

Factors associated with the referral of children with severe illnesses at primary care level in Ethiopia: a cross-sectional study.

Journal:	*BMJ Open*
Manuscript ID	bmjopen-2020-047640.R2
Article Type:	Original research
Date Submitted by the Author:	20-May-2021
Complete List of Authors:	Beyene, Habtamu; Hawassa University, College of Medicine and Health Sciences; Southern Nations Nationalities and Peoples' Region, Regional Health Bureau<br>Kassa, Dejene; Hawassa University, College of Medicine and Health Sciences, School of Public Health<br>Tadele, Henok; Addis Ababa University, College of Health Sciences, Department of Paediatrics and Child Health<br>Persson, Lars; London School of Hygiene and Tropical Medicine Faculty of Infectious and Tropical Diseases; Ethiopian Public Health Institute<br>Defar, Atkure ; Ethiopian Public Health Institute, Health System and Reproductive Health Research Directorate ; University of Gondar, Epidemiology and Biostatistics, Institute of Public Health, College of Medicine and Health Sciences<br>Berhanu, Della; London School of Hygiene and Tropical Medicine Faculty of Infectious and Tropical Diseases; Ethiopian Public Health Institute
**Primary Subject Heading**:	Paediatrics
Secondary Subject Heading:	Public health
Keywords:	Organisation of health services < HEALTH SERVICES ADMINISTRATION & MANAGEMENT, Quality in health care < HEALTH SERVICES ADMINISTRATION & MANAGEMENT, Community child health < PAEDIATRICS, EPIDEMIOLOGY

SCHOLARONE™
Manuscripts

I, the Submitting Author has the right to grant and does grant on behalf of all authors of the Work (as defined in the below author licence), an exclusive licence and/or a non-exclusive licence for contributions from authors who are: i) UK Crown employees; ii) where BMJ has agreed a CC-BY licence shall apply, and/or iii) in accordance with the terms applicable for US Federal Government officers or employees acting as part of their official duties; on a worldwide, perpetual, irrevocable, royalty-free basis to BMJ Publishing Group Ltd ("BMJ") its licensees and where the relevant Journal is co-owned by BMJ to the co-owners of the Journal, to publish the Work in this journal and any other BMJ products and to exploit all rights, as set out in our licence.

The Submitting Author accepts and understands that any supply made under these terms is made by BMJ to the Submitting Author unless you are acting as an employee on behalf of your employer or a postgraduate student of an affiliated institution which is paying any applicable article publishing charge ("APC") for Open Access articles. Where the Submitting Author wishes to make the Work available on an Open Access basis (and intends to pay the relevant APC), the terms of reuse of such Open Access shall be governed by a Creative Commons licence – details of these licences and which Creative Commons licence will apply to this Work are set out in our licence referred to above.

Other than as permitted in any relevant BMJ Author's Self Archiving Policies, I confirm this Work has not been accepted for publication elsewhere, is not being considered for publication elsewhere and does not duplicate material already published. I confirm all authors consent to publication of this Work and authorise the granting of this licence.

For peer review only

Factors associated with the referral of children with severe illnesses at primary care level in Ethiopia: a cross-sectional study.

Short run title: Referral of sick children in Ethiopia

Habtamu Beyene[1, 2], Dejene Hailu[2], Henok Tadele[3], Lars Åke Persson[4, 5], Atkure Defar[5, 6], Della Berhanu[4, 5]

Corresponding author:

Habtamu Beyene; MSc, BSc

Tel: +251-911-003346

Email: habtamu3346@yahoo.com

Author affiliations

[1] Southern Nations, Nationalities & Peoples Regional Health Bureau, Department of maternal, newborn, and child health, and nutrition, Hawassa, Ethiopia

[2] Hawassa University, College of Medicine and Health Sciences, School of Public Health, Hawassa, Ethiopia

[3] Addis Ababa University, College of Health Sciences, Department of Paediatrics and Child Health, Addis Ababa, Ethiopia

[4] Department of Disease Control, Faculty of Infectious and Tropical Diseases, London School of Hygiene and Tropical Medicine, London, UK

[5]Health Systems and Reproductive Health Research Directorate, Ethiopian Public Health Institute, Addis Ababa, Ethiopia

[6] Department of Epidemiology and Biostatistics, Institute of Public Health, College of Medicine and Health Sciences, University of Gondar, Gondar, Ethiopia

For peer review only

ABSTRACT

Context and objective: Ethiopia's primary care has a weak referral system for sick children. We aimed to identify health post and child factors associated with referrals of sick children 0-59 months of age and evaluate the healthcare providers' adherence to referral guidelines.

Design: A cross-sectional facility-based survey.

Setting: This study included data from 165 health posts in 52 districts in four Ethiopian regions collected from December 2018 to February 2019. The data included interviews with health extension workers, assessment of health post preparedness, recording of GPS-coordinates of the health post and the referral health centre, and reviewing registers of sick children treated during the last three months at the health posts. We analysed the association between the sick child's characteristics, health post preparedness, and distance to the health centre with referral of sick children by multivariable logistic regressions.

Outcome measure: Referral to the nearest health centre of sick young infants aged 0-59 days and sick children 2-59 months.

Results: The health extension workers referred 39/229 (17%) of the sick young infants and 78/1123 (7%) of the older children to the next level of care. Only 18 (37%) sick young infants and 22 (50%) 2-59 months children that deserved urgent referral according to guidelines were referred. The leading causes of referral were possible serious bacterial infection and pneumonia. Those being classified as a severe disease were referred more frequently. The availability of basic amenities (AOR = 0.38, 95% CI: 0.15 – 0.96), amoxicillin (AOR = 0.41, 95% CI: 0.19 - 0.88) and rapid diagnostic test (AOR = 0.18, 95% CI: 0.07 - 0.46) were associated with less referral in the older age group.

Conclusion: Few children with severe illness were referred from health posts to health centres. Improving the health posts' medicine and diagnostic supplies may enhance adherence to referral guidelines and ultimately reduce child mortality.

Key words: health extension workers, referral, severe illnesses, sick child, referral factors.

For peer review only

Strengths and limitations of this study

- Based on secondary analysis of register data from 165 health posts in 52 districts of the four most populous regions of Ethiopia, this study reports primary care referral patterns and associated factors in rural areas.
- This study used objectively measured data based on the WHO health service availability and readiness assessment tool and information obtained from the surveyed health posts compared with the national guidelines on referral.
- Using a cross-sectional study design, we cannot prove any causal relationship between the health post readiness, child factors, and referral of sick children observed in this study.
- As the study was mainly based on register reviews, incomplete documentation of some of the health facilities might lead to reporting bias.
- The study findings do not give a complete picture of the country's referral system as we limited the analysis to the primary healthcare level, and we did not collect data from parents of the referred children.

For peer review only

INTRODUCTION

Globally, nearly 5.3 million children below the age of 5 years died in 2018. Of these deaths, 47% occurred during the first month of life. More than half of this mortality occurred in Sub-Saharan Africa. Infectious diseases remained the leading causes of death (1).

Ethiopia achieved a significant reduction in child mortality during the Millennium Development Goals era from 1990 to 2015 (2). However, under-five and neonatal mortality rates remain high with 55 and 30 deaths per 1,000 live births, respectively (3). Lower respiratory tract infection, diarrhoeal diseases, and neonatal causes (mainly preterm birth complications, asphyxia, and neonatal sepsis), were the leading causes of death (4).

The integrated case management of childhood illnesses and the community based newborn care programmes are, if delivered with quality, cost-effective in reducing under-five deaths (5,6). Referral is included as a critical element in these programmes (7). In Ethiopia, the integrated community case management programme was initiated in 2010 (8). This programme trained health extension workers to assess, classify, and treat sick children 2-59 months of age with pneumonia, diarrhoea, malnutrition, and other common illnesses and refer severe cases to health centres according to guidelines (9). In 2013, the Community-Based Newborn Care programme was initiated. Health extension workers were trained to provide pre-referral antibiotic treatment for young infants (0-59 days) with possible severe bacterial infection and refer those to a higher-level facility. If a referral was not possible, the health workers were instructed to provide a seven-day antibiotic treatment. These interventions have been implemented in Ethiopia within the existing Health Extension Program (6,10).

A referral is defined as “a process in which a health worker at one level of the health system, having insufficient resources (drugs, equipment, skills) to manage a clinical condition, seeks the assistance of a better or differently resourced facility at the same or higher level to assist in or take over the management of the client’s case” (11). A well-functioning referral system is an essential part of the health system and an indicator of the quality of care provided at health facilities (7). The referral pattern may reflect the healthcare

For peer review only

workers' ability to identify signs of severe illness at an early stage (12). For effective referral care, a pre-requisite is also that the higher-level facility is ready to receive and treat these children appropriately (7).

In a previous study, we showed that the Ethiopian health extension workers referred only a fraction of the severely sick young infants and under-five children that deserved urgent referral to health centres. Generally, referral logistics were weak (13). Other studies in low-income countries have shown that the distance to the referral site, the severity of the child's illnesses (14), and the availability of referral logistics (15,16) may influence severely sick children's referral.

This study aimed to identify factors associated with the referral of severely ill children aged 0-59 months and healthcare providers' adherence to referral guidelines in Ethiopia's primary health care system. Specifically, we assessed the referral of sick children 0-59 months from health post to the next higher level of health care in relation to guidelines. We analysed factors associated with referral care, including child characteristics, health post preparedness, and distance to the next-level health facility.

METHODS

Study design and settings

This study was based on data from the evaluation of Optimizing the Health Extension Programme intervention. This intervention was a collaborative effort between the Government of Ethiopia and implementing partners to increase the use and quality of community-based newborn care and integrated community case management of childhood illnesses in four Ethiopian regions (17).

We did a secondary analysis of data from the endline cross-sectional survey conducted in 26 districts, where the Optimizing the Health Extension Programme intervention was implemented, and in 26 comparison districts without the intervention. These districts were found in four major Ethiopian regions, namely Amhara, Oromia, Southern Nations, Nationalities and Peoples (SNNP), and Tigray. The survey was conducted from December 2018 to February 2019, after the implementation of the intervention.

For peer review only

In Ethiopia, primary health care services are provided within the district health system, comprised of five primary health care units and a district hospital serving around 100,000 people. Each primary health care unit consists of five health posts and their referral health centre. The health post is typically staffed with two health extension workers and serves a population of 5000 people (18).

The referral care of sick children 0-59 months of age from health posts to the next level was assessed in relation to the treatment guidelines for 0-59 days old sick young infants (community-based newborn care), and 2 – 59 months old children (integrated community case management).

According to these guidelines, babies 0-59 days who are very preterm (< 32 weeks), very low birth weight (< 1500gm), preterm (32-37 weeks), low birth weight (1500-2500gm), have a possible severe bacterial infection, severe jaundice, severe dehydration, severe persistent diarrhoea, or dysentery require an urgent referral. Similarly, children 2-59 months with a possible severe bacterial infection, severe dehydration, severe pneumonia, possible severe bacterial infections, severe persistent diarrhoea, very severe febrile disease, severe complicated measles, mastoiditis, severe anaemia, and complicated severe acute malnutrition require an urgent referral (19).

Study population and sample

The primary study had household and health facility modules to evaluate the Optimizing the Health Extension Programme intervention (20). We used the 2007 Ethiopian census to select 200 enumeration areas proportional to the population size of the 52 districts included in the study. In the main study, households were randomly selected to represent the enumeration areas. The health posts serving the selected households were included in this study. All records of 0-59 days old sick young infants, who sought care in three months prior to the survey, and records of the most recent ten 2-59 months sick children, who also sought care, were reviewed from treatment registers at the health post. Children without any illness who visited the health posts for postnatal care, vaccination, and growth monitoring were excluded from the analysis.

For peer review only - http://bmjopen.bmj.com/site/about/guidelines.xhtml

The sample size for the main study was based on the requirement that the baseline and end-line surveys should measure changes of a fixed number of percentage points of selected indicators for care-seeking and treatment for a sick child, based on household interviews.

Data collection

The questionnaires used in this study were adapted from existing large-scale survey tools, such as the Demographic and Health Surveys and the Service Provision Assessment survey (21). Questionnaires were translated into three local languages (Amharic, Afan Oromo and Tigrigna) and pre-tested. All data were collected using tablet computers.

Out of the eligible 200 clusters, 19 clusters were excluded from the study due to civil unrest. Trained health professionals and supervisors collected the data.

We collected data on the number of health extension workers at the health posts, availability of amenities, equipment, supplies, diagnostics, and medicines. Geographic Positioning System (GIS) coordinates were assessed at health posts and referral health centres. We reviewed registers of 0-59 days and 2-59 months old children and collected background data on their symptoms, disease classifications, treatment, and referral status.

Patient and Public Involvement: Not applicable (patients were not involved in this study).

Data analysis

A descriptive analysis was performed, including frequencies, percentages, and means. We characterized the health posts, the distance from health posts to the referral health centres, the proportion of health posts with service readiness tracer items and referral logistics for childhood referral care, and the referral of sick children of 0-59 months from health posts to higher levels in relation to the treatment guidelines.

We used health posts and their respective referral health centres' GPS reading to calculate the two's straight-line distance. The health post's facility readiness was assessed using the World Health Organization service

For peer review only

availability and readiness assessment (SARA) reference manual (22). The general health facility service readiness has five domains, which include different tracer indicators, items, and services. The first is basic amenities, including four items: communication, access to toilet facilities for clients, water source, and power supply. The second is basic equipment with four items: infant or child scale, thermometer, functional stethoscope, and mid-upper arm circumference tape measure. The third is standard precautions for infection prevention with seven items: sharps container, chlorine bleach, bucket for decontamination solution, contaminated waste container, soap, and a towel or hand rub, alcohol-based hand rub, and clean gloves. The fourth is the basic diagnostic capacity with a rapid diagnostic test for malaria. The last is essential medicines with ten items: vitamin A, tetracycline eye ointment, gentamycin, amoxicillin, oral rehydration solution, zinc-oral rehydration solution combined, zinc, and the ready-to-use- therapeutic foods (plumpy nut and BP100). Finally, the combined facility service readiness was assessed using the general service readiness index by calculating five domains' mean score.

The attributes of the referred sick children, their symptoms, disease classification, and treatment were analysed based on the existing treatment guidelines (19). Accordingly, the proportion of sick children with severe illnesses that require an urgent referral, non-severe illnesses that do not require urgent referral, the numbers referred and causes of their referrals were analysed. The referral status was linked with the facility readiness, and other health post factors, including the distance to the referral facility.

Referral of 0-59 days old sick young infants, and 2-59 months old children were the outcomes separately dichotomized as "yes" (referred) or "no" (not referred). Chi-square tests were performed to assess whether the child's characteristics, health post preparedness, and the distance to the next level health facility were associated with a sick child's referral. Relevant factors with statistically significant association to the referral of sick children in the bivariate analysis were entered into a multivariable logistic regression analysis to identify referral predictors. Adjustments were made for clustering and whether the cluster was an Optimizing the Health Extension Program intervention or comparison area. Odds ratios (ORs) with 95% confidence intervals

For peer review only

(CIs) were calculated to measure the association between the predictor and outcome variables. Additionally, adherence to referral guidelines for sick young infants and older children was dichotomised as “yes” (managed according to referral guideline) or “no” (not managed according to referral guideline). Factors that had statistically significant association with referrals were then assessed in bivariate and multivariable logistic regression analyses to identify predictors of adherence to correct referral practices.

The Census and Survey Processing System (CSPro) software (United States Census Bureau, Washington DC, USA), was used to enter and edit survey data. Statistical analysis and tests were done using SPSS version 25 (SPSS, IBM Corp, New York, USA).

RESULTS

We extracted data from treatment registers at 165 health posts serving 181 enumeration areas. Information on the assessment, treatment and referral of 229 sick young infants of 0 – 59 days and 1123 sick children aged 2-59 months was collected from the registers. Out of the 165 health posts, 48 had registered to manage one or more 0-59 days old sick young infants, and 147 had registered one or more 2-59 months old sick children in the three months preceding the survey.

Characteristics of health posts

Nearly 80% of health posts were staffed with two or more health extension workers, while the rest had only one. Almost all health posts had sick child registers, and 42% had referred at least one sick child to a health centre in the quarter before the survey. A cell phone signal was available in most of the surveyed health posts. Still, most health workers had neither used mobile phones nor other communication means for their most recent referral of a sick child (Table 1). Half of the surveyed health posts were found within 10 kilometres from the next level referral health centre (Figure 1). There were missing distance data for seven health posts.

For peer review only

Health post readiness status

The readiness of health posts to provide health services was assessed for five general service readiness domains. The average readiness score was 61% for basic amenities, 80% for basic equipment, 49% for standard precaution for infection prevention, 50% for basic diagnostic capacity, and 52% for essential medicines (Figure 2). A significant proportion of the health posts lacked readiness to provide the required service for sick children, shown by an overall service readiness index of 57%. Moreover, nearly half of the health posts did not have the required essential medicines. Even amoxicillin, which is needed to treat possible severe bacterial infection of 0-59 days sick young infants, was not available in a quarter of the health posts. Rapid diagnostic tests, which are highly important in malaria endemic areas like Ethiopia, was not found in half of the health posts (Figure 2). The items included in these scores are shown in supplementary figure 1 – 4.

Referral of sick children

During the last three months before the survey, 39 (17%) of the 229 examined 0 – 59 days old sick young infants were referred from the health posts to the nearest health centres. Similarly, 78 (7%) of the 1123 sick children aged 2 – 59 months old had been referred. Possible serious bacterial infection and pneumonia were the leading causes of referral among 0 – 59 days old and 2 – 59 months old children, respectively (Table 2). Only 36% of 0–59-day sick young infants with possible severe bacterial infection were referred. Similarly, the health extension workers referred only 27% of 2-59 months old children with severe malnutrition (Table 2). Cross-tabulation of the four facility readiness domains and their respective individual components are shown in supplementary table 1 and 2, respectively.

Factors associated with referral

Sick young infants aged 0-59 days with severe illnesses were seven times more likely to be referred to the next referral level, as compared to those with non-severe illnesses (95% CI: 1.50 - 30.11) (Table 3). Sick young infants in the later weeks of life were less likely to be referred than those in the first week (AOR = 0.25,

For peer review only

95% CI: 0.10 - 0.64). Neither the sex of the sick child nor the distance from health post to referral health centre were associated with referral (Table 3). A higher proportion of sick young infants was referred from health posts served by two or more health extension workers than those served by only one (p=0.024) (Supplementary table 1).

Also, sick children aged 2-59 months with severe illness were more likely to be referred than those with non-severe illness (AOR = 21.27, 95% CI: 7.51 - 60.23) (Table 3). Health posts that had a better score for basic amenities were less likely to refer 2-59 months sick children to the next level than those without (AOR= 0.38, 95% CI: 0.15 – 0.96). Health posts with amoxicillin (AOR = 0.41, 95% CI: 0.19 - 0.88) and rapid diagnostic tests (AOR = 0.18, 95% CI: 0.07 – 0.46) were less likely to refer 2-59 months old children than those without these medicines and diagnostic kit.

The number of health extension workers, age and sex of the child and distance from health post to the referral health centre were not associated with referrals of 2-59 months old children.

Management according to guideline

The adherence to referral guidelines of sick young infants and older children is shown in table4. Among the sick 229 young infants, 21 children were referred when a referral was not advised, and 31 children were treated at the health post when a referral was recommended. The remaining 177 (77%) were managed according to referral guidelines. Of the factors associated with a referral, essential medicines were also associated with adhering to referral guidelines for young infants. Those in the higher tertile adhered more to referral guidelines (AOR= 13, 95% CI: 1.5-112.6). Among the 1123 sick older children, 56 were referred when a referral was not advised, and 22 children were treated at the health post when a referral was required. The remaining 945 (93%) were managed according to referral guidelines. Facilities with amoxicillin and diagnostics were more likely to adhere to referral guidelines (amoxicillin AOR = 2.8, 95% CI: 1.3-5.5 and diagnostics AOR = 4.7, 95% CI: 1.8-11.9). Data for this analysis are shown in tables 5 and 6.

For peer review only - http://bmjopen.bmj.com/site/about/guidelines.xhtml

DISCUSSION

Our study from four major Ethiopian regions showed that health extension workers referred only around one-third of the sick young infants 0-59 days old and half of the 2-59 months old children that should be referred, according to guidelines. The leading diagnoses at referral were possible severe bacterial infections in young infants and pneumonia in older children. As expected, conditions classified as severe were referred more frequently than non-severe cases. The tendency to refer was associated with some characteristics. For 0-59 days old infants, young neonates were referred more often than a bit older young infants. Health posts with basic amenities, amoxicillin, and rapid diagnostic tests were less inclined to refer older sick children. Within the 2-59 months group, age was not associated with referral. There was no sex difference in referral. Further, the straight-line distance from the health post to the health centre was not associated with referrals.

Due to the cross-sectional study design, we cannot prove any causal relationship between the health post and child factors that were associated with a referral of sick children. Further, we have analysed referral from the first primary care level, and are therefore only displaying part of the country's referral system. Exclusion of records of few sick children due to incomplete documentation on registers of some of the health facilities might be a limitation which could lead to reporting bias. We used objectively measured data on the health posts, based on the WHO health service availability and readiness assessment tool, and information obtained from the surveyed health posts were compared with national guidelines on referral. The study was performed in four of the largest regions in Ethiopia accounting for 80% of the population, where the enumeration areas were selected to represent the chosen districts of these regions. Though generalization of the study findings to the regions might be difficult, it is expected that the selected districts did not differ in health systems characteristics.

Despite the World Health Organization's recommendation (23), our study showed that only a third of young infants and half of the older children, who required referral, were referred from health post to the health centres. This finding was consistent with the previous Ethiopian and Malawian studies (9, 24). The low

For peer review only

referrals seen might be attributed to health providers' fear that caregivers would protest a referral (25), and the actual refusal of caregivers to comply with the referral (26). These factors might lead the health extension workers to manage the child at the health post (27). Health care providers might also undermine the overall impact of treatment guidelines on childhood survival (25, 28). They may perceive that there is little added value in referral and even feel a loss of power and prestige when referring (29). They might also lack the knowledge and be reluctant to adhere to the guidelines (29). This low level of referral of severely sick children is concerning. It could delay access to appropriate care in the higher-level referral facilities, thus increasing the risk of death (30), and hinder the efforts to reach the sustainable development goals on neonatal and under-five mortality (31).

In this study, the severity of a child's illness was associated with their referral in both age groups (32). This relation indicates that severely ill children were more frequently managed according to the referral guidelines. There is a need to strengthen health extension workers' ability to identify severely sick children requiring urgent referral (33). Neonates were primarily referred in their first week of life, highlighting the need for urgent referrals of severely ill newborns to prevent death in neonatal sepsis and other critical conditions (34).. Also, we found that distance from health posts to health centre was not associated with referrals, perhaps since nearly three-quarters of the health posts were located within 15 kilometres from their referral health centre.

Lack of the overall readiness of the health posts to provide the required childcare services (22) is an important finding of this study. This scenario could indicate that many care-seeking sick children were not getting the care they required, at least not at the health post (35). The lack of essential medicines might be a reason for some referrals of children with non-severe diseases, which otherwise may be treated at the first-level health facility (32, 36). This condition might also impose caregiver for unnecessary social and economic costs incurred by referrals of children with non-severe illnesses (36, 37).

For peer review only

Indeed, our data also showed that health posts that had medicine and diagnostics were likely to adhere to referral guidelines. This calls for the need of equipping the health facilities with the basic essential medicines and diagnostics. Although not statistically significant in the multivariable analysis, health posts with two or more health extension works were also more likely to refer as well as adhere to referral guidelines for young infants. A study in Niger also found adequate staffing to be linked to adherence to referral guidelines (29).

The non-adherence of the some of the healthcare providers to the referral guidelines when managing severely sick children found in this study requires improvement. This problem could result in overuse of antibiotics, and missing treatment or increased lethality in severe cases (38). Hence, mechanisms that can improve adherence to referral guidelines are needed as part of actions for improved quality-of-care.

Conclusion

We have shown that health extension workers referred few children with severe illnesses while a referral to the next-level health centre was recommended by guidelines. A significant proportion of the health posts were not ready to provide the necessary care, including referral. The severity of the child's illness, the availability of essential medicines and rapid diagnostic test were among the predictors of referrals of sick children from the health posts. Among other actions to enhance the quality of care provided at the health posts, improving medicine and diagnostic supplies may strengthen adherence to referral guidelines and ultimately prevent child deaths in Ethiopia.

Acknowledgments

We would like to extend our sincere gratitude to SNNP Regional Health Bureau and Hawassa University, College of Medicine and Health Sciences for providing support to conduct this research project. We would also like to pass our deepest appreciation to the different levels of the health systems and health providers involved in this study.

For peer review only

Authors' contributions

HB contributed to the conceptualization of the study, design, development of the survey instrument, training and supervision of data collectors, statistical analysis, and wrote the original draft and final versions of the manuscript. DK and HT contributed to the conceptualization of the study design, development of the study instruments, supervision of the data collection process, data analysis and review of the different versions of the manuscript. DB and LÅP contributed to the conceptualization of the study, design, development of the survey instrument, data compilation and statistical analysis, and reviewed the different versions of the paper. AD contributed to the management of distance from health post to health centre data, development of the study instruments, supervision of the data collection process, and review of the different versions of the manuscript. All authors read and approved the final version of the manuscript.

Funding: The study was supported by a grant from Bill and Melinda Gates Foundation to The London School of Hygiene and Tropical Medicine (grant OPP1132551). The funder had no role in data collection, analysis, or interpretation of results.

Disclaimer: Not required.

Competing interests: The authors declared no competing interests exist.

Patient consent for publication: Not required.

Ethics approval

Ethical approvals were secured from the ethical review boards of Hawassa University (protocol number IRB/199/10; June 2018), the Ethiopian Public Health Institute (protocol number SERO-012-8-2016; Version 001, August 2016) and the London School of Hygiene & Tropical Medicine (protocol number 11235; June 2016).

For peer review only

Data availability statement: Data from this study are co-owned by the participating institutions and stored in a depository at the Ethiopian Public Health Institute (EPHI). The use of these data is guided by a data sharing agreement. Data can be accessed from the secretary of the data sharing committee of EPHI-LSHTM collaborative projects. Contact information: Martha Zeweldemariam, E-mail: martha.zeweldemariam@lshtm.ac.uk

ORCID IDs

Habtamu Beyene https://orcid.org/0000-0003-0923-5191

Della Berhanu https://orcid.org/0000-0002-4984-893X

Atkure Defar http:// orcid.org/ 0000-0001-9435-2135

Lars Åke Persson https://orcid.org/0000-0003-0710-7954

For peer review only

References

1. United Nations Inter-agency Group for Child Mortality Estimation (UNIGME), 'Levels & Trends in Child Mortality: Report 2019, Estimates developed by the United Nations Inter-agency Group for Child Mortality Estimation', United Nations Children's Fund, New York, 2019.
2. Ruducha J, Mann C, Singh NS, et al. Articles How Ethiopia achieved Millennium Development Goal 4 through multisectoral interventions : a Countdown to 2015 case study. *Lancet Glob Heal.* 2015; 5 (11):e1142–51.
3. Ethiopian Public Health Institute (EPHI) [Ethiopia] and ICF. 2019. Ethiopia Mini Demographic and Health Survey 2019: Key Indicators. Rockville, Maryland, USA: EPHI and ICF. Available from: https://www.dhsprogram.com/pubs/pdf/PR120/PR120.pdf. Accessed on August 24, 2020.
4. Deribew A; Tessema GA; Deribe K; Melaku YA; Lakew Y; Amare AT; Abera SF; Mohammed M; Hiruye A; Teklay E; Misganaw A; Kassebaum N. Trends, causes, and risk factors of mortality among children under 5 in Ethiopia, 1990-2013: findings from the Global Burden of Disease Study 2013. *Popul Health Metr.* 2016; 14:42.
5. Mugeni, C., Levine, A. C., Munyaneza, R. M., Mulindahabi, E., Hannah, C., Habimana, J. P. …Ngabo, F. Nationwide implementation of integrated community case management of childhood illness in Rwanda. *Glob Heal Sci Pract.* 2014; *2*: 328–341.
6. Mathewos B, Owen H, Sitrin D, *et al*. Community-Based interventions for newborns in Ethiopia (COMBINE): cost-effectiveness analysis. *Health Policy Plan* 2017; 32:i21–32.
7. World Health Organisation. Standards for improving the quality of care for children and young adolescents in health facilities. Geneva: World Health Organization, 2018. CC BY-NC-SA 3.0 IGO.
8. Miller NP, Amouzou A, Tafesse M, Hazel E, Legesse H, Degefie T, et al. Integrated Community Case Management of Childhood Illness in Ethiopia : Implementation Strength and Quality of Care. *Am. J. Trop. Med. Hyg.* 2014; 91:424–34.

9. Amouzou A, Hazel E, Shaw B, Miller NP, Tafesse M, Mekonnen Y, et al. Effects of the integrated Community Case Management of Childhood Illness Strategy on Child Mortality in Ethiopia : A Cluster Randomized Trial. *Am J Trop Med Hyg.* 2016; 94:596–604.

10. Marsh DR, Hamer DH, Pagnoni F, Peterson S. Introduction to a Special Supplement : Evidence for the Implementation, Effects, and Impact of the Integrated Community Case Management Strategy to Treat Childhood Infection. *Am J Trop Med Hyg.* 2012; 87:2–5.

11. World Health Organization. Referral Systems - a summary of key processes to guide health services managers. Geneva: World Health Organization, 2013. Available at: https://www.who.int/management/facility/referral/en/. Accessed on August 10, 2020.

12. Price J, Willcox M, Kabudula CW, Herbst K, Hinton L, Kahn K, et al. Care pathways during a child's final illness in rural South Africa: Findings from a social autopsy study. *PLoS ONE* 2019; 14: e0224284.

13. Beyene H, Hailu D, Tadele H, Persson LÅ, Berhanu D. Insufficient referral practices of sick children in Ethiopia based on a cross-sectional survey. *Acta Paediatr.* 2020*; 00:1–8.*

14. Abrahim O, Linnander E, Mohammed H, Fetene N, Bradley E. A Patient-Centered Understanding of the Referral System in Ethiopian Primary Health Care Units. *PLoS One*. 2015; 10:1–10.

15. Eskandari M, Abbaszadeh A, Borhani F. Barriers of Referral System to Health Care Provision in Rural Societies in Iran. *J Caring Sci.* 2013; 2:229–36.

16. Akbari A, Mayhew A, Al-alawi MA, Grimshaw J, Winkens R, Glidewell E, et al. Interventions to improve outpatient referrals from primary care to secondary care. *Cochrane Database Syst Rev.* 2014; 4: 1-50.

17. Berhanu D, Okwaraji YB, Belayneh AB, *et al*. Protocol for the evaluation of a complex intervention aiming at increased utilisation of primary child health services in Ethiopia: a before and after study in intervention and comparison areas. *BMC Health Serv Res.* 2020; 20:339.

18. Global financing facility. Ethiopia Health Sector Transformation Plan (2015/16-2019/20). https://www.globalfinancingfacility.org/ethiopia-health-sector-transformation-plan-201516-201920. Accessed on November 22, 2020.

For peer review only

19. FMOH. Federal Ministry of Health, Ethiopia. *Integrated Management of Newborn and Childhood Illness, Part 1*. Blended Learning Module for the Health Extension Programme. 2011. Retrieved from https://www.open.edu/openlearncreate/-pluginfile.php/71990/-mod_resource/content/2/IMNCI-_Part_1_Final_Print-ready_March_2011_.pdf. Accessed on November 15, 2020.

20. Berhanu D, Okwaraji YB, Defar A, et al. Does a complex intervention targeting communities, health facilities and district health managers increase the utilisation of community- based child health services? A before and after study in intervention and comparison areas of Ethiopia. *BMJ Open* 2020; 10:e040868.

21. Central Statistical Agency (CSA) [Ethiopia] and ICF (2016). Ethiopia: Demographic and Health Survey 2016 [Internet]. 2016. Available from: https://www.dhsprogram.com/publications/-publication-FR328-DHS-Final-Reports.cfm. Accessed on November 22, 2020.

22. World Health Organization. Service availability and readiness assessment (SARA). An annual monitoring system for service delivery. Reference manual. Geneva; 2015. Available from http://www.who.int/healthinfo/systems/sara_indicators_questionnaire/en/. Accessed 20 October, 2020.

23. World Health Organization. Guideline: managing possible serious bacterial infection in young infants when referral is not feasible. World Health Organization. Geneva; 2015. https://apps.who.int/iris/-handle/10665/181426. Accessed 20 October, 2020.

24. Amouzou A, Kanyuka M, Hazel E, Heidkamp R, Marsh A, Mleme T, et al. Independent Evaluation of the integrated Community Case Management of Childhood Illness Strategy in Malawi Using a National Evaluation Platform Design. *Am J Trop Med Hyg.* 2016; 94:574–83.

25. Chinbuah MA, Abbey M, Kager PA, Gyapong M, Nonvignon J, Ashitey P, Akpakli J, Appiatse SA, Kubi D, Gyapong JO. Assessment of the adherence of community health workers to dosing and referral guidelines for the management of fever in children under 5 years: a study in Dangme West District, Ghana. *Int Health*. 2013; 5:148-56.

For peer review only

26. Hailegebriel TD, Mulligan B, Cousens S, Mathewos B, Wall S, Bekele A, et al. Effect on Neonatal Mortality of Newborn Infection Management at Health Posts When Referral Is Not Possible : *Glob Heal Sci Pract.* 2017; 5:202–16.

27. Wammanda RD, Adamu SA, Joshua HD, Nisar YB, Qazi SA, Aboubaker S, et al. Implementation of the WHO guideline on treatment of young infants with signs of possible serious bacterial infection when hospital referral is not feasible in rural Zaria, Nigeria: Challenges and solutions. *PLoS ONE*. 2020; 15: e0228718.

28. Wanduru P, Tetui M, Tuhebwe D, Ediau M, Okuga M, Nalwadda C, Ekirapa-Kiracho E, Waiswa P, Rutebemberwa E. The performance of community health workers in the management of multiple childhood infectious diseases in Lira, northern Uganda - a mixed methods cross-sectional study. *Glob Health Action.* 2016; 9:33194.

29. Bossyns P, Lerberghe W Van. Human Resources for Health The weakest link : competence and prestige as constraints to referral by isolated nurses in rural Niger. *BioMed Cent.* 2004; 8:1–8.

30. Nabongo P, Verver S, Nangobi E, Mutunzi R, Wajja A, Mayanja-kizza H, et al. Two year mortality and associated factors in a cohort of children from rural Uganda. *BMC Public Health.* 2014; 14:1–9.

31. Golding, N., Burstein, R., Longbottom, J., Browne, A. J., Fullman, N., Osgood-zimmerman, A., … Foundation, M. G. Mapping under-5 and neonatal mortality in Africa , 2000 – 15 : a baseline analysis for the Sustainable Development Goals. *Lancet*. 2015; *390*, 2171–2182.

32. Musa EO, Ejembi CL. Reasons and outcome of paediatric referrals from first- level health facilities in Sabongari , Zaria , Northwestern. *J Community Med Prim Heal Care.* 2004; 16:10–5.

33. Newbrander W, Ickx P, Werner R, Mujadidi F. Compliance with referral of sick children : a survey in five districts of Afghanistan. *BMC Pediatr.* 2012; 12:1471–2431.

34. Rosenstock S, Katz J, Mullany LC, Khatry SK, Leclerq SC, Darmstadt GL, et al. Sex differences in morbidity and care-seeking during the neonatal period in rural southern Nepal. *J Heal Popul Nutr*. 2015; 33:1–10.

35. Getachew T, Abebe SM, Yitayal M, Persson LÅ, Berhanu D. Assessing the quality of care in sick child services at health facilities in Ethiopia. *BMC Health Serv Res.* 2020; 7:1–12.

36. Logaraj, M. Management of Referral Services through Computer Based Management System, *Nat.J.Res.Com.Med.* 2013; 2, 1–4.

37. Nanyonjo A, Bagorogoza B, Kasteng F, Ayebale G, Makumbi F, Tomson G. Estimating the cost of referral and willingness to pay for referral to higher-level health facilities : a case series study from an integrated community case management programme in Uganda. *BMC Health Serv Res*. 2015;15:1–10.

38. Druetz T, Siekmans K, Goossens S. The community case management of pneumonia in Africa : a review of the evidence. Heal policy Planning. 2015;30(December 2013):253

For peer review only

Legend to figure

Figure 1: Cumulative percentages of distance from health posts to the referral health centres, analysed based on linear distance from 158 health posts to their 128 referral health centres.

Figure 2. The mean percentage scores for five general service readiness domains of the surveyed 165 health posts analysed based on the WHO Service Availability and Readiness Assessment (SARA) reference manual (22).

Supplementary figure 1: Percentages of mean score of basic amenities by tracer items

Supplementary figure 2: Percentages of mean score of basic equipment by tracer items

Supplementary figure 3: Percentages of mean score of standard precaution for infection prevention by tracer items.

Supplementary figure 4: Percentages of mean score of essential medicines by tracer items.

For peer review only

Table 1.Characteristics of health posts in four regions of Ethiopia (December 2018-February 2019) (n=165)

Characteristics	n	%
Region		
Amhara	70	42
Oromia	48	29
SNNPR[a]	22	13
Tigray	25	15
Number of health extension worker per health post		
1	35	21
2+	130	79
Availability of		
Registers for 0 – 59 days old children	148	90
Registers for 2 -59 months old children	156	95
Cell phone signals in the health post	135	82
Number of health posts that referred sick children in the last quarter	69	42
Means of communication used for the referral of last sick child		
Facility landline or mobile phone	3	2
Staff member's mobile phone	25	15
In person communication	13	8
No communication for last child referral	124	75

[a]SNNPR - Southern Nations, Nationalities, and Peoples Region

For peer review only

Table 2. Disease classification, recommended and performed referrals among children 0-59 months old seen at the surveyed 165 health posts, December 2018 – February 2019.

Age group	Disease classification	Referral guideline supports referral?	Number seen	Number (%) referred
0 – 59 days	Very preterm or very low birth weight	Yes	0	0
	Preterm or low birth weight	Yes	4	2 (50)
	Possible serious bacterial infection	Yes	42	15 (36)
	Severe dehydration	Yes	0	0
	Severe persistent diarrhea	Yes	1	0
	Dysentery	Yes	1	1 (100)
	Severe jaundice	Yes	1	0
	Local bacterial infection	No	59	5 (9)
	Some dehydration	No	2	1 (50)
	No dehydration	No	17	1 (6)
	Jaundice	No	1	0
	Malaria	No	0	0
	Feeding problem or low weight	No	45	2 (4)
	Classification not given	-	26	0
	Other[a]	No	30	12 (40)
	Total 0 – 59 days old		229	39 (17)
2 – 59 months	Severe pneumonia/very severe disease	Yes	9	8 (89)
	Severe dehydration	Yes	2	1 (50)
	Severe persistent diarrhea	Yes	3	1 (33)
	Dysentery	Yes	9	6 (67)
	Very severe febrile disease	Yes	2	1 (50)
	Severe complicated measles	Yes	0	0
	Severe malnutrition	Yes	22	6 (27)
	Severe anaemia	Yes	0	0
	Pneumonia	No	478	16 (3)
	Some dehydration	No	186	9 (5)
	No dehydration	No	203	0
	Persistent diarrhea	No	28	2 (7)
	Malaria	No	12	0
	Fever, malaria unlikely	No	11	0
	Fever, no malaria	No	55	4 (7)
	Measles with eye/mouth complications	No	0	0

For peer review only

Age group	Disease classification	Referral guideline supports referral?	Number seen	Number (%) referred
	Measles	No	1	1 (100)
	Acute ear infection	No	7	3 (43)
	Chronic ear infection	No	1	0
	Moderate malnutrition	No	33	4 (12)
	Anaemia	No	1	0
	Others[b]	No	64	16 (25)
	Total 2 – 59 months old		1123	78 (7)

[a]Other – classification for 0 – 59 days included “infection unlikely” (12), “eye problems” (10), and PNC cases with referral outcome (8)

[b]Others – classification for 2 – 59 months old children included mainly “no pneumonia, cough” (42), and “eye problems” (6), growth monitoring with referral outcomes (16).

For peer review only

Table 3. Factors associated with referral of 0-59 days old infants and 2-59 months old children from health post to higher levels in four Ethiopian regions, December 2018-February 2019. Logistic regression analyses.

Characteristic	Levels	Referred (n)	Not referred (n)	Crude OR (95% CI)	Adjusted OR (95% CI)[1]
**For 0-59 days old infants (N=228)[2]**					
The health post					
Facility owned landline or mobile phone	Not available	13	35	1.0	1.0
	Available	26	154	0.47 (0.17 – 1.28)	0.61 (0.13 – 2.80)
Mean score of essential medicine	0 to 33 percent	1	11	1.0	1.0
	34 to 66 percent	32	112	3.14 (0.99 - 9.99)	3.17 (0.42 - 24.19)
	67 to 100 percent	6	66	1.00 (0.24 – 4.14)	1.34 (0.10 – 18.80)
Number of health extension workers	1	2	39	1.0	1.0
	2 or more	37	150	4.81 (1.10 - 21.05)	5.63 (0.87 – 36.40)
Distance between health post and the nearest referral health centre					
	0-8 km	14	60	1.0	1.0
Distance tertiles	8.01-20 km	11	63	0.54 (0.18 – 1.60)	0.60 (0.18 - 2.04)
	20.01 or more km	14	66	0.87 (0.26 – 2.96)	1.02 (0.33 – 3.17)
The child					
Age	0-1 week	14	45	1.0	1.0
	2-4 weeks	16	82	0.63 (0.21 – 1.84)	0.35 (0.11 – 1.10)
	5 or more weeks	9	62	0.47 (0.15 - 1.46)	0.25 (0.10 - 0.64)
Gender	Boy	18	105	1.0	1.0
	Girl	21	84	1.46 (0.59 - 3.62)	1.12 (0.45 - 2.75)
Disease classification	Not severe	21	158	1.0	1.0

For peer review only

Characteristic	Levels	Referred (n)	Not referred (n)	Crude OR (95% CI)	Adjusted OR (95% CI)[1]
	Severe	18	31	4.37 (1.30 – 14.67)	6.72 (1.50 - 30.11)
**For 2-59 months old children (N=1074)[3]**					
The health post					
Facility owned landline or mobile phone	Not available	44	792	1.0	1.0
	Available	27	211	2.30 (1.16 – 4.55)	1.88 (0.94 – 3.76)
Mean score of basic amenities	0 to 33 percent	20	129	1.0	1.0
	34 to 66 percent	15	279	0.35 (0.13 – 0.89)	0.38 (0.15 – 0.96)
	67 to 100 percent	36	595	0.39 (0.17 – 0.89)	0.40 (0.16 - 1.03)
Mean score of basic equipment	0 to 33 percent	2	64	1.0	1.0
	34 to 66 percent	12	147	2.61 (0.65 - 10.45)	3.49 (0.16 - 75.12)
	67 to 100 percent	57	792	2.30 (0.62 - 8.53)	2.38 (0.12 - 48.96)
Amoxicillin	Not available	27	152	1.0	1.0
	Available	44	851	0.29 (0.15 – 0.58)	0.41 (0.19 - 0.88)
Rapid diagnostic test for malaria	Not available	45	449	1.0	1.0
	Available	26	554	0.47 (0.26 – 0.83)	0.18 (0.07 - 0.46)
Mean score of overall service readiness[3]	0 to 33 percent	6	22	1.0	1.0
	34 to 66 percent	43	573	0.28 (0.04 – 2.05)	0.21 (0.03 – 1.53)
	67 to 100 percent	22	408	0.20 (0.03 – 1.49)	0.62 (0.07 – 5.60)
Number of health extension workers	1	20	196	1.0	1.0
	2 or more	51	807	0.62 (0.30 – 1.28)	1.01 (0.38 - 2.65)
Distance between health post and the nearest referral health centre					

For peer review only

Characteristic	Levels	Referred (n)	Not referred (n)	Crude OR (95% CI)	Adjusted OR (95% CI)[1]
Distance tertiles	0-8 km	24	321	1.0	1.0
	8.01-14 km	25	320	1.18 (0.54 - 2.56)	0.98 (0.43 – 2.21)
	14.01 or more km	22	362	0.86 (0.43 - 1.71)	0.71 (0.28 - 1.79)
The child					
Age	0-1 week	25	284	1.0	1.0
	2-4 weeks	24	317	0.86 (0.48 - 1.54)	0.97 (0.52 - 1.81)
	5 or more weeks	22	402	0.62 (0.36 - 1.07)	0.88 (0.45 - 1.68)
Gender	Boy	35	526	1.0	1.0
	Girl	36	477	1.13 (0.71 -1.81)	0.89 (0.52 - 1.52)
Disease classification	Not severe	50	981	1.0	1.0
	Severe	21	22	18.73 (9.21 – 38.09)	21.27 (7.51 - 60.23)

[1] Multivariable logistic regression adjusted for region and clusters. The logistic regression model was tested using the Hosmer and Lemeshow test of the goodness-of-fit statistics, which suggested that the model was a good fit to the data.
[2]Records of 1 sick young infant of 0-59 days old were removed because of missing of data of distance from health post to the referral health centre.
[3]Records of 49 sick older children of 2-59 months old were removed because of missing of data of distance from health post to the referral health centre.

For peer review only

Table 4. Adherence to referral management guidelines for sick young infants and older children

Age group	Observed	Management guideline	
		Referral advised	Referral not advised
0-59 days	Referred	18	21
	Not referred	31	159
2-59 months	Referred	22	56
	Not referred	22	1023

*Shadowed cells indicate referral management that did not adhere to guidelines

For peer review only

Table 5: Factors associated with adherence to referral guidelines in the management of 0-59 days old sick young infants

Characteristic	Levels	Management according to guideline		Crude OR (95% CI)	Adjusted OR (95% CI)
		Correct (n)	Not correct (n)		
Facility-owned landline or mobile phone	Not available	145	42	1.0	1.0
	Available	31	10	0.90 (0.26 – 3.07)	1.12 (0.27 – 4.68)
Mean score of essential medicine	0 to 33 percent	7	5	1.0	1.0
	34 to 66 percent	112	32	2.50 (0.83 – 7.51)	4.49 (0.93 – 21.77)
	67 to 100 percent	57	15	2.71 (0.51 – 14.46)	12.95 (1.49 – 112.82)
Number of health extension workers	1	27	14	1.0	1.0
	2 or more	149	38	2.03 (0.37 – 11.28)	8.51 (0.83 – 86.98)
Distance tertiles	0-8 km	54	23	1.0	1.0
	8.01-20 km	63	18	1.49 (0.37 – 6.03)	1.01 (0.17 – 6.13)
	20.01 or more km	59	11	2.28 (0.56 – 9.30)	0.58 (0.08 – 4.20)
Age	0-1 week	49	10	1.0	1.0
	2-4 weeks	69	29	0.49 (0.15 – 1.58)	0.57 (0.15 – 2.24)
	5 or more weeks	58	13	0.91 (0.26 – 3.23)	1.37 (0.28 - 6.76)
Gender	Boy	95	28	1.0	1.0
	Girl	81	24	0.99 (0.47 – 2.11)	1.19 (0.55 - 2.58)

For peer review only

Table 6: Factors associated with adherence to referral guidelines in the management of 2-59 months old sick children

Characteristic	Levels	Management according to guideline		Crude OR (95% CI)	Adjusted OR (95% CI)
		Correct (n)	Not correct (n)		
Age	0-1 week	282	27	1.0	1.0
	2-4 weeks	315	26	1.16 (0.65 – 2.06)	1.24 (0.70 – 2.18)
	5 or more weeks	402	22	1.75 (0.95 – 3.23)	1.78 (0.93 - 3.40)
Gender	Boy	524	37	1.0	1.0
	Girl	475	38	0.88 (0.56 – 1.39)	0.97 (0.61 - 1.55)
Amoxicillin	Not available	153	26	1.0	1.0
	Available	846	49	2.93 (1.48 – 5.82)	2.74 (1.40 – 5.35)
Distance tertiles	0-8 km	340	24	1.0	1.0
	8.01-14 km	298	28	0.75 (0.36 – 1.58)	0.72 (0.28 – 1.82)
	14.01 or more km	361	23	1.11 (0.54 – 2.27)	0.96 (0.44 – 2.05)
Mean score of basic amenities	0 to 33 percent	130	19	1.0	1.0
	34 to 66 percent	278	16	2.54 (1.01 – 6.39)	1.97 (0.71 – 5.49)
	67 to 100 percent	591	40	2.16 (0.93 – 5.02)	1.41 (0.56 – 3.56)
Mean score of basic equipment	0 to 33 percent	61	5	1.0	1.0
	34 to 66 percent	141	18	0.64 (0.21 – 1.93)	0.96 (0.12 – 7.57)
	67 to 100 percent	797	52	1.26 (0.45 – 3.47)	2.46 (0.29 – 20.54)
Mean score of diagnostics	0 to 33 percent	448	46	1.0	1.0
	67 to 100 percent	551	29	1.95 (1.10 – 3.46)	4.59 (1.79 - 11.75)
Mean score of overall readiness	0 to 33 percent	21	7	1.0	1.0
	34 to 66 percent	570	46	4.13 (0.68 – 25.02)	3.19 (0.43 - 23.61)
	67 to 100 percent	408	22	6.18 (1.01 – 37.87)	1.59 (0.14 – 18.00)
Number of health extension workers	1	193	23	1.0	1.0
	2 or more	806	52	1.84 (0.92 – 3.72)	1.52 (0.77 – 3.02)

For peer review only

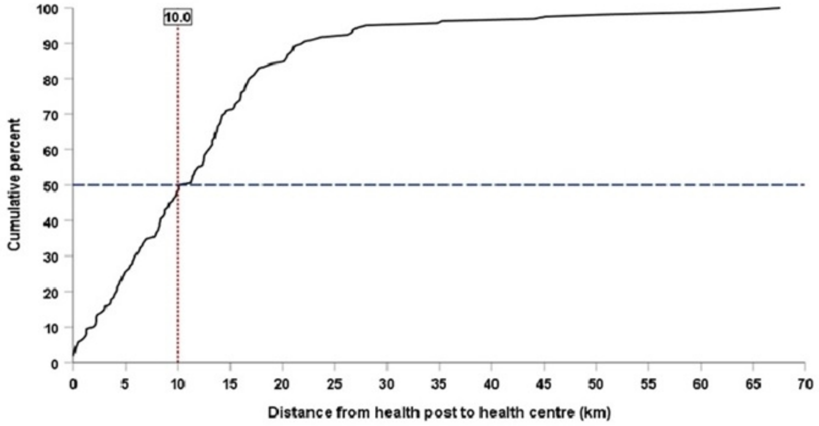

Figure 1: Cumulative percentages of distance from health posts to the referral health centres, analysed based on linear distance from 158 health posts to their 128 referral health centres.

216x121mm (96 x 96 DPI)

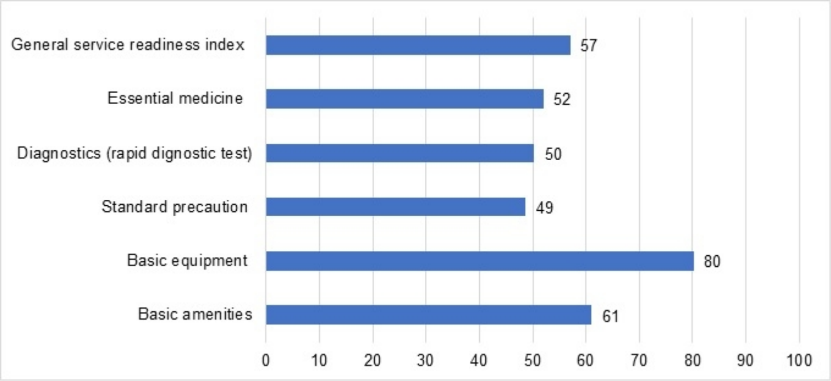

Figure 2. The mean percentage scores for five general service readiness domains of the surveyed 165 health posts analysed based on the WHO Service Availability and Readiness Assessment (SARA) reference manual (22).

216x121mm (96 x 96 DPI)

List of supplementary figures

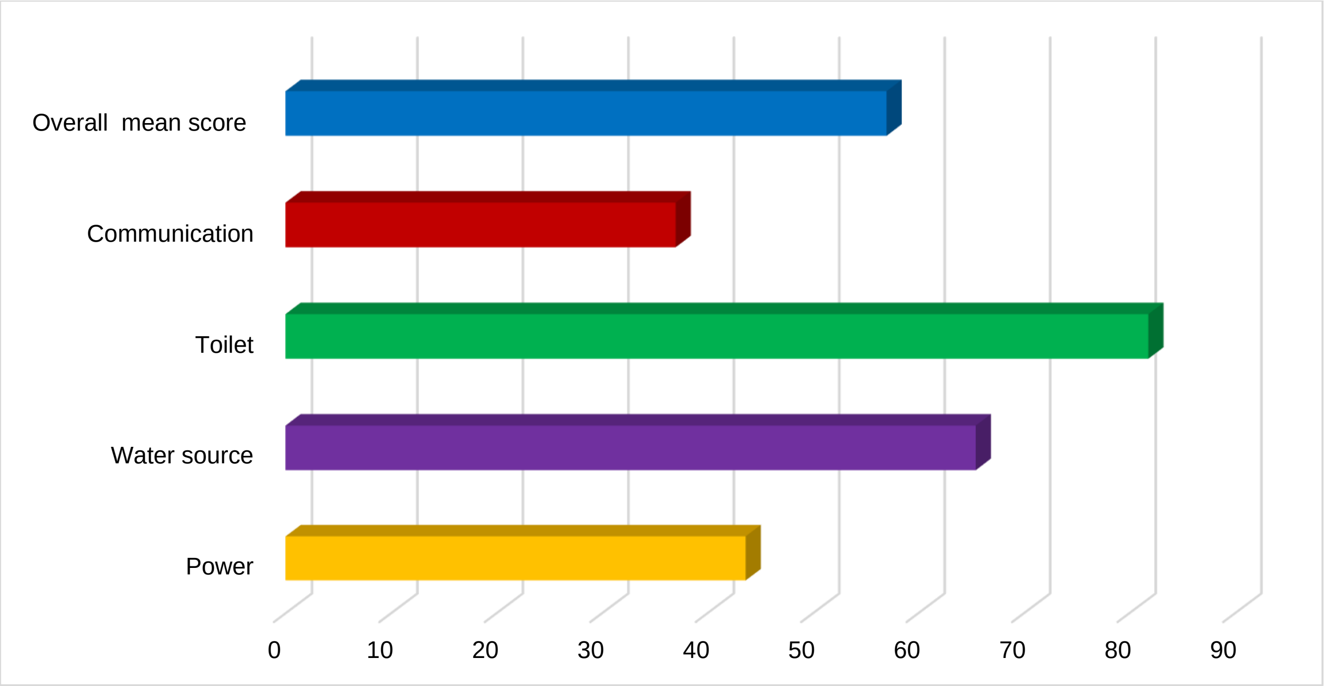

Supplementary figure 1: Percentages of mean score of basic amenities by tracer items

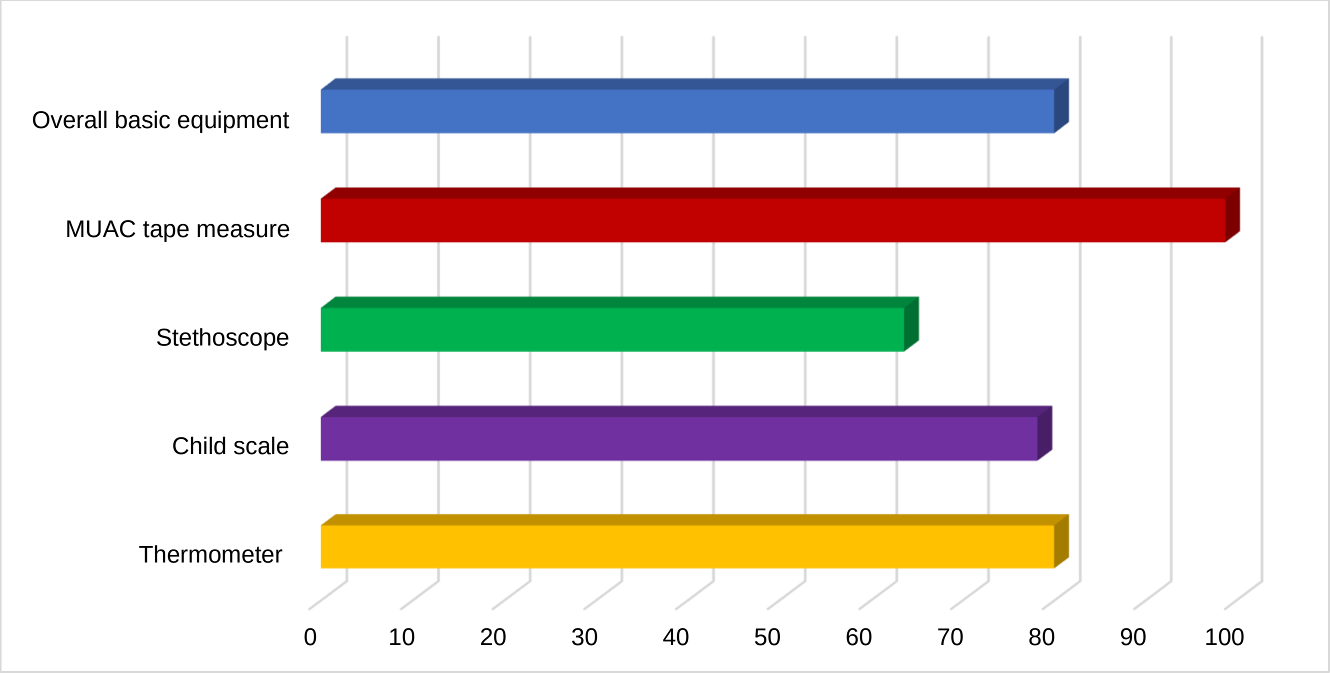

Supplementary figure 2: Percentages of mean score of basic equipment by tracer items

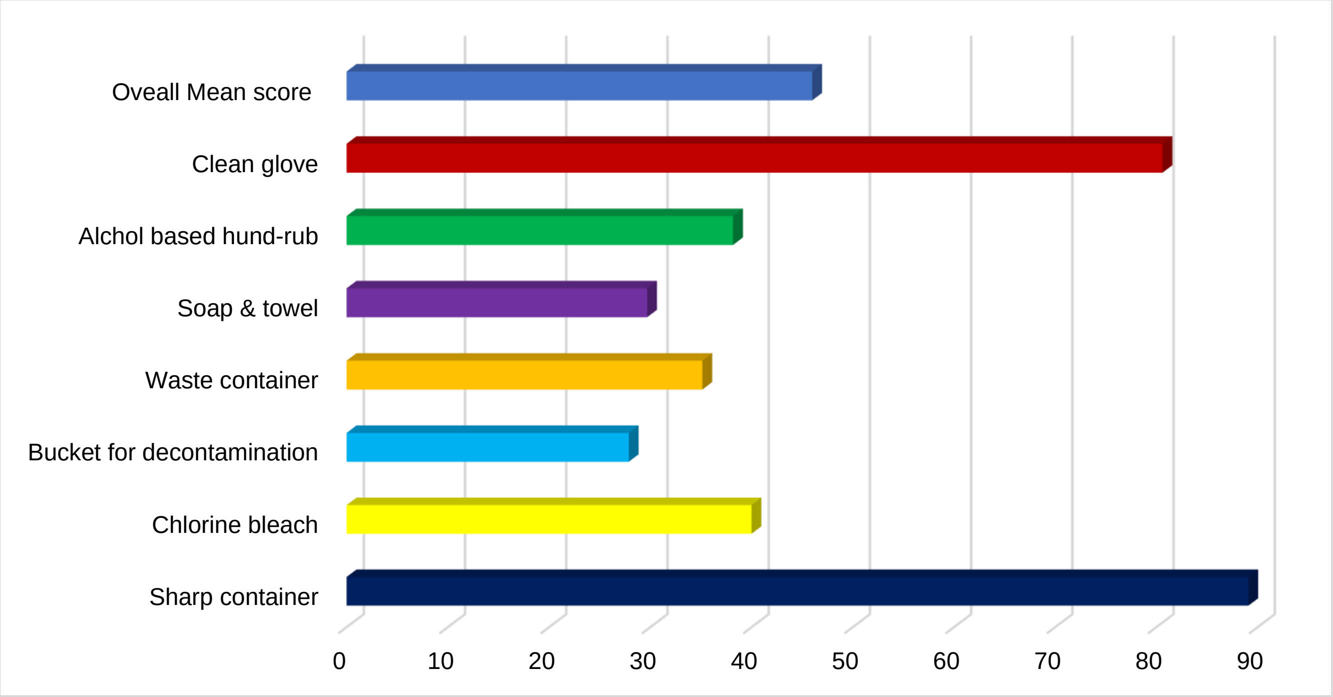

Supplementary figure 3: Percentages of mean score of standard precaution for infection prevention by tracer items.

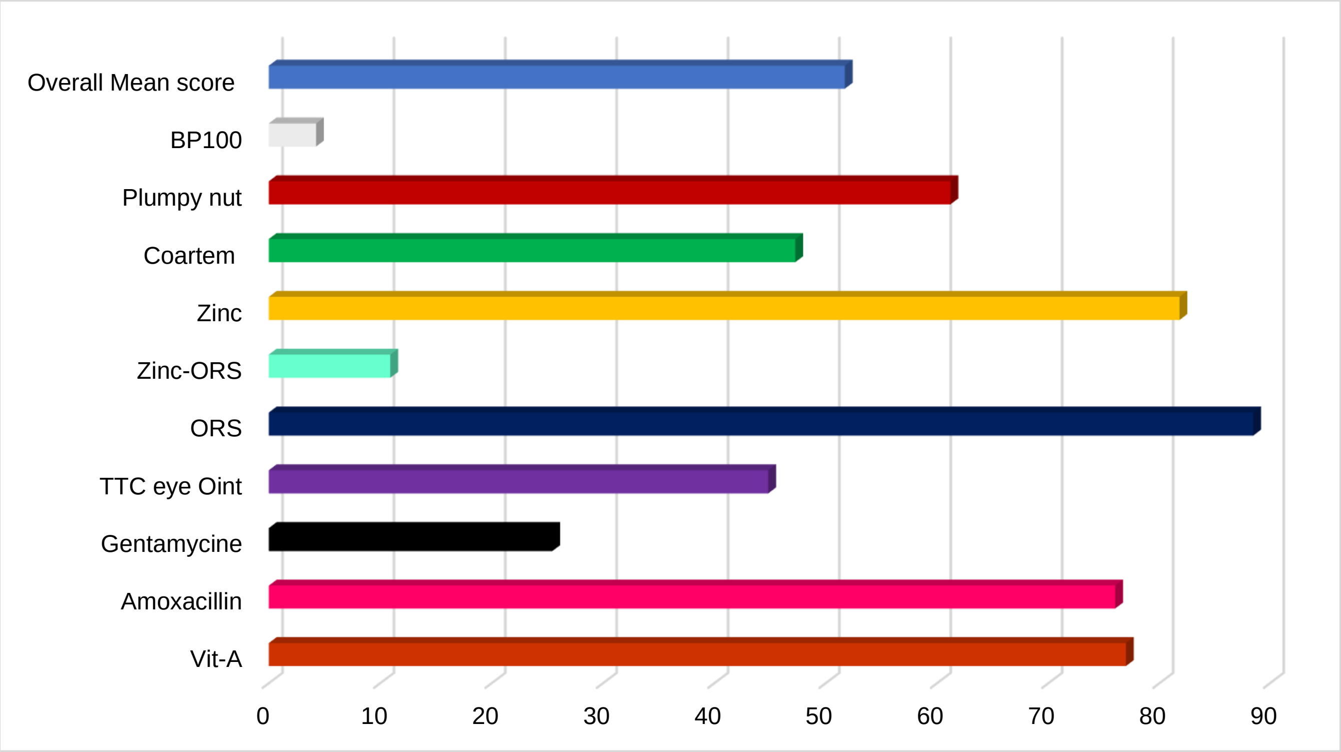

Supplementary figure 4: Percentages of mean score of essential medicines by tracer items.

List of Supplementary tables

Supplementary table 1: Health post and child factors associated with referrals of 0 – 59 months sick young children in four regions of Ethiopia

Characteristics	Referral status of 0 – 59 days old sick young infants			Referral status of 2 – 59 months old children		
	Referred N=39 n (%)	Not referred N=190 n (%)	P-value	Referred N=78 n (%)	Not referred N=1045 n (%)	P-value
The health post						
Mean score of basic amenities						
0 to 33 percent	13 (24)	42 (76)	0.466	20 (13)	129 (87)	
34 to 66 percent	9 (15)	50 (85)		19 (6)	292 (94)	0.043
67 to 100 percent	17 (15)	98 (85)		39 (6)	624 (94)	
Mean score of basic equipment						
0 to 33 percent	2 (22)	7 (78)		3 (4)	73 (96)	
34 to 66 percent	10 (19)	43 (81)	0.825	12 (8)	147 (92)	0.512
67 to 100 percent	27 (16)	140 (84)		63 (7)	825 (93)	
Mean score of standard precaution						
0 to 33 percent	4 (9)	40 (91)		30 (8)	353 (92)	
34 to 66 percent	14 (19)	59 (81)	0.527	31 (8)	355 (92)	0.279
67 to 100 percent	21 (19)	91 (81)		17 (5)	337 (95)	
Rapid diagnostic test (RDT)						
0 to 33 percent	10 (17)	50 (83)		47 (9)	467 (91)	
34 to 66 percent	0	0	0.947	0	0	0.026
67 to 100 percent	29 (17)	140 (83)		31 (5)	578 (95)	
Mean score of essential medicines						
0 to 33 percent	1 (8)	11 (92)		17 (18)	77 (82)	
34 to 66 percent	32 (21)	113 (79)	0.026	43 (6)	697 (94)	0.001
67 to 100 percent	6 (8)	66 (92)		18 (6)	271 (94)	
Health post's overall service readiness index						
0 to 33 percent	0 (0)	1 (0)		7 (18)	31 (82)	
34 to 66 percent	22 (19)	93 (81)	0.737	43 (7)	575 (93)	0.193
67 to 100 percent	17 (15)	96 (85)		28 (6)	439 (94)	
Number of health extension worker per health post						

For peer review only

1	2 (5)	39 (95)	0.024	20 (9)	196 (81)	0.264
2 or more	37 (20)	151 (80)		58 (6)	849 (94)	
Distance between health post and the nearest referral health centre						
Distance to the next referral site[b]						
0 - 8 kms	16 (21)	61 (79)	0.554			
8.01 - 20 kms	10 (12)	71 (88)				
>20 kms	13 (19)	57 (81)				
0 - 8 kms				24 (7)	340 (93)	0.699
8.01 - 14 kms				25 (8)	301 (92)	
>14 kms				22 (6)	362 (94)	
The child						
Region						
Amhara	3 (25)	9 (75)	0.722	34 (7)	428 (93)	0.691
Oromia	10 (13)	65 (87)		26 (7)	342 (93)	
SNNPR[a]	9 (21)	33 (79)		11 (8)	128 (92)	
Tigray	17 (17)	83 (83)		7 (5)	147 (95)	
Age						
1st week	14 (24)	45 (76)	0.353			
2 – 4 weeks	16 (16)	82 (84)				
5 – 8 weeks	9 (13)	63 (87)				
2 – 11 months				26 (8)	294 (92)	0.249
12 – 23 months				28 (8)	325 (92)	
24 – 59 months				24 (5)	426 (95)	
Sex						
Boy	18 (15)	106 (85)	0.395	40 (7)	550 (93)	0.810
Girl	21 (20)	84 (80)		38 (7)	495 (93)	
Disease classification						
Non-severe illnesses not requiring referral	21 (12)	159 (88)	0.013	56 (5)	1023 (95)	0.001
Severe illnesses requiring referral	18 (37)	31 (63)		22 (50)	22 (50)	

[a] SNNPR - Southern Nations, Nationalities, and Peoples Region

[b] GIS based data of distance from health post to health centre were missing for 0-59 days sick young infant (1) and 2-59 months sick children (49).

Supplementary table 2: Individual health post readiness factors associated with referrals of 0-59 months sick children in four regions of Ethiopia

Characteristics		Referral status of 0 – 59 days sick young infants			Referral status of 2 – 59 months old children		
		Referred N=39 n (%)	Not referred N=190 n (%)	P-value	Referred N=78 n (%)	Referred N=1045 n (%)	P-value
Availability of basic amenities							
Water source							
	Not available	15 (22)	53 (78)	0.296	30 (8)	356 (92)	0.558
	Available	24 (15)	137 (85)		48 (7)	689 (93)	
Electric power							
	Not available	18 (17)	91 (83)	0.886	51 (8)	602 (92)	0.257
	Available	21 (18)	99 (82)		27 (6)	443 (94)	
Facility owned toilet							
	Not available	6 (18)	27 (82)	0.880	14 (7)	207 (93)	0.981
	Available	33 (17)	163 (83)		64 (7)	838 (93)	
Facility owned landline or mobile phone							
	Not available	35 (19)	153 (81)	0.133	51 (6)	828 (94)	0.038
	Available	4 (10)	37 (90)		27 (11)	217 (89)	
Availability of basic equipment							
Thermometer							
	Not available	8 (14)	49 (86)	0.594	12 (5)	228 (95)	0.207
	Available	31 (18)	141 (82)		66 (8)	817 (92)	
Child weight scale							
	Not available	5 (13)	34 (87)	0.526	20 (9)	214 (91)	0.388
	Available	34 (18)	156 (82)		58 (7)	831 (93)	
Stethoscope							
	Not available	18 (19)	79 (81)	0.709	35 (8)	399 (92)	0.336
	Available	21 (16)	111 (84)		43 (6)	646 (94)	
MUAC tape measure							
	Not available	1 (100)	0	0.040	0 (0)	14 (100)	0.701
	Available	38 (17)	190 (83)		78 (7)	1031 (93)	
Availability diagnostics							
Rapid diagnostic test (RDT)							
	Not available	10 (17)	50 (84)	0.947	47 (9)	467 (91)	0.026
	Available	29 (17)	140 (82)		31 (5)	578 (95)	

For peer review only

Availability of essential medicines							
Vitamin A							
	Not available	9 (27)	25 (73)	0.211	11 (5)	207 (95)	0.214
	Available	30 (15)	165 (85)		67 (7)	838 (93)	
Amoxicillin							
	Not available	6 (14)	36 (86)	0.745	28 (15)	159 (85)	0.001
	Available	33 (18)	154 (82)		50 (5)	886 (95)	
Gentamycin							
	Not available	20 (14)	127 (86)	0.201			
	Available	19 (23)	63 (77)				
Tetracycline eye ointment							
	Not available	26 (19)	110 (81)	0.457	46 (8)	567 (92)	0.554
	Available	13 (14)	80 (86)		32 (6)	478 (94)	
Oral rehydration solution (ORS)							
	Not available	2 (13)	13 (87)	0.627	10 (17)	48 (83)	0.048
	Available	37 (17)	177 (83)		68 (6)	997 (94)	
Zinc-ORS							
	Not available				69 (7)	947 (93)	0.608
	Available				9 (8)	98 (92)	
Zinc							
	Not available				16 (9)	152 (91)	0.331
	Available				62 (7)	893 (93)	
Coartem							
	Not available	18 (20)	71 (80)	0.494	47 (9)	507 (91)	0.087
	Available	21 (15)	119 (85)		31 (5)	535 (95)	
Plumpy Nut							
	Not available	8 (23)	27 (77)	0.404	30 (8)	372 (92)	0.688
	Available	31 (16)	163 (84)		48 (7)	673 (93)	
BP100							
	Not available	38 (17)	180 (83)	0.012	77 (7)	993 (93)	0.107
	Available	1 (9)	10 (91)		1 (2)	52 (98)	

For peer review only

STROBE 2007 (v4) Statement—Checklist of items that should be included in reports of *cross-sectional studies*

Section/Topic	Item #	Recommendation	Reported on page #
**Title and abstract**	1	(*a*) Indicate the study's design with a commonly used term in the title or the abstract	1
		(*b*) Provide in the abstract an informative and balanced summary of what was done and what was found	2
**Introduction**			
Background/rationale	2	Explain the scientific background and rationale for the investigation being reported	3
Objectives	3	State specific objectives, including any prespecified hypotheses	5
**Methods**			
Study design	4	Present key elements of study design early in the paper	5
Setting	5	Describe the setting, locations, and relevant dates, including periods of recruitment, exposure, follow-up, and data collection	5
Participants	6	(*a*) Give the eligibility criteria, and the sources and methods of selection of participants	6
Variables	7	Clearly define all outcomes, exposures, predictors, potential confounders, and effect modifiers. Give diagnostic criteria, if applicable	8
Data sources/ measurement	8*	For each variable of interest, give sources of data and details of methods of assessment (measurement). Describe comparability of assessment methods if there is more than one group	7
Bias	9	Describe any efforts to address potential sources of bias	8
Study size	10	Explain how the study size was arrived at	6 – 7
Quantitative variables	11	Explain how quantitative variables were handled in the analyses. If applicable, describe which groupings were chosen and why	7 – 8
Statistical methods	12	(*a*) Describe all statistical methods, including those used to control for confounding	8
		(*b*) Describe any methods used to examine subgroups and interactions	8 - 9
		(*c*) Explain how missing data were addressed	29 - 30
		(*d*) If applicable, describe analytical methods taking account of sampling strategy	8
		(*e*) Describe any sensitivity analyses	-
**Results**			

For peer review only

Participants	13*	(a) Report numbers of individuals at each stage of study—eg numbers potentially eligible, examined for eligibility, confirmed eligible, included in the study, completing follow-up, and analysed	9
		(b) Give reasons for non-participation at each stage	-
		(c) Consider use of a flow diagram	-
Descriptive data	14*	(a) Give characteristics of study participants (eg demographic, clinical, social) and information on exposures and potential confounders	9
		(b) Indicate number of participants with missing data for each variable of interest	8, 28, 29, 30
Outcome data	15*	Report numbers of outcome events or summary measures	10, 25
Main results	16	(*a*) Give unadjusted estimates and, if applicable, confounder-adjusted estimates and their precision (eg, 95% confidence interval). Make clear which confounders were adjusted for and why they were included	11, 29, 30
		(*b*) Report category boundaries when continuous variables were categorized	10, 29, 30
		(*c*) If relevant, consider translating estimates of relative risk into absolute risk for a meaningful time period	N/A
Other analyses	17	Report other analyses done—eg analyses of subgroups and interactions, and sensitivity analyses	12
**Discussion**			
Key results	18	Summarise key results with reference to study objectives	12
Limitations	19	Discuss limitations of the study, taking into account sources of potential bias or imprecision. Discuss both direction and magnitude of any potential bias	12 - 13
Interpretation	20	Give a cautious overall interpretation of results considering objectives, limitations, multiplicity of analyses, results from similar studies, and other relevant evidence	13 - 14
Generalisability	21	Discuss the generalisability (external validity) of the study results	13
**Other information**			
Funding	22	Give the source of funding and the role of the funders for the present study and, if applicable, for the original study on which the present article is based	16

*Give information separately for cases and controls in case-control studies and, if applicable, for exposed and unexposed groups in cohort and cross-sectional studies.

Note: An Explanation and Elaboration article discusses each checklist item and gives methodological background and published examples of transparent reporting. The STROBE checklist is best used in conjunction with this article (freely available on the Web sites of PLoS Medicine at http://www.plosmedicine.org/, Annals of Internal Medicine at http://www.annals.org/, and Epidemiology at http://www.epidem.com/). Information on the STROBE Initiative is available at www.strobe-statement.org.

For peer review only
